# Deciphering Design of Aggregation‐Induced Emission Materials by Data Interpretation

**DOI:** 10.1002/advs.202411345

**Published:** 2024-11-22

**Authors:** Junyi Gong, Ziwei Deng, Huilin Xie, Zijie Qiu, Zheng Zhao, Ben Zhong Tang

**Affiliations:** ^1^ School of Science and Engineering, Shenzhen Institute of Aggregate Science and Technology The Chinese University of Hong Kong, Shenzhen (CUHK‐SZ) 2001 Longxiang Road, Longgang District Shenzhen Guangdong 518172 P. R. China; ^2^ Faculty of Chemistry Shenzhen MSU‐BIT University Longgang Shenzhen Guangdong 518172 P. R. China; ^3^ Department of Chemistry, Hong Kong Branch of Chinese National Engineering Research Center for Tissue Restoration and Reconstruction Institute of Molecular Functional Materials Division of Life Science and State Key Laboratory of Molecular Neuroscience The Hong Kong University of Science and Technology Clear Water Bay Kowloon Hong Kong SAR P. R. China

**Keywords:** aggregation‐induced emission, data interpretatio, photophysics

## Abstract

This work presents a novel methodology for elucidating the characteristics of aggregation‐induced emission (AIE) systems through the application of data science techniques. A new set of chemical fingerprints specifically tailored to the photophysics of AIE systems is developed. The fingerprints are readily interpretable and have demonstrated promising efficacy in addressing influences related to the photophysics of organic light‐emitting materials, achieving high accuracy and precision in the regression of emission transition energy (mean absolute error (*MAE*) ∼ 0.13*eV*) and the classification of optical features and excited state dynamics mechanisms (*F*1*score* ∼ 0.94). Furthermore, a conditional variational autoencoder and integrated gradient analysis are employed to examine the trained neural network model, thereby gaining insights into the relationship between the structural features encapsulated in the fingerprints and the macroscopic photophysical properties. This methodology promotes a more profound and thorough comprehension of the characteristics of AIE and guides the development strategies for AIE systems. It offers a solid and overarching framework for the theoretical analysis involved in the design of AIE‐generating compounds and elucidates the optical phenomena associated with these compounds.

## Graphical abstract

Employing data science to elucidate the relationship between structure and properties of AIE materials in a comprehensible manner.

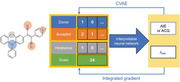

## 1 Introduction

Organic fluorescent dyes are essential in various domains, including bioimaging agents, photovoltaic cells, and optoelectronic devices.^[^ ^1^, ^2^, ^3^ ^]^ In many applications, it is imperative for these materials to exhibit high emission efficiency in condensed forms, such as crystalline structures or nanoparticles. However, conventional fluorescent systems often experience the detrimental aggregation‐caused quenching effect at elevated concentrations or in solid or aggregated states, which impedes the development of these materials.^[^ ^4^ ^]^ The introduction of the concept of aggregation‐induced emission (AIE) has provided a solution, revealing a class of light‐emitting systems that demonstrate robust emission in solid and aggregated states.^[^ ^5^ ^]^ Since its inception in 2001, AIE systems have been employed across a majority of fields that were formerly dominated by traditional light‐emitting materials.^[^ ^6^, ^7^ ^]^ Concurrently with the research on application, AIE systems have garnered interest from theorists.^[^ ^8^, ^9^ ^]^ Recent years have seen efforts aimed at elucidating the underlying mechanisms and the relationship between chemical structure and properties associated with the AIE phenomenon.^[^ ^10^ ^]^ The restriction of rotation/vibration/motion,^[^ ^11^, ^12^ ^]^ along with the restricted access to conical intersections, represent two famous theoretical frameworks that elucidate the mechanism of AIE in terms of chemical structure and quantum chemistry, respectively.^[^ ^13^ ^]^ Nevertheless, a significant portion of theoretical research is grounded in specific systems. Consequently, there remain ongoing debates regarding the intrinsic characteristics of AIE, attributable to the variability of AIE substructures.^[^ ^14^, ^15^, ^16^ ^]^ In sharp contrast, previous research findings in related disciplines were challenging to synthesize and apply to pertinent studies, leading to a significant amount of redundant research and inefficient use of resources.

At the current stage, our experiments and theoretical calculations on various cases have provided a macroscopic understanding of the mechanism underlying AIE. In condensed phases, the strong emission observed in AIE systems is attributed to their non‐planar geometries, which effectively reduce intermolecular exciton diffusion and inhibit quenching effects. Conversely, in dilute conditions, these non‐planar geometries may be subject to molecular motion that promotes non‐radiative decay. This phenomenon occurs due to non‐adiabatic couplings between excited and ground states, leading to a decrease in emission intensity.^[^ ^17^ ^]^ The discussions regarding the relationship between the chemical structure and the spectral performance as well as other photophysics of AIE‐active luminogen (AIEgens) or other type of photoelectronic materials are dispersed across a considerable number of papers that focus on their specific circumstances or substructures. Scant publications have formed a macroscopic perspective on this topic.^[^ ^18^, ^19^, ^20^ ^]^

The swift advancement of artificial intelligence within the scientific domain has equipped researchers with a robust instrument for both predictive modeling and regression analysis of properties.^[^ ^21^, ^22^, ^23^, ^24^ ^]^ It also helps in understanding the intricacies of chemical systems by employing a more statistical method, which minimizes the bias associated with particular systems.^[^ ^25^ ^]^ Efforts have been undertaken to establish a general criterion for differentiating AIE from other system types. In 2018, Qiu et al. employed a machine‐learning approach to elucidate the characteristics of AIE in triphenylamine derivatives.^[^ ^26^ ^]^ Subsequently, in 2022, Xu et al. introduced a fingerprint‐based methodology aimed at distinguishing AIE from aggregation‐caused quenching (ACQ) systems more broadly.^[^ ^27^ ^]^ These studies have motivated us to conduct a more extensive investigation utilizing a larger dataset. These investigations have prompted us to undertake a more comprehensive analysis employing a larger dataset. The establishment of a database for aggregate science has enabled an initial review of preliminary research concerning AIE from a macroscopic perspective.^[^ ^28^ ^]^ However, currently, the most extensive database within the field of AIE comprises approximately several thousand data entries. Despite the comprehensive efforts to gather and standardize this information, which have nearly encompassed the pertinent literature in the domain, the volume of data remains inadequate when juxtaposed with fields where data science applications have reached a more advanced stage, such as pharmacology. Therefore, in order to explore the field utilizing data science methodologies, there is a significant need to establish a more effective approach for the optimal use of information. This involves eliminating any redundant features that do not have a direct correlation to the subject of interest.

In this study, we introduce a novel interpretable chemical fingerprint specifically designed to encode substructures relevant to photophysics. Every digits of the fingerprint were defined straightforwardly about the AIE effect and optical properties. Initially, we assessed the reliability of this fingerprint in classifying and regressing the photophysical properties of organic materials through various machine learning models. Our findings indicate that fully‐connected neural networks (FCNN) are the most effective models to employ in conjunction with the fingerprint under the appropriate circumstances. The fingerprint demonstrated significant performance and reliability, utilizing a reduced number of features that are more interpretable, which are derived from the chemical structure. Subsequently, we applied techniques of the integrated gradient analysis to analyze the relationship between chemical structure and photophysics, aiming to derive theoretical principles governing AIE and related systems. In the following study on interpretability, we conduct a thorough investigation into the effects of electronic donors, acceptors, and the scale of aromatic conjugation on the macroscopic photophysical properties of aggregate materials, specifically focusing on the AIE effect and emission wavelengths. Our findings yield two key conclusions: 1) The scale of conjugation acts as a double‐edged sword, presenting competing effects that influence both the AIE phenomenon and the characteristics of emission wavelengths; 2) The simultaneous presence of both donor and acceptor components is crucial for a precise evaluation of the AIE phenomenon and its related wavelength characteristics. Ultimately, this research establishes a novel framework for related studies, adopting a more data‐driven approach.

## 2 Results and Discussion

### 2.1 The Design of the Model

It is evident that the chemical structure significantly influences the performance of a given material. If we denote the features of the chemical structure as *s*_1_, *s*_2_, …, *s*_*i*_, and quantify performance as *p*, we can express this relationship with the function:

(1) $$p=F(s_{1},s_{2},\text{…},s_{i})$$

Here, *F* represents the actual relationship between structure and properties. The model need to fit the function between features and the targeted properties.

Given the constraints of the data scale, conventional general‐purpose fingerprints, commonly utilized in computer‐assisted drug discovery and design, often contain excessive redundant information that is not pertinent to photophysics. This redundancy can lead to substantial overfitting and exacerbate data bias, thereby distorting the formulation of theoretical conclusions. So it is essential to develop a novel feature extraction method that minimizes the presence of redundant information. The photophysical properties of organic light‐emitting materials in aggregate or solid‐state forms are influenced by their chemical structure and packing arrangement. Furthermore, the packing arrangement can be deduced from the chemical structure in a reverse manner. The thermodynamic history, which encompasses the overall processes of separation, isolation, and purification–as well as factors such as heating or cooling rates–also plays a crucial role in determining the packing morphology.^[^ ^29^, ^30^, ^31^ ^]^ Researchers recognized that some AIEgens emit light in crystal form while some in single molecule form. This, which have a potent correlation to the molecular structure, is also a great influential factor to the spectral.^[^ ^32^, ^33^, ^34^ ^]^ However, the database was constructed from the data of published papers. As not all the publications encompassed the morphology, and in addition, it could not be uniformly digitized, the overall performance of the model is rather statistical rather than analytical. Nevertheless, considering that the majority of the pristine obtained and reported samples are the most kinetically stable morphology of the corresponding molecule, and the morphology itself is also profoundly influenced by the molecular structure, the model can reflect the structure‐properties relationship of AIE‐related materials that emit lights in either condensed or single molecule forms.

Numerous empirical and intuitive observations have been made regarding the relationship between chemical structure and photophysics:

1) **The conjugation**: In a typical organic system, conjugation is essential for the generation of photoluminescence, and the extent of conjugation impacts the transition energy from the ground state to the first excited state along with its corresponding multiplicity with prolonging the emission wavelength^[^ ^35^ ^]^;
2) **The donors and acceptors**: The unevenness of the distribution of electrons plays a significant role in shaping the spectral characteristics. In a conjugated system, the presence of electron donors and acceptors can also lead to an extension of the emission wavelengths;
3) **The molecular packing**: The most significant characteristic of emitters in solid or aggregate forms is the presence of intermolecular interactions. When these intermolecular interactions enhance the overall dipole separation, the emission wavelength tends to be redshifted. Furthermore, intermolecular interactions may also act as barriers that inhibit molecular motion, thereby exerting a more complex influence on spectral properties depending on specific circumstances.

Traditionally, achieving compounds with very long emission wavelengths necessitates a large conjugated system that incorporates multiple strong electron donors and acceptors. However, focusing on specific systems can make it challenging to identify these effects clearly. In real‐world molecular design, a simple functional group may exhibit several competing effects simultaneously. For example, empirically, strong electron donors and intermolecular dipole interactions enhance the charge transfer effect, thereby extending the emission wavelength. However, a robust donor such as triphenylamine–characterized by its propeller‐like conformation–introduces significant spatial hindrance that inhibits intermolecular dipole interactions. In practical molecular design scenarios, it is crucial to recognize that a single functional group can have multiple competing influences at once. Another example pertains to the TICT (twisted intramolecular charge transfer: A system will experience a twisted equilibrium geometry that necessitates increased spatial accommodation, resulting in prolonged emission following excitation) system; while an increased conjugation scale regularly leads to a longer emission wavelength, solid‐state conditions may introduce more rigid geometries that can hinder the excited state relaxation process, as this process relies on molecular motions. Consequently, such rigidity could counteract the effect of extending emission wavelengths.

However, establishing a quantitative relationship between these factors and optical performance poses challenges. The primary objective of feature selection in this study is to measure the extent of conjugation and quantify the number of electron donors and acceptors, subsequently transforming these values into a descriptor space to serve as input for machine learning models. The transformation of chemical structures into a specifically curated descriptor space, utilizing selected features, constitutes the initial and pivotal phase in the development of a machine learning model. In the context of classification and similarity searches, molecular fingerprints are particularly significant. A conventional fingerprint for a given molecule is represented as a sequential binary string that indicates the presence or absence of particular substructures or patterns within the molecule. Building upon this concept, we introduce a novel hybrid serial fingerprint comprising four components: donors, acceptors, conjugation scale, and conjugation breakers.

**Figure 1:**
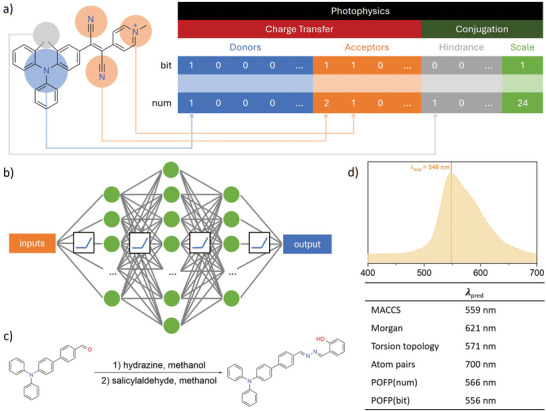
a) Schematic view of generating a photophysics‐oriented fingerprint in this work; b) Schematic view of the neural network model for regression on emission peak wavelengths; c) The synthetic route and d) solid‐state emission spectra with denoted experimental (λ_*exp*_) and FCNN predicted emission peak wavelengths of TPABSA with different fingerprints.

The methodology employed for feature extraction is referred to as photophysics‐oriented fingerprints (POFP). In contrast to general‐purpose fingerprints, POFP exhibits greater interpretability, as its numerical values can be directly associated with photophysical phenomena. Initially, as detailed in the supplementary appendix, we compiled a comprehensive list of electron donors and acceptors that are frequently utilized in the construction of photoluminescent systems. Additionally, we documented typical excited‐state proton transfer (ESIPT) patterns, given their positive impact on emission wavelengths.^[^ ^36^ ^]^ As illustrated in **Figure** **1**a, the extent of conjugation is a significant determinant in photophysical properties. Due to the considerable spatial hindrances posed by bulk groups, we categorized these as “conjugation breakers.” The effects of conjugation breakers can be understood from two perspectives: Bulky groups may introduce non‐planarity, thereby disrupting the conjugation between aromatic rings and effectively reducing the extent of conjugation. Additionally, bulky groups can act as spacers between molecules in a condensed state, diminishing intermolecular dipole interactions or π–π stacking, which often leads to an enhancement in emission intensity. Consequently, we included a list of common “conjugation‐breaker” substructures along with the count of aromatic atoms to represent the overall conjugation scale of a specific chemical structure. Typically, the fingerprint comprises 15 digits representing donors (which include ESIPT patterns, such as Schiff base,^[^ ^37^ ^]^ 2‐hydroxyacetophenone and 3‐hydroxyflavone^[^ ^38^, ^39^ ^]^), 23 digits for acceptors, 7 digits designated for hindrances that act as conjugation disruptors, and a final digit that enumerates the total number of aromatic atoms present. We adopted two distinct methodologies for encoding features derived from chemical structures: the traditional method, designated as POFP(bit), records binary information indicating the presence (1) or absence (0) of a given substructure; the alternative method, termed POFP(num), quantifies the number of specific substructures, replacing the binary digits with their corresponding counts, thereby providing enhanced informational content. Ultimately, we concatenated all digits into a singular series of bits or numbers, resulting in a vector that constitutes the POFP of the respective compounds.

### 2.2 The Performance on Prediction of Photophysics

#### 2.2.1 Emission Maxima Wavelengths

**Figure 2:**
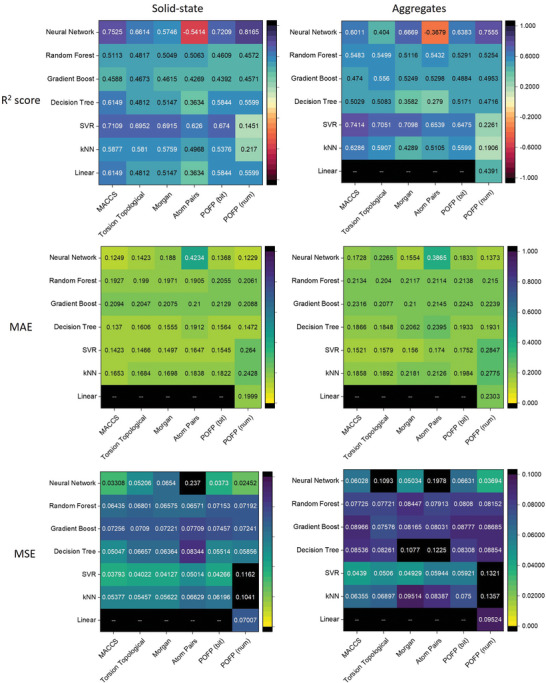
The R square score, mean absolute error (eV) and mean square error of emission wavelength in solid‐state (left) and nanoaggregates (right) of 5‐fold cross‐validation in different combinations of machine‐learning algorithms with different representation methods of chemical structure (Black boxes denoted that the *R* ^2^ < −1, *MAE* > 1 or *MSE* > 0.1).

The designing purpose of POFPs is to predict the photophysics of materials with aggregation‐related effects, particularly AIE. The photophysical property which received the most attention is the emission wavelength. The emission wavelengths in different aggregate forms determine the application of corresponding compounds. Normal algorithms were introduced, including linear regression, support‐vector machine regressor (SVR), k‐nearest neighbors regressor (kNN), decision trees, random forest, and gradient boosting regression. We also constructed a four‐layer fully connected neural network (FCNN), as shown in Figure 1b. The ReLU function activated all layers. For reference, some general‐purpose fingerprints that are already widely used are introduced. General purpose fingerprints, such as molecule access system (MACCS) key,^[^ ^40^ ^]^ Morgan's fingerprint, atom pair fingerprint (AP),^[^ ^41^ ^]^ and topological torsion fingerprint (TT) were developed as universal tools for embedding, comparing, indexing and screening chemical structures from a vast ensemble, especially in the research field of quantified structure‐activity relationship (QSAR) of pharmacy.^[^ ^42^ ^]^ The results were concluded in **Figure** **2**. Most of the tested couples yielded positive R2 scores, implying that the emission wavelengths are related to the chemical structures. It could be found that POFP (num) with the neural network implementation performed best among all tested couples on either R2 score or mean absolute error (MAE).

In the context of pristine solid‐state emission, the use of POFP (num) in conjunction with a fully connected neural network (FCNN) resulted in an *R* ^2^ score of 0.8165 and a MAE of 0.1229 eV. While the comparison may lack significance, the findings are notably close to the errors observed in time‐dependent density functional theory (TD‐DFT) calculations. The FCNN demonstrated promising performance across the majority of tested fingerprints; however, with the exception of POFP (num) paired with FCNN, no other combinations achieved an *R* ^2^ score exceeding 0.8. In comparison to POFP (bit), POFP (num) yielded a superior *R* ^2^ value and a lower MAE, suggesting that the numerical counts of specific donors, acceptors, and other substructures provide more critical information for quantifying properties than merely indicating their presence or absence. The mean squared error (MSE) results corroborated this conclusion, indicating that POFP (num) generally performed well. Although linear models typically exhibit significant deviations when compared to non‐linear models, POFP (num) also demonstrated reasonable error under linear fitting, suggesting that it is the most straightforward embedding method for quantifying photo‐related features among all evaluated fingerprints. To assess the practical applicability of the regression models, we designed and synthesized a compound, TPABSA, and measured its emission spectra in the solid state. TPABSA serves as an illustrative example, being both readily synthesizable and containing intramolecular charge transfer (ICT) and ESIPT substructures. As depicted in Figure 1c, the experimental emission peak wavelength λ_*exp*_ was recorded at 548 nm, while the average predicted wavelength λ_*pred*_ from the POFP (num) FCNN was 566 nm as shown in Figure 1d. The deviations measured in nanometers and electronvolts were 18 nm and 0.072 eV, respectively, indicating a strong predictive capability for screening structures with targeted emissions from a vast array of organic compounds. Notably, in this instance, POFP (bit) achieved even greater accuracy, with a deviation of only 8 nm as shown in Figure 1d.

Actually, there is limited reported work focusing on the prediction of spectral properties in AIE systems.^[^ ^43^, ^44^, ^45^, ^46^ ^]^ Our implementation offers a black‐box approach to directly establish correlations between chemical structures and photophysical properties without requiring additional quantum mechanical calculations or orbital information. Although this method may be subject to dataset bias and reduced robustness, it demonstrates extremely high efficiency in making predictions with instantaneous completion for individual input chemical structures, making it highly suitable for screening large datasets. Furthermore, the efficiency of the predictions does not depend on the size of the molecule, indicating that the model is particularly well‐suited for extensive conjugated systems commonly found in optical materials. Moreover, the input features of POFPs are concrete, which enhances the intuitiveness of interpretability analysis compared to other methods.

#### 2.2.2 Quantum Yields and Fluorescence Lifetime in Solid State

For other photophysical properties, such as fluorescence quantum yields and lifetimes, we also attempted to perform regression analyses using all the aforementioned models and fingerprints. The results are presented in Figures S1 and S2 (Supporting Information). Although the dataset for quantum yields and lifetime data is not as comprehensive as that for emission wavelengths, the findings suggest that POFP(num) utilizing neural networks shows promise, achieving almost the highest accuracy in terms of both MAE and MSE metrics. However, despite the strong performance of POFPs among all tested models, the overall accuracy in predicting quantum yields and lifetimes still exhibits deviations when compared to the recorded data. These deviations may stem from the quality of the data. For the regression of solid‐state quantum yield, it is important to note that the recorded data primarily originate from integral sphere measurements. Such measurements are often characterized by high noise levels, particularly when assessing the quantum yields of weak emitters with very long emission wavelengths. The practical MAE in the regression of quantum yields is ≈0.14, indicating that the model can be applied to systems with a quantum yield of at least 0.42. For lifetime regression, POFP(num) demonstrates a MAE of 1.61 ns and a MSE of 4.64 ns^2^, indicating better performance compared to quantum yield because that most of the tested fluorescence lifetimes fall within the range of 5 to 10 ns. These results suggest that the conventional measurement method for solid‐state fluorescence lifetime has reached a more advanced level of maturity.

#### 2.2.3 Conclusion on Regression Performance

Generally, POFPs utilizing neural networks demonstrated superior performance in most regression scenarios. The influence of structural characteristics on photophysical properties can be elucidated from the aforementioned results. In the majority of tested fingerprints, which solely recorded the presence or absence of corresponding substructures, the linear regression model exhibited significant deviations. Conversely, POFP(num) emerged as the only fingerprint capable of yielding meaningful results when applied with a linear model. This finding suggests that emission maxima wavelengths, quantum yields, and lifetimes are influenced by structural features in a proportional manner rather than through a threshold effect. Consequently, it implies that the number of corresponding moieties is also a critical factor affecting photophysics beyond their mere existence.

In addition, even with the inclusion of POFP(num), the linear model remains suboptimal for regression tasks. In predicting emission wavelengths, the neural network demonstrated exceptional accuracy, significantly outperforming other models. For quantum yields regression, while the neural network maintained its superior performance, decision trees exhibited comparable results. In lifetime regression, SVR secured second place in terms of performance. These findings suggest that all tested photophysical properties do not exhibit a linear correlation with the number of structural features. The underlying relationship is likely to be non‐linear and complex; furthermore, it appears that none of the included features operate independently. It is anticipated that there are interactions among structural features contributing to these outcomes.

### 2.3 Overall Performance on Classification

**Figure 3:**
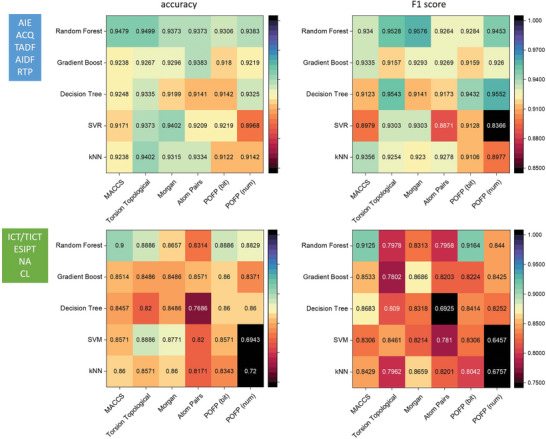
The accuracy and weighted‐average F1 score of the classification model of 5‐fold cross‐validation trained by photophysical features and excited‐state dynamics mechanisms.

While the POFPs were not specifically developed for classification purposes, they nonetheless provided valuable information for the categorization of compounds based on their photophysical characteristics and mechanisms. For the classification benchmarks, we selected several algorithms, including Random Forest, gradient boosting (GB), Decision Trees, support vector machine (SVM), and kNN. Each of these classifiers was utilized in conjunction with the tested fingerprints to conduct classifications, employing a 5‐fold cross‐validation approach. The findings are summarized in **Figure** **3**.

In the context of classifying photophysical behaviors associated with aggregates (such as AIE, ACQ, thermally activated delayed fluorescence (TADF), aggregation induced delayed fluorescence (AIDF), and room temperature phosphorescence (RTP)), the performance of POFP (num) when utilizing random forest and decision tree classifiers demonstrated promising results, albeit not achieving the highest accuracy and F1 scores. It is noteworthy that both the decision tree and its derivative, the random forest classifier, are specifically designed for classification tasks. These classifiers achieved high accuracy rates and F1 scores, ≈0.95, when applied to the MACCS fingerprint, torsion topological fingerprint, and Morgan fingerprint. In terms of accuracy, the MACCS and torsion topological fingerprints outperformed both POFP (bit) and POFP (num). Furthermore, in relation to the F1 score, the performance of the torsion topological and Morgan fingerprints also surpassed that of the POFPs. Conversely, for the classification of mechanisms underlying excited‐state dynamics (including ICT/TICT, ESIPT, neutral aromaticity (NA), and clusterluminescence (CL)), POFP (bit) consistently yielded the best results, achieving the highest F1 score. In our implementation, the TICT and ICT systems were treated as a whole because both are subsets of charge transfer systems characterized by similar structural features. Additionally, the number of TICT and ICT entries in our database is highly unbalanced, which could disrupt the training of classification models. Many TICT systems exhibit AIE activity due to their involvement in a space‐consuming relaxation process and the experience of twisted equilibrium geometries in the excited state. This phenomenon leads to weaker emission, which may be further inhibited in condensed forms as a result of spatial hindrance caused by molecular packing. Since our database has recorded materials with research potential in the field of aggregate science–particularly in AIE–the number of TICT entries is significantly larger than that of other ICT systems.

The observed results are not unexpected, as the fundamental components of the POFP (bit) are comprised of donor, acceptor, and ESIPT moieties. The relationship between other general‐purpose fingerprints and their underlying mechanisms may be more discernible. Notably, the random forest algorithms demonstrated superior performance. The photophysical behavior of aggregates is not solely determined by the presence of specific substructures; it is also significantly affected by the chemical structural rigidity, solubility, and planarity of the molecular geometries. These properties: chemical structural rigidity, solubility, and planarity are extensive in nature and can be influenced by the quantity of particular substructures present. Consequently, the POFP(num) exhibited a marked improvement in performance compared to POFP(bit).

**Figure 4:**
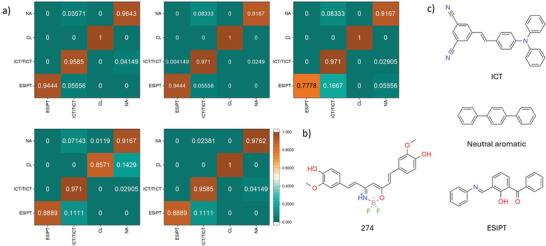
a) The normalized confusion matrices in the classification test on excited‐state dynamics mechanisms of POFP (bit) with the random forest algorithms in 5‐fold cross‐validations, respectively; b) Chemical structure of compound 274 of the database; c) The example molecules and their predicted mechanisms using POFP (bit) with random forest algorithms.

The classification performance regarding the mechanisms was generally inferior to that of the features. As indicated by the confusion matrices derived from 5‐fold cross‐validation, illustrated in **Figure** **4**a, the inadequate discrimination between ESIPT and ICT/TICT mechanisms constrains the overall differentiation capability. This limitation may stem from the fact that a considerable number of ESIPT systems within the database are not exclusively ESIPT but rather hybrid systems that incorporate ICT/TICT substructures. Examples of such systems are presented in Figure 4c. It is evident that the model effectively identifies the key structural characteristics associated with the corresponding excited state dynamics mechanisms.

By the way, it is noteworthy that in Figure 4c, the first compound was classified as an AIEgen due to its triphenylamine (TPA) moiety and stilbene skeleton. In contrast, the second compound, namely p‐terphenyl, is not considered an AIEgen. The p‐terphenyl exhibits a quantum yield of 97% and emits at 338 nm in dilute form.^[^ ^47^ ^]^ Additionally, it demonstrates strong crystalline fluorescence in the blue region; however, this occurs with lower efficiency.^[^ ^48^, ^49^ ^]^ Classifying p‐terphenyl as an AIE system poses challenges due to its high emission efficiency in dilute solutions, which can be attributed to subtle relaxation processes occurring in the excited state. Most AIE systems exhibit significant mobility following excitation. This molecular motion is invariably accompanied by electronic relaxation that drives the excited system toward an equilibrium state characterized by lower energy levels. Consequently, this often results in an enhanced non‐radiative decay rate owing to increased non‐adiabatic couplings between the excited and ground states stemming from a reduced energy gap. However, for p‐terphenyl–despite partial rotational freedom of single bonds between benzene rings–the molecules tend to adopt increasingly co‐planar conformations with higher oscillator strengths and radiative decay rates on the potential energy surface of their excited states. This phenomenon leads to stronger emissions in dilute forms and elucidates why p‐terphenyl does not exhibit typical aggregation‐induced emission (AIE) behavior.

It can be inferred that the classification of features following aggregation (AIE, ACQ, AIDF, TADF, and RTP) is contingent upon the number of specific substructures. This observation arises from the interplay between positive and negative factors influencing emission in solid state as well as in dilute solution, particularly highlighting the competitive dynamics distinguishing AIE from ACQ, implying that POFP(num) performed better. However, materials are often classified based on their excited state dynamic mechanisms–such as ICT/TICT, ESIPT, NA, and clusterluminescence (CL)–with reference to the presence of their representative functional groups. Therefore, for this type of classification, the use of POFP(bit) is more precise.

### 2.4 The Performance in Clustering

The clustering of chemical structures plays a crucial role in the pre‐screening of extensive collections of organic compounds that lack specific labels. Most clustering algorithms aim to enhance the distinctions among data points within a dataset, thereby facilitating their categorization into distinct clusters. General‐purpose fingerprints, which encompass more comprehensive structural information, enable the clustering of compounds based on their nuanced differences. Nevertheless, in practical research contexts, a higher degree of clustering precision does not necessarily equate to improved outcomes. An illustrative example has been presented to demonstrate this distinction.

As illustrated in Figure S3 (Supporting Information), we employed K‐means clustering algorithms to categorize the POFP (num) data points in conjunction with the associated mechanistic information. To enhance visualization, we initially applied t‐SNE (t‐distributed stochastic neighbor embedding). Two ensembles of similar data points are indicated by black and orange circles, both of which consist of six‐membered rings featuring boron bridge substructures. The black box specifically denotes halogen‐derivative boron‐containing systems. Notably, irrespective of the chosen value of K, compound 274 as shown in Figure 4b consistently clustered with its neighboring compounds 1656, 1671, and 1647. An analysis of their chemical structures reveals that all aforementioned molecules incorporate boron bridges, whereas the other compounds represent typical 4,4‐difluoro‐boradiazaindacene (BODIPY) systems, which do not include compound 274. When utilizing MACCS fingerprints for clustering, the algorithms effectively discern the differences, resulting in the exclusion of compound 274 from the larger group across all tests with varying K values, as depicted in Figure S4 (Supporting Information).

Engaging in a debate regarding the superiority of different clustering methodologies is unproductive. The choice of approach should be contingent upon the specific context of the research. General‐purpose fingerprints evaluate features uniformly, irrespective of their relevance to photophysics. In contrast, POFPs, particularly POFP (num), focus exclusively on substructures pertinent to photophysics. Consequently, POFPs demonstrate enhanced performance in classifications related to photophysics when applied to smaller datasets; however, they may overlook minor structural variations.

### 2.5 Explore Chemical Design by the Conditional Variational Autoencoder

**Figure 5:**
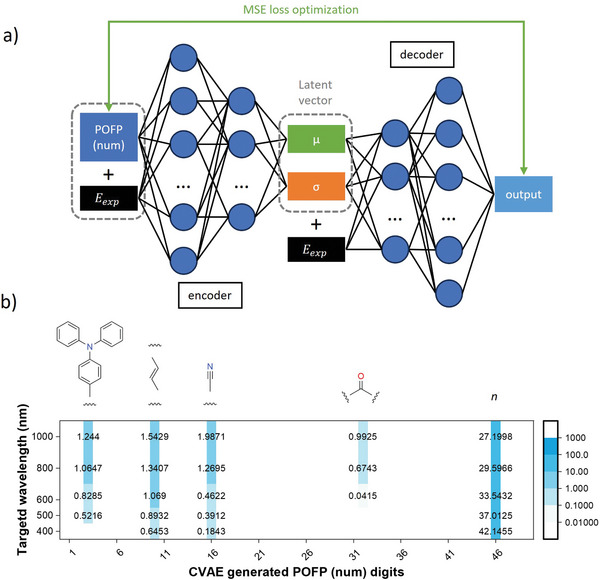
a) The structure of conditional variational autoencoder models in this work (μ and σ denote the mean and standard deviation value of the distribution in the latent space, *E*_*exp*_ stand for the emission energy in solid‐state); b) The CVAE‐generated POFP (num) at point μ = 0 and σ = 0 in the latent space with conditions of different targeted emission wavelengths in solid‐state (n denotes the number of aromatic atoms).

The conditional variational autoencoder (CVAE) is a widely utilized generative model that facilitates the generation of specific content under defined conditions.^[^ ^50^ ^]^ However, one‐directional encoders for chemical structures, such as POFPs, are not suitable for the rational generation of complete chemical structures using CVAE or other generative models. Nonetheless, CVAE can be employed to elucidate the significance of various substructures in relation to photophysics from a macroscopic perspective. In this study, we constructed a basic CVAE, as illustrated in **Figure** **5**a. The input consisted of the POFP (num) of the structures concatenated with the experimental emission energy in the solid state. Following encoding by a two‐layer feedforward neural network (FCNN), the resulting 3D vector was utilized to compute the Kullback–Leibler divergence (KL divergence) in relation to a standard normal distribution, after which the emission energy was concatenated once more. This newly formed 3D vector was subsequently decoded by another two‐layer FCNN to yield an output that matched the dimensions of the original POFP (num). The training process aimed to minimize both the KL divergence and the mean squared error between the output and the original POFP (num). Upon achieving optimal training, the commonalities inherent in the dataset were encapsulated within the parameters of the encoder and decoder, allowing for the specificity of a given structure with a particular emission energy to be adjusted by modifying the latent vector. Furthermore, when inputs of μ = 0, σ = 0, and a designated emission energy value were fed into a pre‐trained decoder, the output was expected to represent the average POFP (num) of chemical structures corresponding to the specified targeted emission wavelengths.

As illustrated in Figure 5b, we selected emission wavelengths of 400, 500, 600, 800, and 1000 nm to calculate average POFP(num)s. Notable trends emerged indicating that the counts of typical donor molecules, such as triphenylamines and carbon–carbon double bonds, as well as common acceptors like cyano and carboxyl groups, increased significantly despite a marked reduction in the total number of aromatic atoms as the targeted wavelengths increased. This finding appears to contradict the chemical intuitions held by many researchers in the field. While the increase in the number of donors and acceptors is logical, the decrease in aromatic atoms presents a more complex scenario. This phenomenon can be attributed to two primary factors: first, the dataset comprises published materials characterized by superior optical properties, necessitating larger conjugation scales to offset the diminished optical characteristics associated with a scarcity of donors and acceptors in systems exhibiting shorter emission wavelengths; second, based on the proposed mechanism of restricted access to a conical intersection and the correlation between fluorescence emission and molecular rigidity, it is posited that rigid systems, typically featuring multiple aromatic rings, are more conducive to maintaining shorter emission wavelengths, particularly in solid‐state environments. This conclusion may challenge the prevailing tendency among some researchers to incorporate as many conjugated rings as possible when designing systems intended for longer emission wavelengths.

### 2.6 Explore Photophysics by the Integrated Gradient Analysis

In order to advance the examination of the complex interplay between chemical structure and macroscopic optical properties, as well as the role of individual substructures, we applied integrated gradient analysis to clarify the models utilized in previous classification and regression research.^[^ ^51^, ^52^ ^]^ It is posited that the pre‐trained neural network operates as a function $\hat{F}\left(s_{1},s_{2},\text{…},s_{i}\right)$ of the features (*s*_*i*_) derived from the fingerprint:

(2) $$\hat{F}\left(s_{1},s_{2},\text{…},s_{i}\right)=f\left(s_{1},s_{2},\text{…},s_{i},w_{1},w_{2},\text{…},w_{k}\right)$$

The variables *w*_*k*_ represent the predicted weights associated with various classes. The gradient of the function $\hat{F}$ on a specific class (*w*_*k*_) with respect to the feature *s*_*i*_ (denoted as $\frac{\partial \hat{F}}{\partial s_{i}}$) serves as an indicator of the sensitivity of feature *i* in relation to the classification outcome. However, the value of $\frac{\partial \hat{F}}{\partial s_{i}}$ at a particular point of *s*_*i*_ only captures the local sensitivity of feature *i* and is susceptible to the issue of gradient saturation. To address this challenge, the integrated gradient model is proposed. This model involves selecting a baseline configuration characterized by features ${\bar{s}}_{1},{\bar{s}}_{2},\text{…},{\bar{s}}_{i}$, which are positioned at a certain distance from the configuration under analysis, represented by features *s*_1_, *s*_2_, …, *s*_*i*_. It is posited that there exist transitional configurations between the baseline and the configuration being analyzed, denoted as *s*_1_(α), *s*_2_(α), …, *s*_*i*_(α). The parameter α signifies the proportion of the distance between the current transitional configuration and the baseline. So the integrated gradient could be expressed as follows after normalization:

(3) $$IG=\left(s_{i}-{\bar{s}}_{i}\right)\int_{0}^{1}\frac{\partial \hat{F}\left(s_{i}\left(\alpha \right)\right)}{\partial s_{i}}d\alpha$$

But in our contribution, when we do not need to compare contributions of different features individually, a simple version of integrated gradient (IG) could be expressed as follow without normalization:

(4) $$IG=\int_{0}^{1}\frac{\partial \hat{F}\left(s_{i}\left(\alpha \right)\right)}{\partial s_{i}}d\alpha$$

It is possible to sample the transitional fingerprints in a linear manner, thus:

(5) $$s_{i}\left(\alpha \right)={\bar{s}}_{i}+\alpha \left(s_{i}-{\bar{s}}_{i}\right)$$

In the implementation, we sampled 100 points at uniform intervals to approximate the value of the integral.

The integrated gradient (IG) method is employed to assess the overall contribution of features *s*_*i*_ to the predicted outcome of a specific structure, utilizing a baseline structure as a reference point. In essence, IG clarifies how the difference between the current structure and the baseline affects the prediction outcome. In practical applications, IG is typically approximated by summing the gradients derived from discrete fingerprints. The formulation of the baseline structure has a substantial influence on the IG. Consequently, modifications to the baseline may lead to the calculated IG reflecting different aspects of the pre‐trained model.

Our initial findings suggest that the incorporation of spatial hindrance may mitigate intermolecular interactions, thereby improving the emission characteristics of the system in solid‐state and aggregate forms. Furthermore, the integration of flexible substructures at excited state, such as TICT moieties or double bonds, facilitates molecular motion in the excited state, which may lead to a reduction in emission when in dilute solutions. We chose the most famous AIE system – tetraphenylethylene (TPE) as the analyzing structure and the blank fingerprint (all *s*_*i*_ equals to 0) as the baseline. By feeding the fingerprint to the pre‐trained classification neural network, the predicted class of TPE is AIE. The TPE has two active fingerprint digits in POFP(num): the 10th (equal to 1, representing that TPE contains one carbon–carbon double bond) and the last one (equal to 24, representing that TPE has total of 24 aromatic atoms). In this section, Equation (3) was utilized to generate a normalized value for the purpose of comparison. The IG of the former one and the latter one were calculated to be 1.27294 and –0.27294, respectively. The result may also anti‐intuition that the scale of aromatic conjugation is not positive for being a AIEgen. However, upon reevaluating the situation, it becomes evident that extensive conjugation does not inherently contribute positively to the system's ability to exhibit AIE. Conversely, in the absence of flexible structural components, larger aromatic conjugated systems may considerably amplify molecular interactions within the aggregated state. This enhancement can, in turn, diminish the fluorescence intensity of the aggregated state, thereby adversely affecting the likelihood of the entire molecule functioning as an AIE system. In the context of an AIE system, it is essential for its flexible substructures to mitigate the adverse effects associated with conjugation. Larger aromatic conjugation systems necessitate the incorporation of more flexible substructures, such as the ethylene moiety, to effectively address these challenges.

As illustrated in Figure S5b (Supporting Information), salicylaldehyde azines (SA) exemplify a typical AIE system characterized by excited‐state intramolecular proton transfer (ESIPT) patterns.^[^ ^53^ ^]^ The calculated weights for various structural components were 0.95342 for the phenol‐imide ESIPT pattern (*s*_13_), 0.95342 for the carbon–nitrogen double bond (*s*_17_), and ‐0.79828 for aromatic atoms. Previous studies have indicated that ESIPT possesses significant potential for AIE, as evidenced by numerous preliminary investigations.^[^ ^54^, ^55^ ^]^ Conversely, the presence of aromatic systems has been shown to exert a negative influence on the AIE effect. A similar observation was made in the investigation of the IGs of TPABSA, where the weights were determined to be 0.74732 for TPA, 1.22421 for the phenol‐imide ESIPT pattern, and 3.92544 for the carbon–nitrogen double bond, with a negative weight of –4.89696 for aromatic atoms. In the case of tetraphenylpyrazine (TPP), the weights were recorded as 0.37946 for aromatic nitrogen (*s*_30_) and 0.62054 for the number of aromatic atoms.^[^ ^56^, ^57^ ^]^ Notably, TPP represents the sole example in our study that exhibited a positive contribution from aromatic atoms as shown in Figure S5d (Supporting Information). Figure S5e (Supporting Information) illustrates the relationship between the weights of aromatic nitrogen and the total number of aromatic atoms in a system that simulates TPP, with variations in the total count of aromatic atoms. The data suggest that the contribution of aromatic atoms to the AIE effect is not constantly positive or negative. Specifically, when the number of aromatic atoms ranges from 3 to 14, a negative correlation with the conjugation scale is observed. Conversely, when the number of aromatic atoms exceeds 14, a positive correlation emerges, indicating the system's classification as an AIE system. This finding suggests that, in analogous systems, while the enhancement of rigidity and intermolecular interactions may negatively impact the AIE phenomenon, there exists a competing effect. As the number of aromatic atoms increases, the substitution around the central pyrazine ring becomes increasingly congested, leading to greater steric hindrance, which inhibits intermolecular interactions and positively influences the AIE effect. In the case of TPP, the latter effect is more pronounced, resulting in a positive value of IG relative to the number of aromatic atoms.

**Figure 6:**
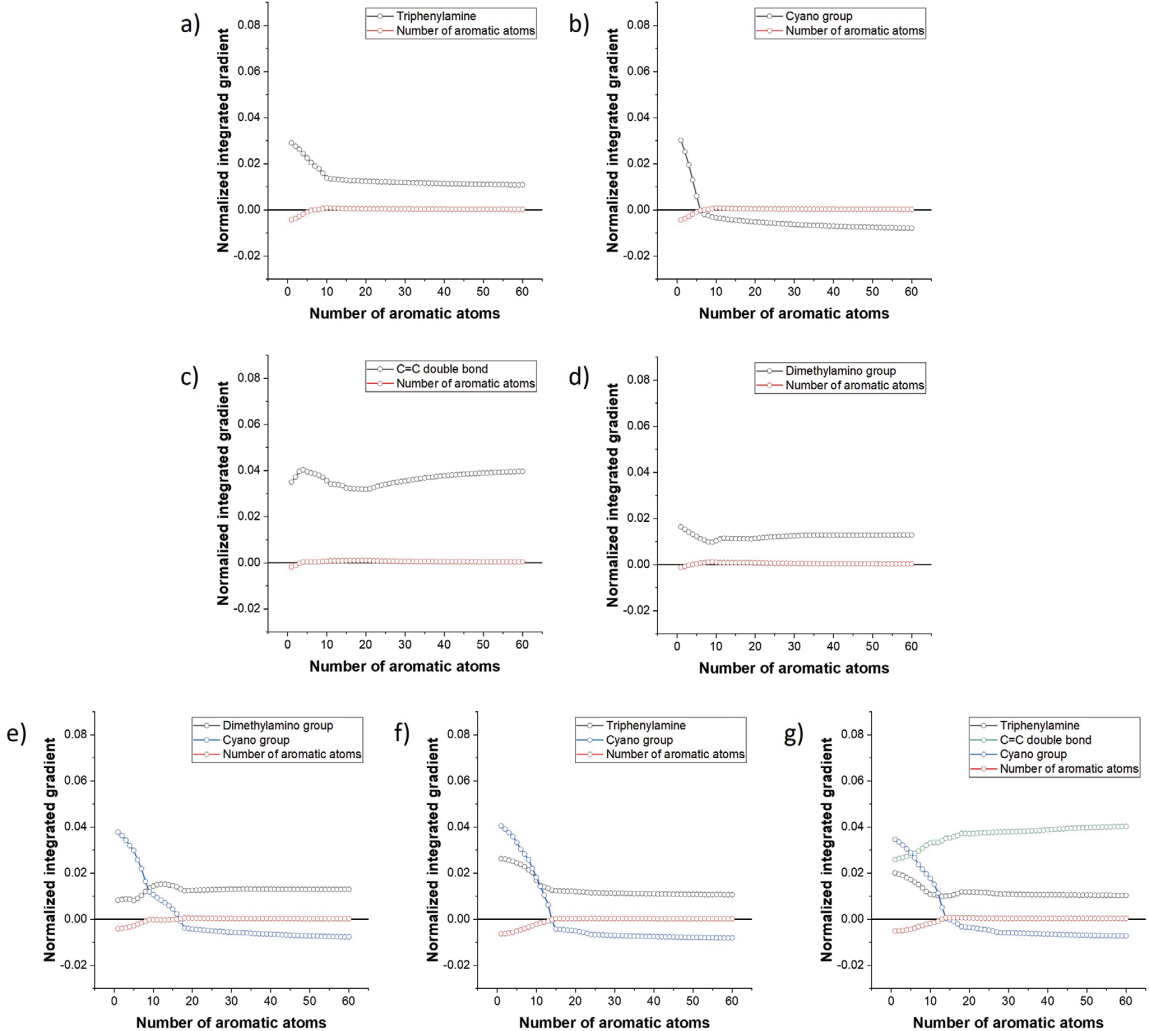
The normalized integrated gradients on identifying AIE systems were analyzed in relation to the increment in the number of aromatic atoms across four distinct fingerprints, each characterized by the presence of a single additional active digit. These fingerprints correspond to: a) triphenylamine (*s*_3_), b) cyano group (*s*_16_), c) carbon–carbon double bond (*s*_10_), and d) dimethylamino group (*s*_1_), respectively. The normalized integrated gradients upon increasing number of aromatic atoms with co‐existence of e) dimethylamino group and cyano group, f) triphenylamine and cyano group, as well as g) triphenylamine, carbon‐carbon double bond and cyano group. All above plots were calculated when the baseline fingerprint was characterized by *s*_10_ = 0.5 and *s*_46_ = 6.

The subsequent sections will focus on the trends in the variation of the IG of specific features. To facilitate the calculations and ensure the plots maintain a smooth appearance, Equation (4) has been employed. In an optimal scenario, the baseline structure should yield anticipated outcomes that exhibit comparable weights across various classes. For this analysis, we have selected a proposed fingerprint characterized by *s*_10_ = 0.5 and *s*_46_ = 6, which demonstrates normalized prediction weights of 0.3102 for ACQ, 0.3694 for AIE, and 0.3204 for the remaining categories (RTP, AIDF, or TADF). In order to achieve a more comprehensive understanding of the structure‐property relationships, we endeavored to create a series of hypothetical fingerprints characterized by a continuous variable representing the number of aromatic atoms (*s*_46_), alongside a singular active digit. Four distinct fingerprints were identified, each indicating the presence of specific substructures: triphenylamine (*s*_3_), cyano group (*s*_16_), carbon–carbon double bond (*s*_10_), and dimethylamino group (*s*_1_). The findings were subsequently presented in the **Figure** **6**. In a system defined by a single triphenylamine, dimethylamino or cyano group, the increase in the number of aromatic atoms generally diminishes the relevance of these specific substructures in the characterization of the fingerprint associated with an aggregation‐induced emission (AIE) system. The observed phenomenon regarding the TPA can be explained by the notion that as the extent of conjugation increases, the spatial hindrance and flexibility of a single TPA unit become inadequate to offset the intensified intermolecular interactions. In the case of the cyano group, which lacks sufficient flexibility and spatial hindrance, its detrimental impact on the AIE classification is clearly evident in the graphical representation, particularly when the structural parameters possess chemical rationality (*s*_46_ > 6). Nevertheless, as illustrated in Figure 6e,f, the presence of donor co‐existence results in the IGs of the cyano group becoming negative when associated with a greater number of aromatic atoms, thereby facilitating chemical rationality. The cyano group, when considered independently, does not impart flexibility or steric hindrance; instead, it serves to enhance charge separation and dipole interactions, which adversely affects the AIE effect. Conversely, in the framework of donor molecules that demonstrate the potential for the TICT behavior, the incorporation of a cyano group can promote the TICT effect, thereby exerting a beneficial influence on the AIE phenomenon. In contrast, carbon–carbon double bonds demonstrate a distinct trend, wherein their importance in predicting AIEgen behavior increases following an initial decline. Carbon–carbon double bonds can unequivocally offer optical flexibility in the excited state as a result of photo‐induced E/Z isomerization.^[^ ^57^, ^58^ ^]^ However, when the number of aromatic atoms is equal to or less than 12, the likelihood of the system being non‐planar is not guaranteed. Stilbene serves as a pertinent example of this hypothesis. In the case of planar geometry, a double bond can function as a conjugation extender, potentially augmenting intermolecular interactions. The interplay between molecular flexibility and intermolecular interactions accounts for the initial decrease observed in the plot. When the number of aromatic atoms exceeds twelve, there is a heightened likelihood of the system adopting a non‐planar conformation, which considerably diminishes intermolecular interactions. Consequently, the significance of carbon–carbon double bonds increases due to their optical flexibility. However, the introduction of a substructure exhibiting inherent steric hindrance, as illustrated in Figure 6g, results in a consistent increase in the significance of carbon–carbon double bonds as the extent of conjugation is expanded.

**Figure 7:**
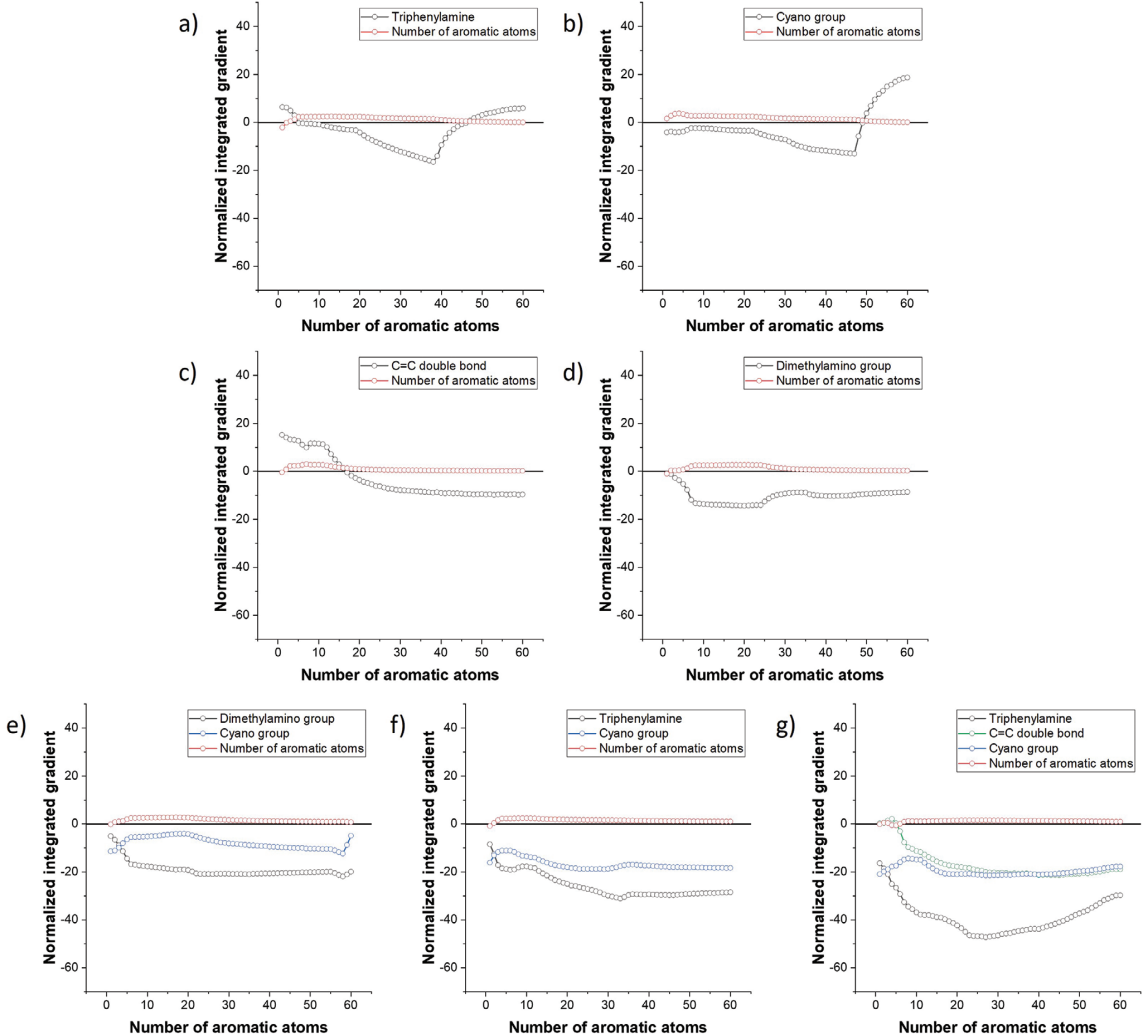
The normalized integrated gradients on regression of solid state emission transition energy were analyzed in relation to the increment in the number of aromatic atoms across four distinct fingerprints, each characterized by the presence of a single additional active digit. These fingerprints correspond to: a) triphenylamine (*s*_3_), b) cyano group (*s*_16_), c) carbon‐carbon double bond (*s*_10_), and d) dimethylamino group (*s*_1_), respectively. The normalized integrated gradients upon increasing number of aromatic atoms with co‐existence of e) dimethylamino group and cyano group, f) triphenylamine and cyano group, as well as g) triphenylamine, carbon–carbon double bond and cyano group. All above plots were calculated when the baseline fingerprint was characterized by *s*_10_ = 0.5 and *s*_46_ = 6.

An integrated gradient analysis was conducted on the regression model designed to predict solid‐state emission wavelengths. There exist two opposing influences exerted by donors and acceptors. On one hand, the enhanced charge separation is expected to extend the emission duration. Conversely, certain large and flexible donors and acceptors exhibit spatial hindrances that can reduce dipole interactions, thereby leading to a decrease in emission wavelengths in the solid state. This phenomenon is illustrated in Figure S5c,d (Supporting Information), where it is evident that some donors and structures characterized by spatial hindrances still demonstrate positive IGs when the number of aromatic atoms in the baseline structure is varied. This observation suggests their potential to increase energy gaps. **Figure** **7** presents results that are similar yet more comprehensive, utilizing *s*_10_ = 0.5 and *s*_46_ = 6 as the baseline for fingerprint analysis. The figure indicates that the presence of the TPA, cyano group, and dimethylamino group contributes to a reduction in transition energy within a reasonable range of aromatic atom counts, resulting in a red shift in wavelengths. This observation aligns with expectations due to the pronounced charge separation effect. Conversely, TPA introduces increased spatial hindrance, which subsequently raises transition energy in the solid state by disrupting intermolecular dipole interactions. Notably, the cyano group exhibits a sophisticated behavior, as it also influences transition energy when the number of aromatic atoms exceeds 50. The influence of carbon–carbon double bonds on transition energy exhibited a range from positive to negative as the number of aromatic atoms surpassed ≈15. The observed negative effect can be attributed to the role of the double bond as a conjugation extender. The findings presented in Figure 7e,g, which illustrate the simultaneous presence of multiple groups, suggest that all examined groups have the capacity to extend the emission wavelengths, a conclusion that is intuitively evident.

Above all, some compelling conclusions can be derived from the aforementioned investigation:

1) First, the conjugation scale is a double‐edged sword, impacting both the AIE phenomenon and the wavelength characteristics. an increased conjugation scale tends to enhance the rigidity of the molecular system, thereby reducing molecular mobility in an excited state, which may lead to a decrease in emission wavelength and adversely affect the AIE characteristics. However, an increased scale of conjugation would lead to a reduction in the energy gap, which is advantageous for extending the wavelengths of emission. There exists a competitive dynamic between these two effects.
2) Second, the presence of both the donor and acceptor is essential for accurately assessing the influence of the AIE phenomenon and its associated wavelength. In systems characterized by a single donor or acceptor, an increase in the extent of conjugation frequently reduces the significance of the donor or acceptor in classifying the system as an AIEgen. The presence of only one donor or one acceptor does not consistently correlate with an extension of the emission wavelength. Conversely, in systems that feature both a donor and an acceptor, the contributions of each to the AIE behavior are enhanced concurrently. When assessing the energy of emission transitions, the simultaneous presence of donors and acceptors may render both components adequate for extending the emission wavelength.

## 3 Conclusion

In this study, we performed an extensive analysis of the molecular design of AIE systems utilizing machine learning techniques that prioritize interpretability. This approach aims to elucidate the relationship between chemical structures and photophysical properties, thereby enhancing our understanding of the AIE phenomenon and the characteristics of AIE materials. Ultimately, this research seeks to establish a more robust design strategy for these materials. Unlike previous theoretical studies, this research has developed a feature extraction framework specifically designed for AIE and related systems. An interpretable fingerprints as the core of the framework, referred to as POFPs, was designed, which offer two primary advantages when compared to previously established methods: 1) POFPs encompass decades of parameters, whereas traditional models typically involve hundreds of features. Given that the total number of data entries is relatively limited, the model may struggle to effectively process such a large number of parameters, thereby increasing the likelihood of overfitting; 2) Traditional fingerprints are designed for general purposes and have been demonstrated to be promising in the development of medicinal compounds. However, the design principles governing medicinal chemicals differ significantly from those applicable to light‐emitting materials. In terms of photophysics, traditional fingerprints contain numerous redundant features that can exacerbate overfitting and constrain the robustness of the trained model. Moreover, the POFPs characterized by photophysics‐oriented parameters are more appropriate for analyzing interpretability. This framework exhibits significant performance, achieving high accuracy and precision in both the classification of materials and the regression of emission transition energy, while also ensuring interpretability. Furthermore, the study presents a comprehensive methodology for conducting mechanistic investigations of AIE, adopting a broad perspective rather than focusing solely on case studies. Then the methodology employed the conditional variational autoencoder and integrated gradient analysis for enhanced understanding. The results discussed are largely intuitive, highlighting the adverse impact of non‐planar and flexible geometries on the AIE effect. This suggests a significant practical application in research concerning the structure‐properties relationship. In the following study, the impact of donor and acceptor groups, along with the scale of aromatic conjugation, on the AIE effect and the emission wavelength in the solid state were be summarized.

To the best of our knowledge, we have conducted the inaugural comprehensive investigation into the structure‐property relationships, utilizing a novel fingerprint specifically developed for the AIE effect and photophysics, with an emphasis on interpretability. This research introduces a new paradigm for examining the correlation between chemical structure and the photophysics of AIE systems, drawing upon the cumulative advancements in the field to date. This approach may yield more robust and generalizable conclusions compared to analyses that concentrate solely on individual compounds or systems.

## 4 Experimental Section

### Data Collection and Clean

All photophysical data utilized in this study were sourced from ASBase (http://www.asbase.cn).^[^ ^28^ ^]^ In the practice, certain data entries that contain invalid SMILES or transition metal ions which were not included in the POFPs were excluded, as the implementation of POFPs relies on the conversion process from the SMILES representations of organic systems featuring main group metal elements. The distributions of the emission transition energies for the training dataset are presented in Figures S6 and S7 (Supporting Information). For the regression analysis, the dataset comprised 708 entries with documented solid‐state emission wavelengths ranging from 337 to 1100 nm. Additionally, the emission wavelengths observed in nanoaggregates included 637 entries, spanning from 323 to 1060 nm. As the dependent variable for the training process, the emission wavelengths were initially converted to energy values in electronvolts (eV) using the Equation:

(6) E≈1240/λ

The symbol λ represents the emission wavelengths measured in nanometers (nm). In the context of the classification experiment, a total of 1,092 records of compounds exhibiting documented photophysical characteristics were analyzed. The recorded characteristics encompassed phenomena such as simple AIE, ACQ, TADF, RTP, and AIDF. Additionally, there were 360 entries that included documented mechanisms of excited‐state dynamics, which featured processes such as simple and twisted intramolecular charge transfer (ICT/TICT),^[^ ^59^ ^]^ excited‐state intramolecular proton transfer (ESIPT), neutral aromaticity (NA), and cluster luminescence (CL).^[^ ^60^, ^61^, ^62^ ^]^ All entries were selected without the application of specific criteria.

Small data sets can amplify the influence of abnormal instances due to their higher weights in the fitting process. One potential issue is that all data entries are sourced from ASBase, a database of published materials, which indicates that all chemical structures have been pre‐screened by researchers in the relevant field. Consequently, the model may inadvertently exacerbate biases present in the data set, leading to potential over‐interpretation of results. However, this represents a bottleneck that all projects involving AI and machine learning must confront. The most appropriate solution at the current stage is to expand the scale of the dataset and ensure a balanced representation of data entries with diverse features.

### Codes

To quantify the number of donor and acceptor species, the RDKit software version 2023.09.6 was employed. The machine learning algorithms were implemented on Python 3.9 with packages including Pytorch and Scikit‐learn ver. 1.5.1.^[^ ^63^, ^64^ ^]^ The source code for the generation of the fingerprint in Python is available at https://github.com/JGong‐CatenaryGong/POFP.

### Definition of POFP

All features were delineated using SMARTS patterns and included in the **Table** **1**. The identification and enumeration of corresponding features were executed through substructure matching algorithms provided by the RDKit package.

**Table 1:** The definition and SMART patterns of features in POFP.

No	Category	Name	SMART Pattern
1	donors and ESIPT patterns	dialkylamino	[c][N]([C])[C]
2		diphenylamine	[c][N]([c])[C]
3		triphenylamine	[c][N]([c])[c]
4		methoxy	[c][O][C]
5		diphenyloxy	[c][O][c]
6		phenothiazine	[c][S][c][c][N][c]
7		phenoxazine	[c][O][c][c][N][c]
8		thiophene	[s]‐1[c][c][c][c]‐1
9		furan	[o]‐1‐[c][c][c][c]‐1
10		carbon–carbon double bond	[C]=[C]
11		1,2‐dihyroxy aromatic ring	[O][c][c][O]
12		naphthalene	[c]‐1[c][c][c]([c][c]‐1)[c][c][c][c]‐2
13		ESIPT: enol‐imide	[N]=[C;H][c][c][O;H]
14		ESIPT: enol‐keto	[O]=[C;H][c][c][O;H]
15		ESIPT: 5m ring	[c][C](=[O])[C](=[C])[O;H]
16	acceptors	cyano	[C]#[N]
17		carbon–nitrogen double bond	[C]=[N]
18		aromatic positive nitrogen	[n+]
19		aromatic positive oxygen	[o+]
20		boron as acceptor	[c][B]
21		BF2 bridge	[O,N][B]([F])[F]
22		benzothiazole	[C][c](s)[n]
23		benzoxazole	[C][c](o)[n]
24		benzoimidazole	[C][c](n)[n]
25		aromatic N–S–N	[n][s][n]
26		N=S=N	[c][N]=[S]=[N][c]
27		aromatic N–Se–N	[n][se][n]
28		TPA‐BMO	[C]=[C]([N])[C](=[O])[O]
29		bisimide	[c][C](=[O])[N][C](=[O])[c]
30		aromatic nitrogen	[n]
31		indanone	[C]=[C]([C](=[O])[c])[C](=[O])[c]
32		carboxy	[C]=[O]
33		carbon–carbon triple bond	[c][C]#[C]
34		4‐cyano‐indanone	[N]#[C][C]([C]#[N])=[C]([c])[C](=[C])[C](c)=[C]([C]#[N])[C]#[N]
35		pyrazine‐NBD	[c][c][n][c][c]([c])[n,c][s][n,c][c]([c])[c][n][c][c]
36		fluoride	[C,c][F]
37		flavone	[O]=[C,c][c][c]([c])[S,s,O,o]
38		heavy metal atoms	[Au,Bi,Ir,Ru,Eu]
39	conjugation breakers	spatial conflict of aromatic hydrogen	[C][c][c]([c,C])[c]
40		BINAP core	[c][c]([c])[c]([c])[c]([c])[c]([c])[c]
41		saturated aromatic bridge	[c][C]([c])‐[C]
42		BF4 cation	[F][B‐]([F])([F])[F]
43		PF6 cation	[F][P‐]([F])([F])([F])([F])[F]
44		t‐butyl	[C]([C])([C])[C]
45		continual benzene rings	[c]([c])[c]([c])[c]([c])[c]([c])[c]([c])[c]([c])[c]([c])[c]([c])

### Materials and Instruments

All chemicals were purchased from J&K Chemicals unless otherwise specified and were used as received. H and C13 NMR spectra were measured on a Bruker ARX 400 MHz spectrometer and a Bruker ARX 500 MHz spectrometer. The photoluminescence (PL) spectra were recorded on a HITACHI F4600 fluorescence spectrometer.

### Synthesis—The Synthesis of TPABCHO

TPABCHO, with IUPAC name as 4'‐(Diphenylamino)‐4‐biphenylcarbaldehyde, was synthesized by the method from the literature.^[^ ^65^ ^]^

#### Synthesis—The Synthesis of TPABSA

Aqueous hydrazine of 4.2 mmol was dissolved in 40 mL of methanol. 4.0 mmol TPABCHO was dispersed in 10 mL of methanol in another flask. Then, the TPABCHO‐methanol mixture was added to the hydrazine‐methanol solution dropwise in 20 min. Keep the system under reflux for 2 h. Then 4.0 mmol salicylaldehyde was added to the system. Yellow precipitation formed instantly. Filter the solid and wash it with 10 mL methanol three times and 10 mL de‐ionized water three times. The resulted yellow crystalline powder was the TPABSA.

^1^H‐NMR (400 MHz, DMSO‐d6) δ 11.32 (s, 1H), 8.98 (s, 1H), 8.84 (s, 1H), 7.97 ‐ 7.92 (m, 2H), 7.84 ‐ 7.79 (m, 2H), 7.76 ‐ 7.65 (m, 4H), 7.39 ‐ 7.32 (m, 4H), 7.13 ‐ 6.98 (m, 10H).

^13^C‐NMR (400 MHz, DMSO‐d6) δ 163.53, 163.26, 162.53, 159.23, 159.15, 147.92, 147.35, 142.95, 133.73, 133.52, 133.02, 132.48, 131.72, 131.30, 130.16, 129.62, 128.26, 126.94, 124.95, 124.03, 123.25, 120.07, 120.03, 118.69, 117.02, 116.96, 40.61, 40.41, 40.20, 39.99, 39.78, 39.57, 39.36.

## Conflict of Interest

The authors declare no conflict of interest.

## Supporting information



Supporting Information

## Data Availability

The data that support the findings of this study are openly available in [ASBase] at [https://www.asbase.cn], reference number [24].

## References

[1] Principles of Fluorescence Spectroscopy, (Ed.: J. R. Lakowicz ), Springer US, Boston, MA 2006, pp. 1–26.

[2] Principles of Fluorescence Spectroscopy, (Ed.: J. R. Lakowicz ), Springer US, Boston, MA 2006, pp. 63–95.

[3] V.‐N. Nguyen , A. Kumar , M. H. Lee , J. Yoon , Coord. Chem. Rev. 2020, 425, 213545.

[4] J. R. Albani , in Structure and Dynamics of Macromolecules: Absorption and Fluorescence Studies, (Ed.: J. R. Albani ), Elsevier Science, Amsterdam 2004, pp. 141–192.

[5] J. Luo , Z. Xie , J. W. Y. Lam , L. Cheng , H. Chen , C. Qiu , H. S. Kwok , X. Zhan , Y. Liu , D. Zhu , B. Z. Tang , Chem. Commun. 2001, 37, 1740. 10.1039/b105159h 12240292

[6] X. Cai , B. Liu , Angew. Chem., Int. Ed. 2020, 59, 9868. 10.1002/anie.202000845 32128951

[7] R. Hu , X. Yang , A. Qin , B. Z. Tang , Mater. Chem. Front. 2021, 5, 4073.

[8] Y. Hong , J. W. Y. Lam , B. Z. Tang , Chem. Commun. 2009, 45, 4332. 10.1039/b904665h 19597589

[9] Q. Peng , Z. Shuai , Aggregate 2021, 2, e91.

[10] H. Zhang , Z. Zhao , A. T. Turley , L. Wang , P. R. McGonigal , Y. Tu , Y. Li , Z. Wang , R. T. K. Kwok , J. W. Y. Lam , B. Z. Tang , Adv. Mater. 2020, 32, 2001457. 10.1002/adma.202001457 32734656

[11] T. Virgili , A. Forni , E. Cariati , D. Pasini , C. Botta , J. Phys. Chem. C 2013, 117, 27161. 10.1039/c6cp02988d 27334668

[12] P. Zhou , P. Li , Y. Zhao , K. Han , J. Phys. Chem. Lett. 2019, 10, 6929. 31647671 10.1021/acs.jpclett.9b02922

[13] X.‐L. Peng , S. Ruiz‐Barragan , Z.‐S. Li , Q.‐S. Li , L. Blancafort , J. Mater. Chem. C 2016, 4, 2802.

[14] J. Guan , C. Shen , J. Peng , J. Zheng , J. Phys. Chem. Lett. 2021, 12, 4218. 33900751 10.1021/acs.jpclett.0c03861

[15] N. B. Shustova , T.‐C. Ong , A. F. Cozzolino , V. K. Michaelis , R. G. Griffin , M. Dincă , J. Am. Chem. Soc. 2012, 134, 15061. 22889020 10.1021/ja306042w PMC3448963

[16] D. Presti , L. Wilbraham , C. Targa , F. Labat , A. Pedone , M. C. Menziani , I. Ciofini , C. Adamo , J. Phys. Chem. C 2017, 121, 5747.

[17] J. Yang , M. Fang , Z. Li , Aggregate 2020, 1, 6.

[18] S. Li , L. Zhan , N. Yao , X. Xia , Z. Chen , W. Yang , C. He , L. Zuo , M. Shi , H. Zhu , X. Lu , F. Zhang , H. Chen , Nat. Commun. 2021, 12, 4627. 34330911 10.1038/s41467-021-24937-5 PMC8324909

[19] X.‐M. Sun , J. Liu , Z.‐H. Li , Y.‐P. Fu , T.‐T. Huang , Z.‐D. Tang , B. Shi , H. Yao , T.‐B. Wei , Q. Lin , Chin. Chem. Lett. 2023, 34, 107792.

[20] Y. Pei , L. Liu , X. Cao , J. Zhou , C. Liu , Front. Mater. 2024, 11.

[21] M. C. Melo , J. R. M. A. Maasch , C. de la Fuente Nunez , Machine Learning for Drug Discovery, American Chemical Society, Washington, DC, USA 2022.

[22] J. P. Janet , H. J. Kulik , Machine Learning in Chemistry, ACS In Focus. American Chemical Society, American Chemical Society, Washington, DC 2020.

[23] K. T. Butler , F. Oviedo , P. Canepa , Machine Learning in Materials Science, ACS In Focus. American Chemical Society, American Chemical Society, Washington, DC 2021.

[24] C&EN Global Enterprise 2018, 96, 16.

[25] S. Dhoble , T.‐H. Wu , Kenry , Angew. Chem., Int. Ed. 2024, 63, e202318380. 10.1002/anie.202318380 38687554

[26] J. Qiu , K. Wang , Z. Lian , X. Yang , W. Huang , A. Qin , Q. Wang , J. Tian , B. Tang , S. Zhang , Chem. Commun. 2018, 54, 7955. 10.1039/c8cc02850h 29956696

[27] S. Xu , X. Liu , P. Cai , J. Li , X. Wang , B. Liu , Adv. Sci. 2022, 9, 2101074. 10.1002/advs.202101074 PMC8760175 34821473

[28] J. Gong , W. Gong , B. Wu , H. Wang , W. He , Z. Dai , Y. Li , Y. Liu , Z. Wang , X. Tuo , J. W. Lam , Z. Qiu , Z. Zhao , B. Z. Tang , Aggregate 2023, 4, e263.

[29] S. Anitha , D. J. Thiruvadigal , T. S. Natarajan , Mater. Lett. 2011, 65, 167.

[30] G. Madras , B. J. McCoy , Acta Mater. 2003, 51, 2031.

[31] S. Ding , X. Huang , Q. Yin , Y. Dong , Y. Bai , T. Wang , H. Hao , Chem. Eng. Sci. 2021, 233, 116390.

[32] H. Tong , Y. Hong , Y. Dong , M. Häußler , J. W. Y. Lam , Z. Li , Z. Guo , Z. Guo , B. Z. Tang , Chem. Commun. 2006, 42, 3705. 10.1039/b608425g 17047818

[33] S. Sasaki , S. Suzuki , W. M. C. Sameera , K. Igawa , K. Morokuma , G.‐i. Konishi , J. Am. Chem. Soc. 2016, 138, 8194. 27300152 10.1021/jacs.6b03749

[34] H. S. Sasmal , A. Kumar Mahato , P. Majumder , R. Banerjee , J. Am. Chem. Soc. 2022, 144, 11482. 35754375 10.1021/jacs.2c02301

[35] Y. Yamaguchi , Y. Matsubara , T. Ochi , T. Wakamiya , Z.‐i. Yoshida , J. Am. Chem. Soc. 2008, 130, 13867. 18816053 10.1021/ja8040493

[36] P. Zhou , K. Han , Aggregate 2022, 3, e160.

[37] Y. Zhao , X. Cui , M. Cui , C. Zhang , Q. Meng , J. Lumin. 2022, 248, 118951.

[38] Y. Hu , H. Luo , L. Zhao , X. Guo , S. Wang , R. Hu , G. Yang , Org. Biomol. Chem. 2024, 22, 1850. 38345427 10.1039/d3ob01953e

[39] B. Liu , J. Wang , G. Zhang , R. Bai , Y. Pang , ACS Appl. Mater. Interfaces 2014, 6, 4402. 24571859 10.1021/am500102s PMC3985879

[40] J. L. Durant , B. A. Leland , D. R. Henry , J. G. Nourse , J. Chem. Inf. Comput. Sci. 2002, 42, 1273. 12444722 10.1021/ci010132r

[41] R. E. Carhart , D. H. Smith , R. Venkataraghavan , J. Chem. Inf. Comput. Sci. 1985, 25, 64.

[42] M. A. Johnson , G. M. Maggiora , Concepts and Applications of Molecular Similarity, Wiley, New Jersey 1990.

[43] J. Westermayr , P. Marquetand , Chem. Rev. 2021, 121, 9873. 33211478 10.1021/acs.chemrev.0c00749 PMC8391943

[44] H. Kawai , Y. O. Nakagawa , Mach. Learn.: Sci. Technol. 2020, 1, 045027.

[45] C.‐W. Ju , H. Bai , B. Li , R. Liu , J. Chem. Inf. Model. 2021, 61, 1053. 33620207 10.1021/acs.jcim.0c01203

[46] M. Sumita , X. Yang , S. Ishihara , R. Tamura , K. Tsuda , ACS Cent. Sci. 2018, 4, 1126. 30276245 10.1021/acscentsci.8b00213 PMC6161049

[47] I. B. Berlman , in Handbook of Fluorescence Spectra of Aromatic Molecules, 2nd ed., (Ed.: I. B. Berlman ), Academic Press, Cambridge, MA 1971, pp. 67–95.

[48] C. Adachi , T. Tsutsui , S. Saito , Appl. Phys. Lett. 1990, 56, 799.

[49] Q. Ai , P. Chen , Y. Feng , Y. Xu , J. Appl. Crystallogr. 2017, 50, 278.

[50] J. Lim , S. Ryu , J. W. Kim , W. Y. Kim , J. Cheminformatics 2018, 10, 31. 10.1186/s13321-018-0286-7 PMC6041224 29995272

[51] M. Sundararajan , A. Taly , Q. Yan , *arXiv* 2016, 1611.02639.

[52] M. Sundararajan , A. Taly , Q. Yan , Int. Conf. on Machine Learning, PMLR, 2017, pp. 3319–3328.

[53] W. Tang , Y. Xiang , A. Tong , J. Org. Chem. 2009, 74, 2163. 19178143 10.1021/jo802631m

[54] V. S. Padalkar , S. Seki , Chem. Soc. Rev. 2016, 45, 169. 26506465 10.1039/c5cs00543d

[55] A. K. Singh , A. V. Nair , S. S. Shah , S. Ray , N. D. P. Singh , J. Med. Chem. 2023, 66, 3732. 36913722 10.1021/acs.jmedchem.2c01466

[56] M. Chen , L. Li , H. Nie , J. Tong , L. Yan , B. Xu , J. Z. Sun , W. Tian , Z. Zhao , A. Qin , B. Z. Tang , Chem. Sci. 2015, 6, 1932. 28717453 10.1039/c4sc03365e PMC5501095

[57] T. T. Tasso , T. Furuyama , N. Kobayashi , Chem. ‐ Eur. J. 2015, 21, 4817. 25676348 10.1002/chem.201406128

[58] S. Suzuki , S. Sasaki , A. S. Sairi , R. Iwai , B. Z. Tang , G.‐i. Konishi , Angew. Chem., Int. Ed. 2020, 59, 9856. 10.1002/anie.202000940 PMC7318703 32154630

[59] S. Sasaki , G. P. Drummen , G.‐i. Konishi , J. Mater. Chem. C 2016, 4, 2731.

[60] B. He , J. Zhang , J. Zhang , H. Zhang , X. Wu , X. Chen , K. H. S. Kei , A. Qin , H. H. Y. Sung , J. W. Y. Lam , B. Z. Tang , Adv. Sci. 2021, 8, 2004299. 10.1002/advs.202004299 PMC8025018 33854902

[61] B. Liu , B. Chu , L. Zhu , H. Zhang , W.‐Z. Yuan , Z. Zhao , W.‐M. Wan , X.‐H. Zhang , Chin. Chem. Lett. 2023, 34, 107909.

[62] H. Zhang , Z. Zhao , P. R. McGonigal , R. Ye , S. Liu , J. W. Lam , R. T. Kwok , W. Z. Yuan , J. Xie , A. L. Rogach , B. Z. Tang , Mater. Today 2020, 32, 275.

[63] A. Paszke , S. Gross , F. Massa , A. Lerer , J. Bradbury , G. Chanan , T. Killeen , Z. Lin , N. Gimelshein , L. Antiga , A. Desmaison , A. Köpf , E. Yang , Z. DeVito , M. Raison , A. Tejani , S. Chilamkurthy , B. Steiner , L. Fang , J. Bai , S. Chintala , Advances in Neural Information Processing Systems , Vol. 32 Curran Associates, Inc., Glasgow, Scotland 2019.

[64] F. Pedregosa , G. Varoquaux , A. Gramfort , V. Michel , B. Thirion , O. Grisel , M. Blondel , P. Prettenhofer , R. Weiss , V. Dubourg , J. Vanderplas , A. Passos , D. Cournapeau , M. Brucher , M. Perrot , E. Duchesnay , J. Mach. Learn. Res. 2011, 12, 2825.

[65] M. Fang , J. Yang , Q. Liao , Y. Gong , Z. Xie , Z. Chi , Q. Peng , Q. Li , Z. Li , J. Mater. Chem. C 2017, 5, 9879.

