# Supplementary material for: Deciphering Design of Aggregation‐Induced Emission Materials by Data Interpretation
Source: Adv Sci (Weinh). 2024 Nov 22;12(3):2411345. doi: 10.1002/advs.202411345 (PMC11744700; doi:10.1002/advs.202411345)
Supplement: Supplementary file 1 — Supporting Information [file ADVS-12-2411345-s001.doc]

**Supporting Information**

**Top-down Strategy Enabling Elastic** **Wood Nanocarbon Sponges with Wrinkled Multilayer Structure and High Compressive Strength for High-performance** **Compressible Supercapacitors**

Song Wei,^a,b^ Caichao Wan,*^a^ Shanshan Jia,^c^ Xingong Li,^a^ Ruwei Chen,^b^ Guanjie He,*^b^ Yiqiang Wu*^a^

*^a^ College of Materials Science and Engineering, Central South University of Forestry and Technology, Changsha 410004, China.*

*^b^ Christopher Ingold Laboratory, Department of Chemistry, University College London, London, WC1H0A, UK.*

*^c^* *College of Forestry, Sichuan Agricultural University, Chengdu 611130, P. R. China.*

*** *Corresponding authors. E-mail: wancaichaojy@163.com (C.W.); g.he@ucl.ac.uk (G.H.); wuyq0506@126.com (Y.W.)*

**Supplementary Figures**


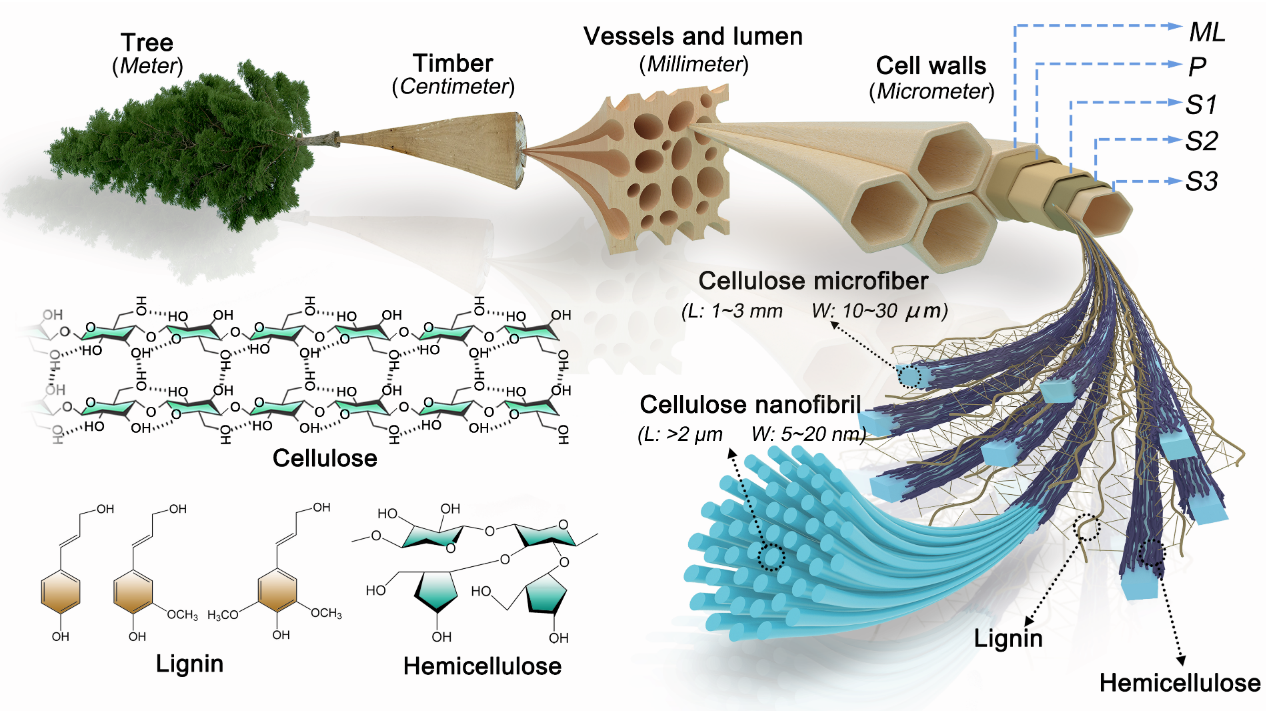


**Fig. S1** Schematic illustration of cellular structure and chemical composites of natural wood, reproduced with permission.^[1]^ Copyright 2023, Royal Chemistry Society.


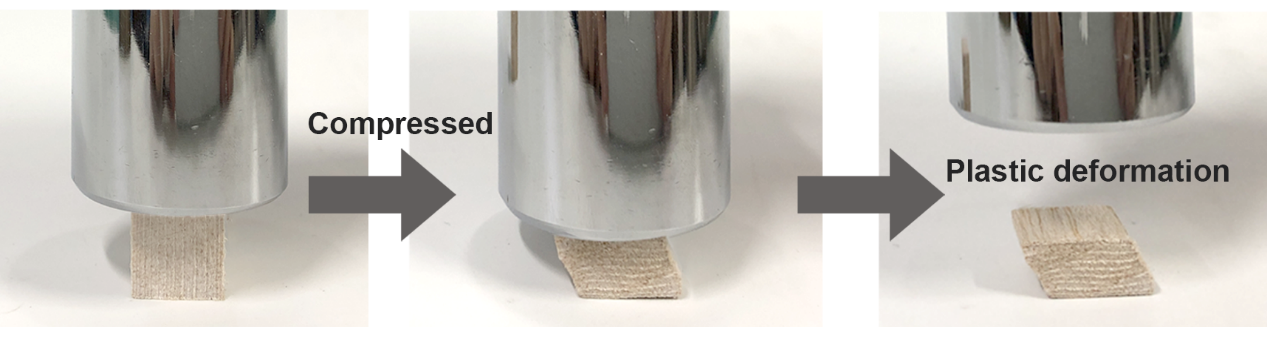


**Fig. S2** Digital images show a block of compressed Balsa wood without shape recovery.


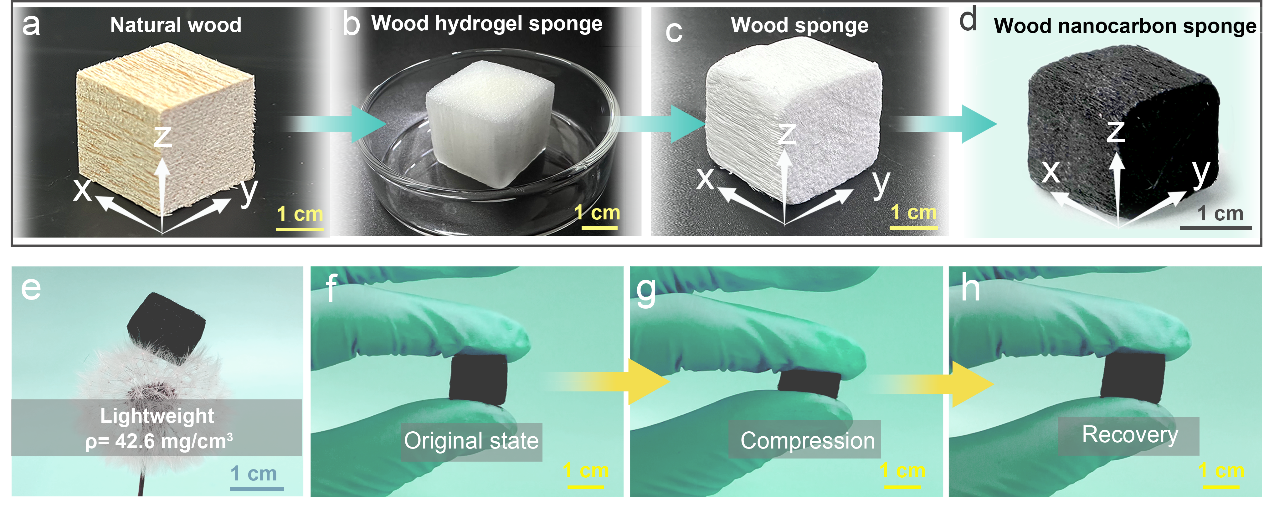


**Fig. S3** a–d) Macroscopic morphological transformation from natural wood to WNCS during the fabrication processes. Digital images showing (e) the lightweight WNCS supported by dandelion and (f–g) its reversible compressibility.

Fig. S3a–d recorded the macromorphologies of the sample at different producing stages, including the faint yellow natural wood block, the wood hydrogel after delignification (Fig. S3a), the wood sponge after freeze-dring (Fig. S3b) and wood nanocarbon sponge after carbonization (Fig. S3c). It can be found that the wood hydrogel and sponge obtained after chemical delignification are pure white, indicating the complete removal of lignin and hemicellulose. The density of the wood sponge (34.2 mg cm^−3^) is almost a third compared to that of the natural wood (93.9 mg cm^−3^), demonstrating the removal of non-cellulose components since the cellulose content in the wood is about 30%. As a result of the volume reduction (≈45.4% shrinkage rate) following carbonization, the WNCS experiences a slightly elevated density of 42.6 mg cm^−3^. Fig. S3f–g shows the compression-resilience process of the WNCS on the XY plane, which corresponds to axial section-II shown in Fig. 1c.


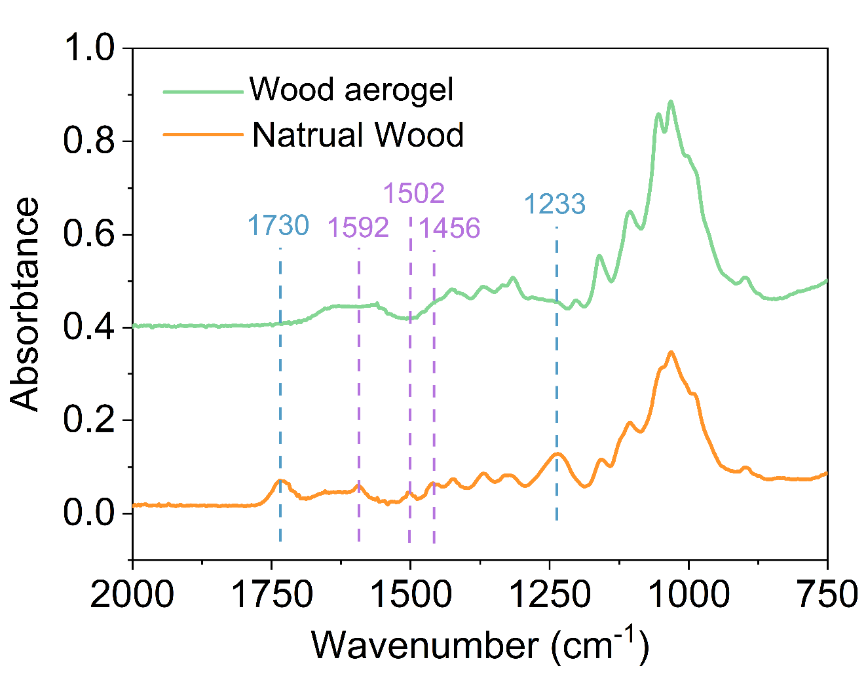


**Fig. S4** FTIR spectra of natural wood and wood sponge. The absorption peaks at 1730 and 1233 cm^−1^ belong to the stretching vibration of C=O and C-O for hemicellulose, respectively. The absorption peaks at 1592 and 1502 cm^−1^ are aromatic skeletal vibrations and 1456 is the bending vibration of C-H for lignin, respectively.^[2]^


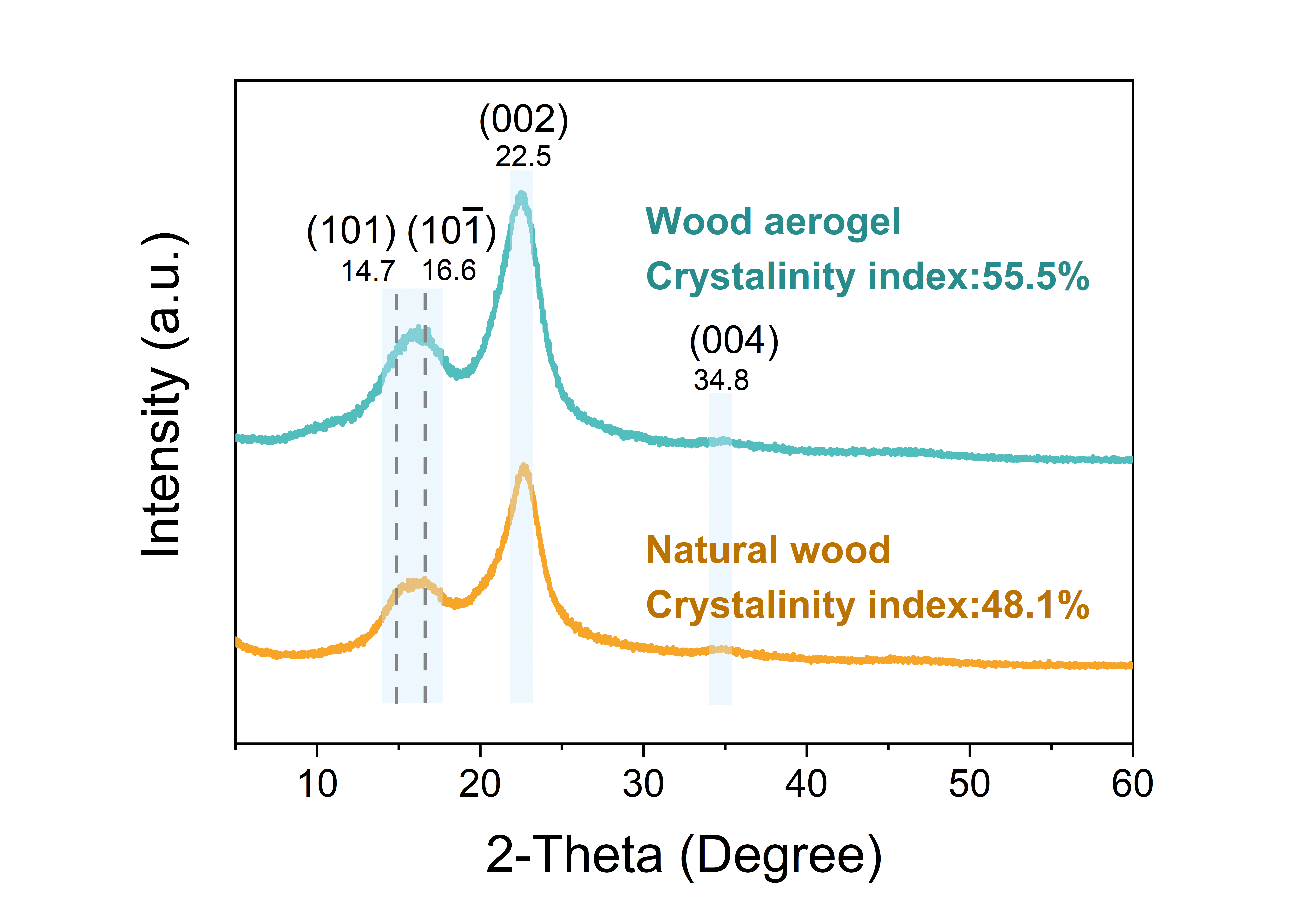


**Fig. S5** XRD pattern of natural wood and wood sponge.


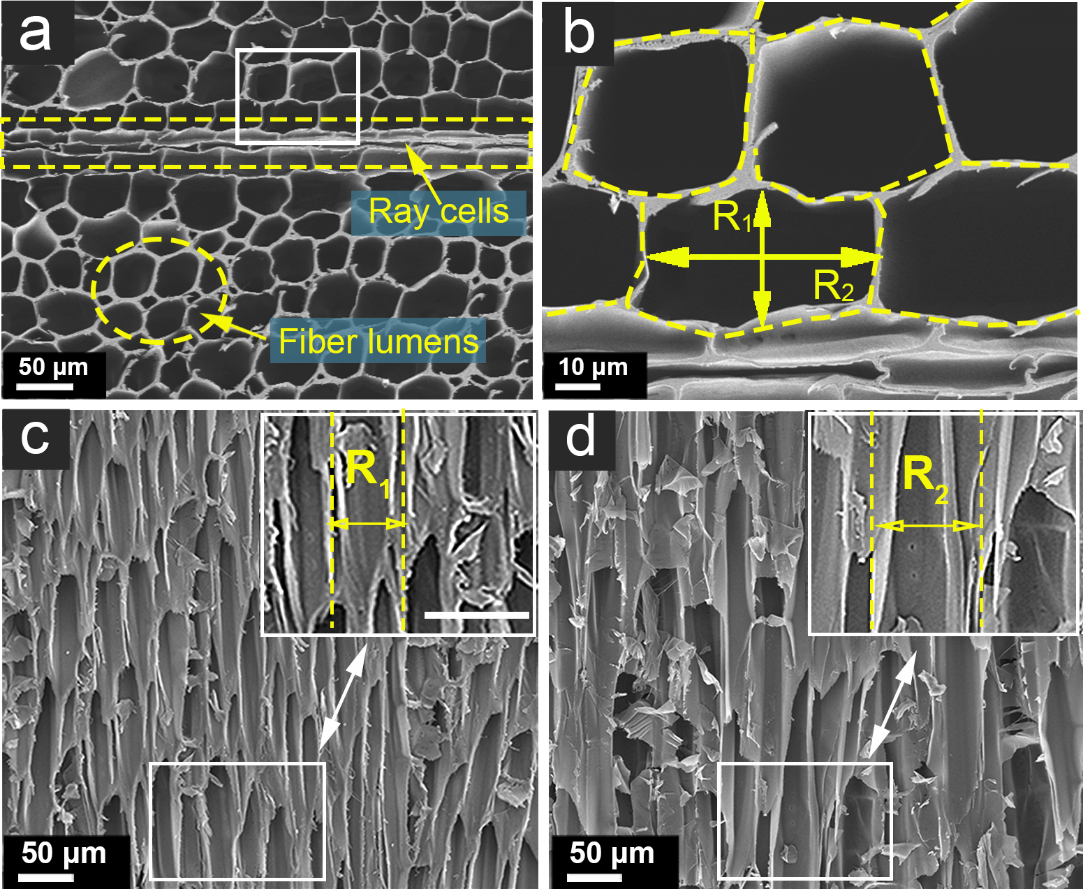


**Fig. S6** SEM image of natural wood on transection (a, b), axial section-I (c), and axial section-II (d).

Fig. S6a shows the neatly arranged honeycomb-like fiber lumens and ray cells of natural *Balsa* wood. Further expanding the resolution, it can be observed that these fiber cavities have thinner cell wall layers and high porosity (Fig. S6b), which is one of the important reasons why balsa wood is easily permeable to chemical reagents.^[3]^ On axial section-I (Fig. S6c) and axial section-II (Fig. S6d), the straight inner wall structure corresponding to these honeycomb-like fiber channels can be observed. It is worth noting that the diameter (R_2_) of the pores shown in axial section-II is larger than that (R_1_) of section-I, indicating that these fiber cells tend to be elongated, consistent with the observation on transection (Fig. S6b).


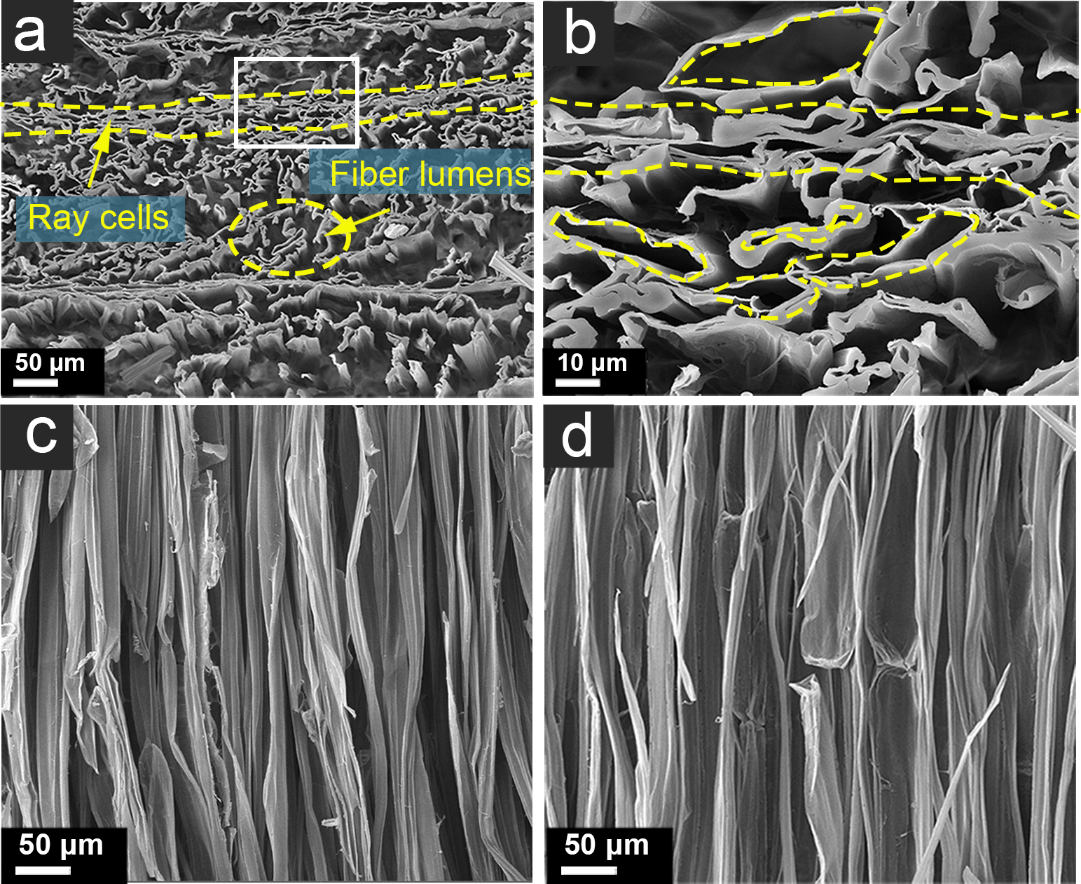


**Fig. S7** SEM image of wood sponge on transection (a, b), axial section-I (c), and axial section-II (d).

After the dissolution and removal of lignin and hemicellulose, the structures of all wood cells underwent significant variation (Fig. S7a). As shown in Fig. S7b, the region of ray cells evolved into a continuous multilayer structure, while the fiber lumens disengaged from each other, either forming independent irregular flat tubes or disassembling into wrinkled, layered, and interlaced structures. This is due to the destruction of the cellular structures after the removal of the fillers within cellulose microfibers, i.e. lignin and hemicellulose that play a bonding role in the cell wall. The retained cellulose microfibril aggregation layers are interlaced to form continuous wrinkled layers. The SEM images of two axial sections in Fig. S7c and S7d further confirmed such unique wrinkled multilayers of wood sponge.


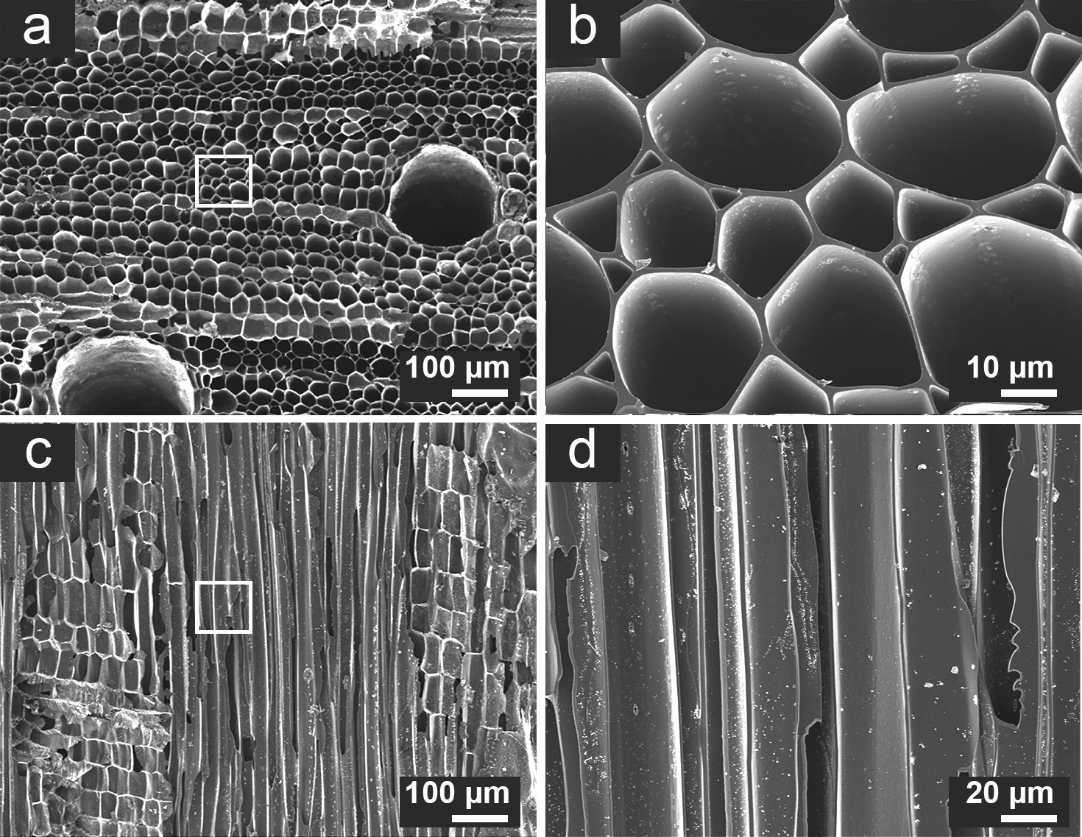


**Fig. S8** SEM images of wood carbon at different sections. a, b) Transecti, c, d) axial section**.**


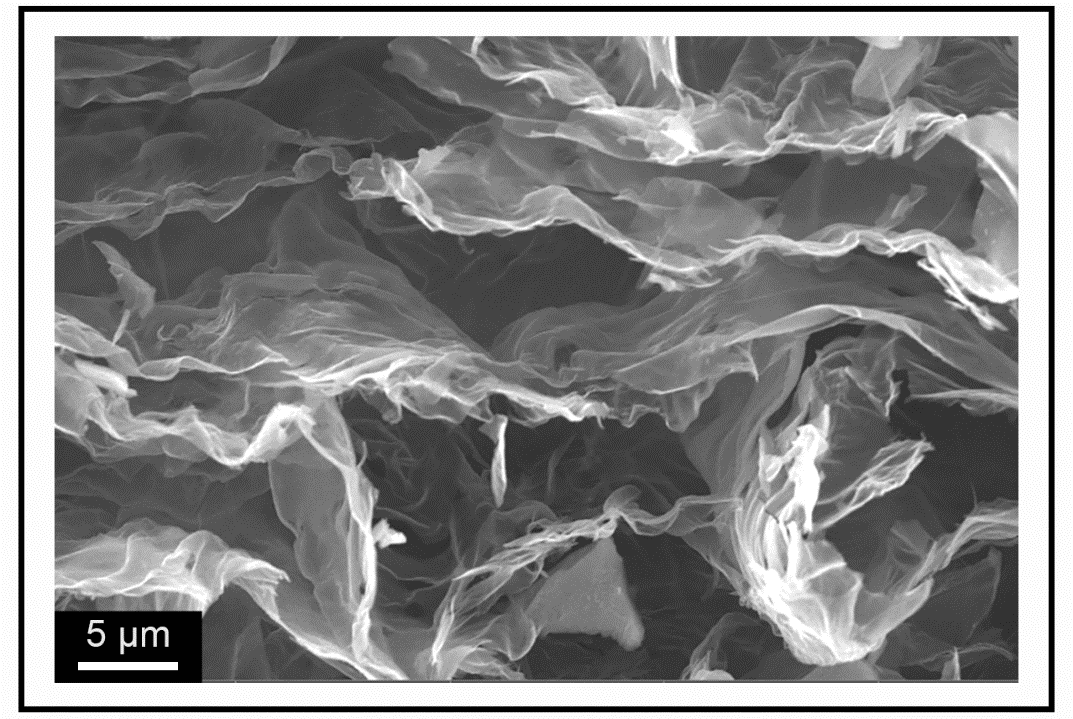


**Fig. S9** SEM image of WNCS at large magnification, showing the wrinkled lamellar nanocarbons.


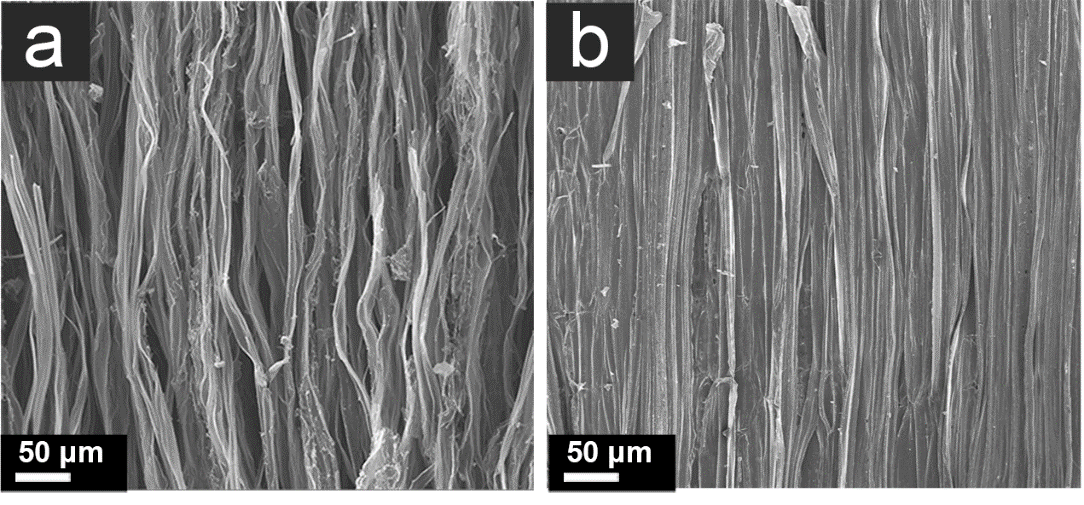


**Fig. S10** SEM image of WNCS on (a) axial section-I and (b) axial section-II.


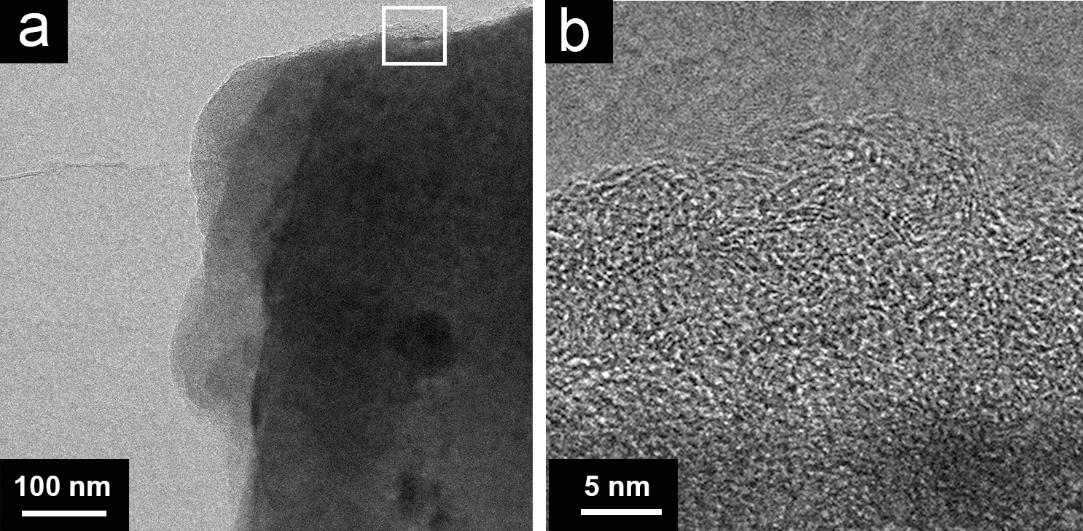


**Fig. S11** TEM images of wood carbon at different magnifications.


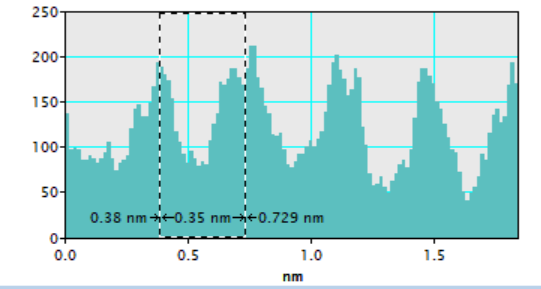


**Fig. S12** The intensity line profiles of the selected area from Fig. 3b.


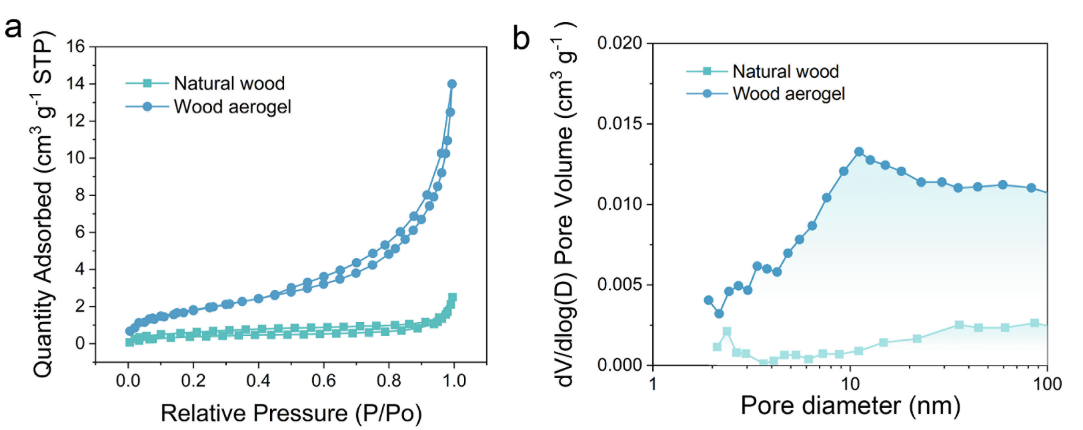


**Fig. S13** a) N_2_ adsorption-desorption isotherms and (b) pore distribution profiles of natural wood and wood sponge.

As shown in Fig. 12a, the nitrogen adsorption-desorption isotherm of natural Balsa wood exhibits minimal adsorption (2.5 cm^−3^ g^−1^) within the range of 0 < P/P_0_ < 1.0, as natural wood is dominated by macropores (50 – 7500 nm) in the fiber lumens and few mesopores and micropores in the cell wall layers. After the removal of lignin and hemicellulose, the nitrogen adsorption capacity of wood sponges was significantly increased (14.0 cm^−3^ g^−1^) in the same relative pressure range. In particular, the adsorption capacity increases sharply within the range of P/P_0_ > 0.6, indicating a significant increase in the number of mesopores and macropores in the structure. This conclusion can be also confirmed by pore distribution profiles in Fig. S12b.


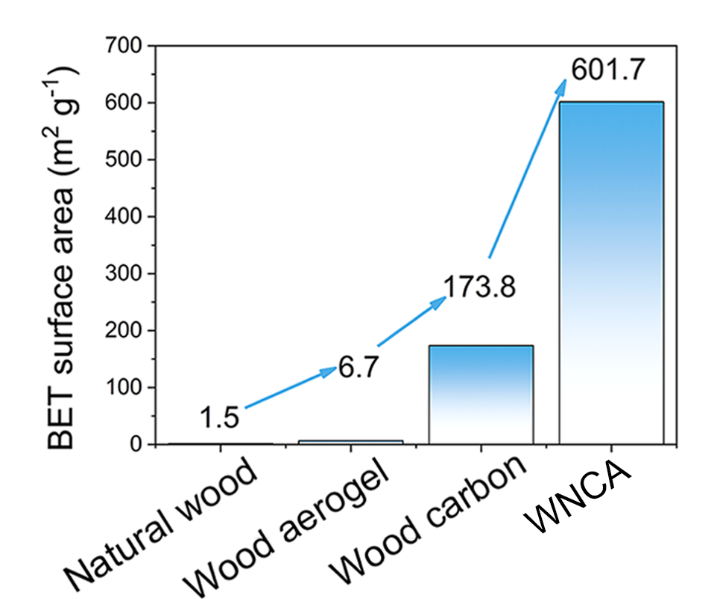


**Fig. S14** Comparison of specific surface areas for various samples.


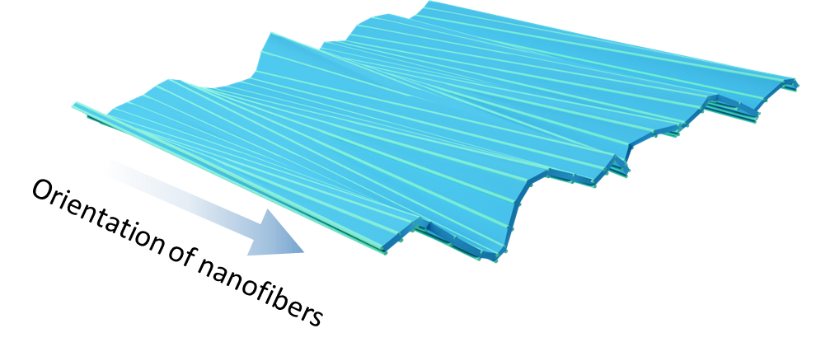


**Fig. S15** The orientation of nanofibers in a wrinkled layer.

**
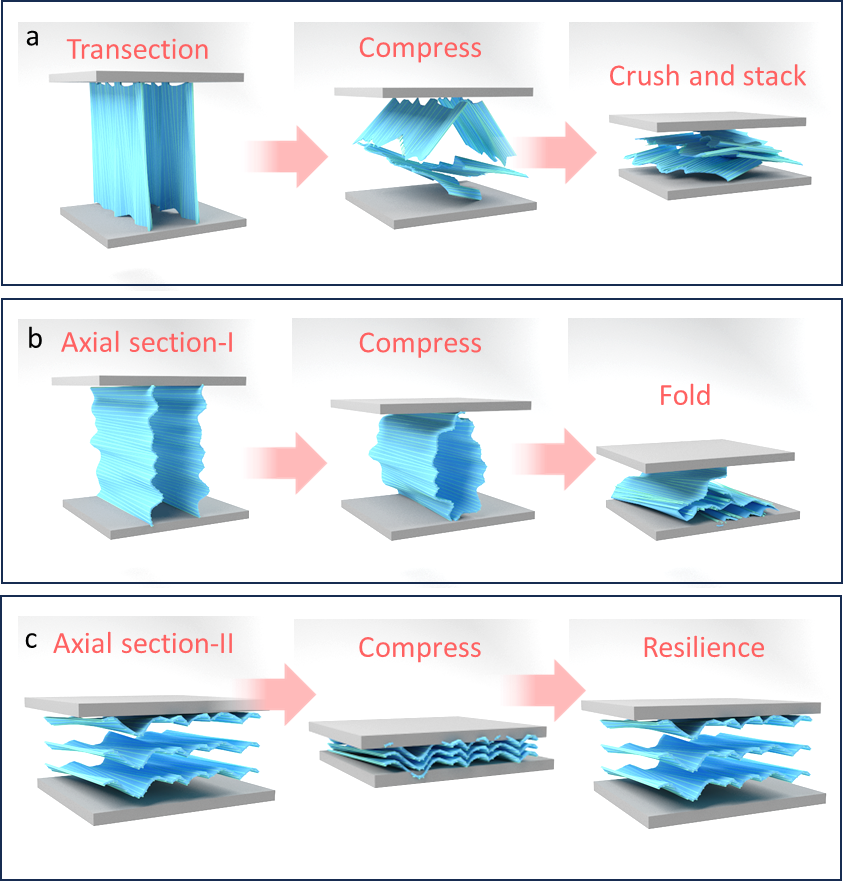
**

**Fig. S16** Schematic illustrations of the compression processes on various direction of WNCS, a) transection, b) axial section-I and (c) axial section-II.


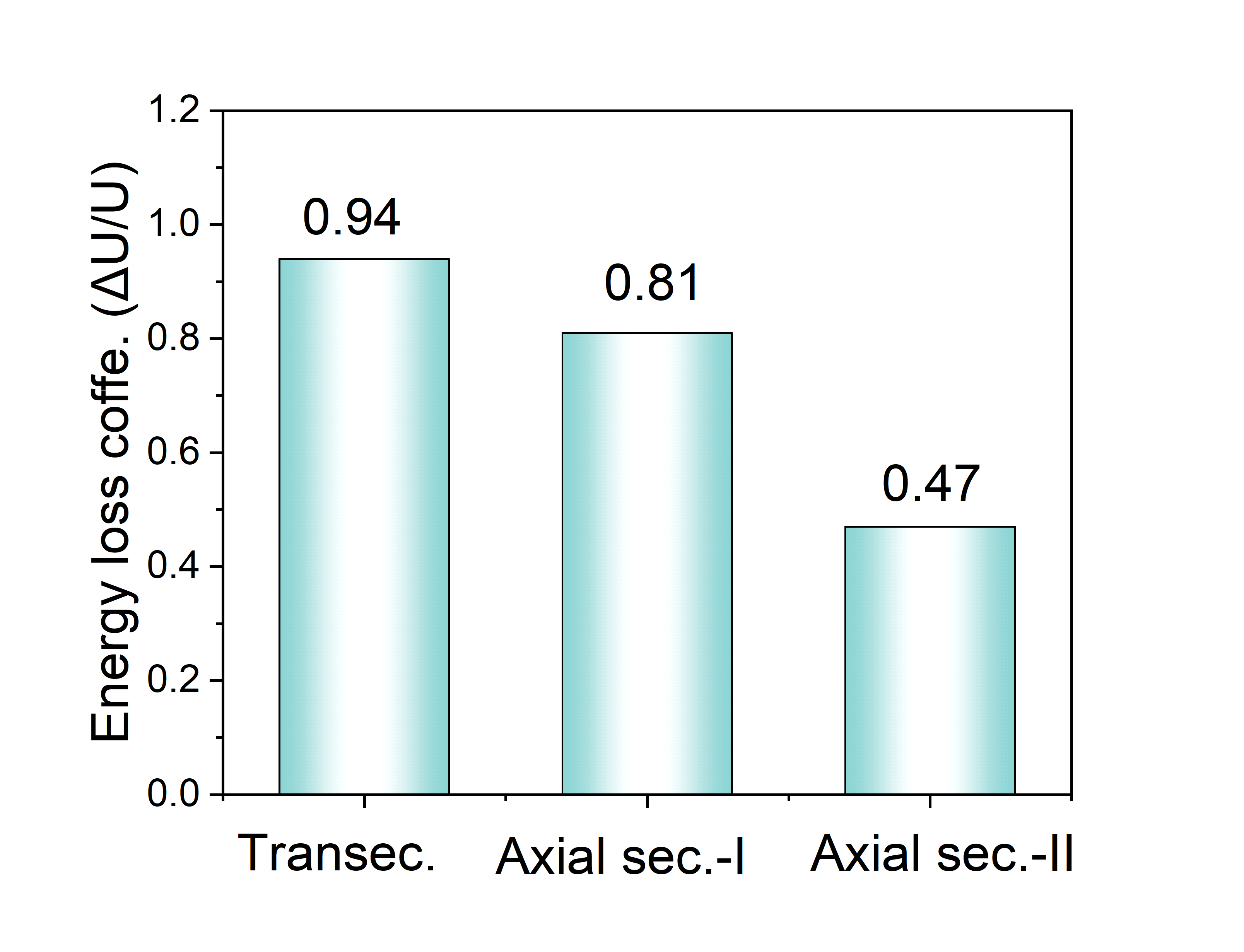


**Fig. S17** Energy loss coefficient at 40% strain for WNCS compressed on different sections.


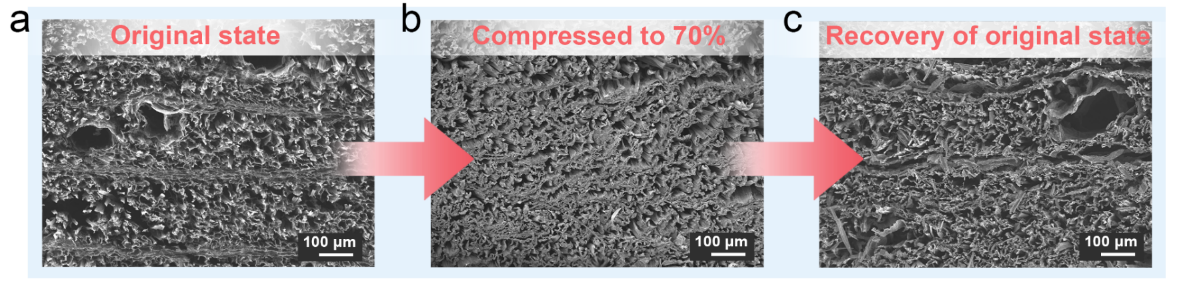


**Fig. S18** SEM images showing the morphological transformation of elastic WNCS during 70% strain compression. No obvious loosening or cracking appeared even under severe structural densification.


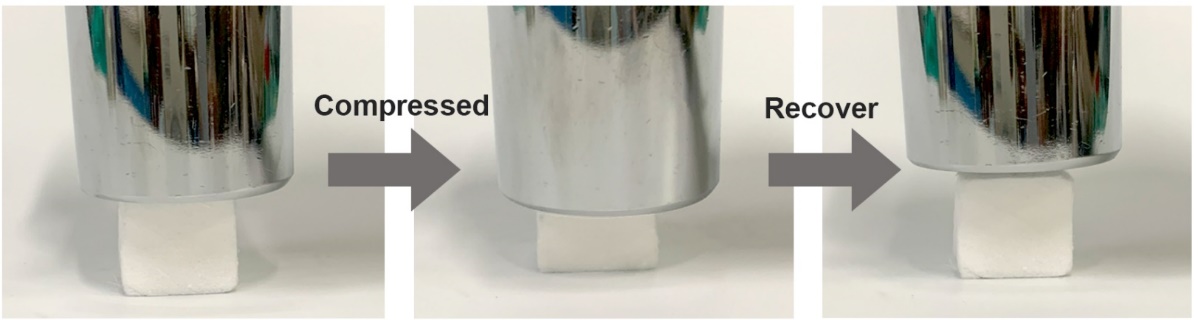


**Fig. S19** Digital images show the compression-recovery process of wood sponges at a strain of 50%.


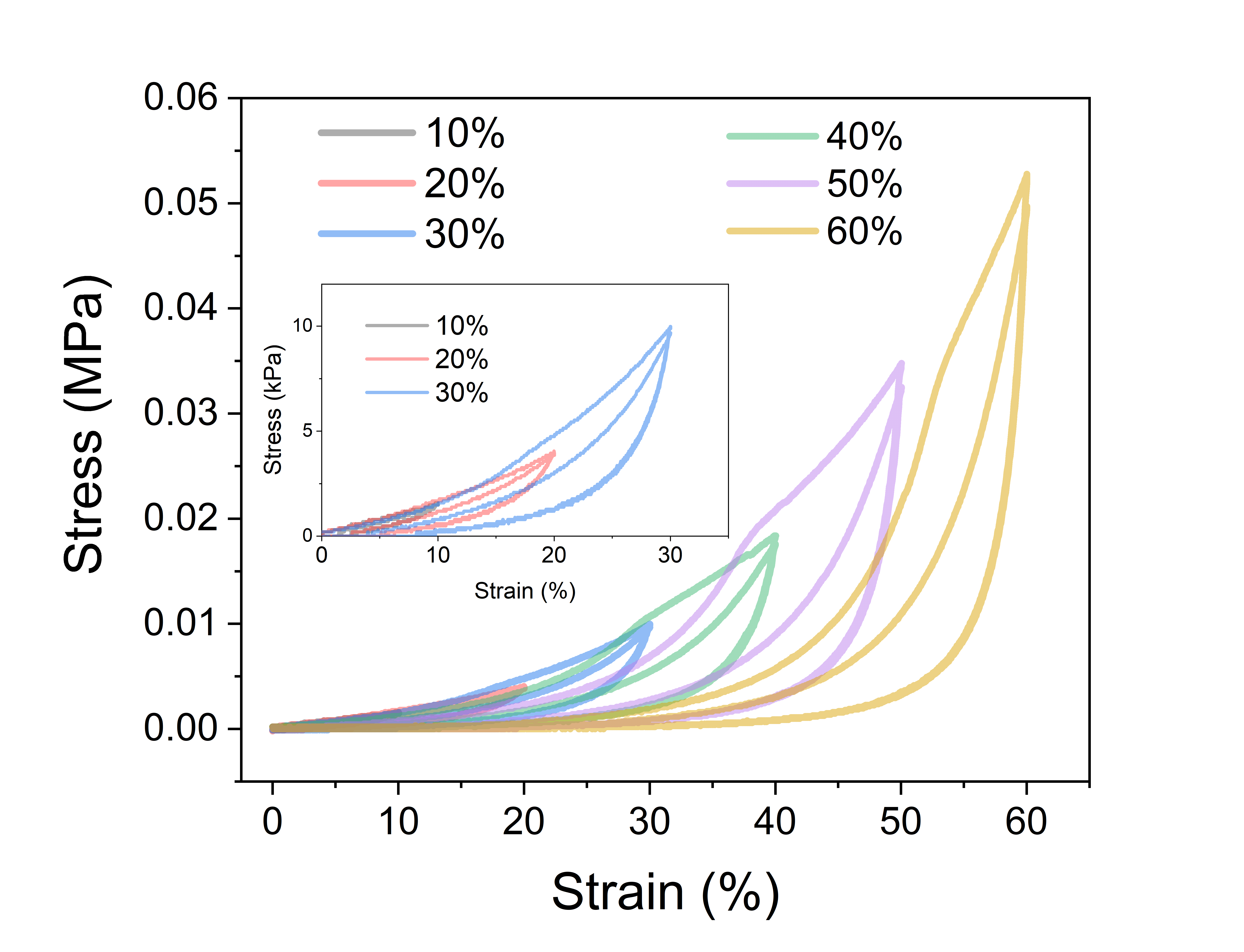


**Fig. S20** *σ*-*ε* curves of wood sponge at the strain ranges from 10% to 60%.


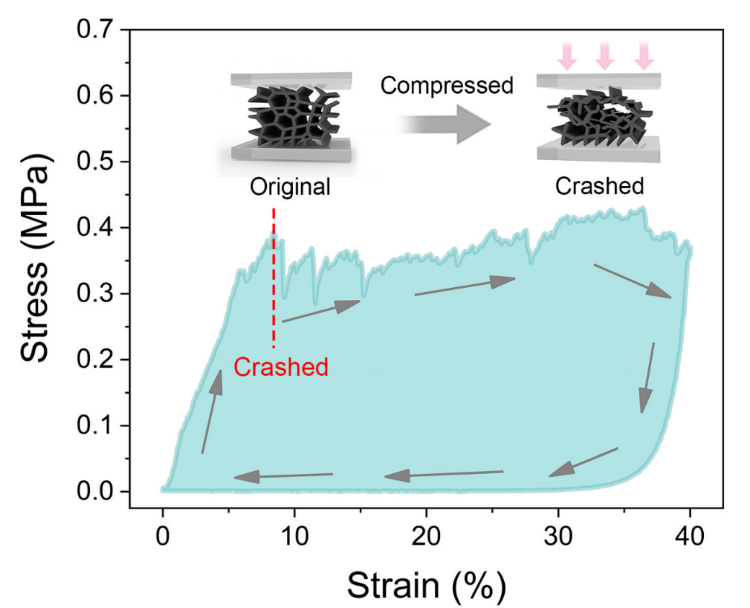


**Fig. S21** *σ*-*ε* curve at 40% strain of wood carbon.


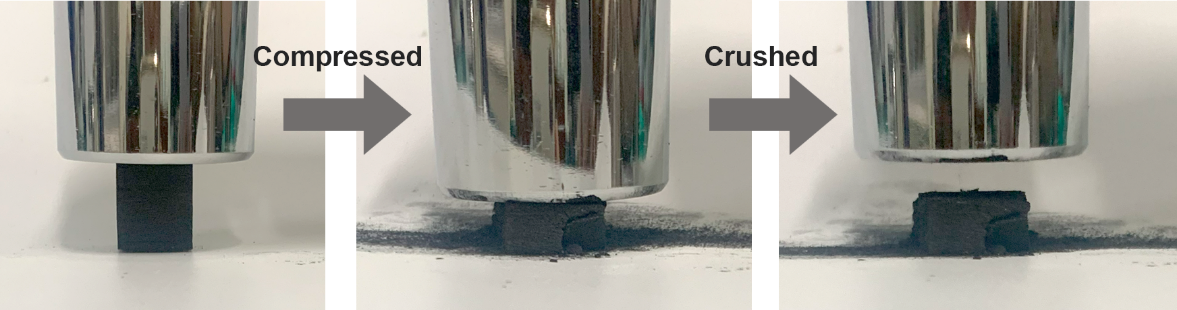


**Fig. S22** Digital images of the wood carbon crushed during the compression process.


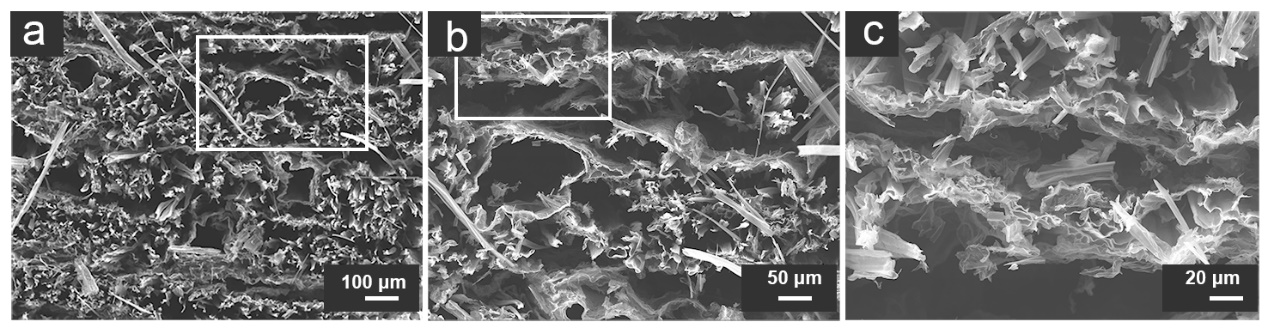


**Fig. S23** SEM images at different magnifications of WNCS after 1000 cycles compressing at 60% strain.


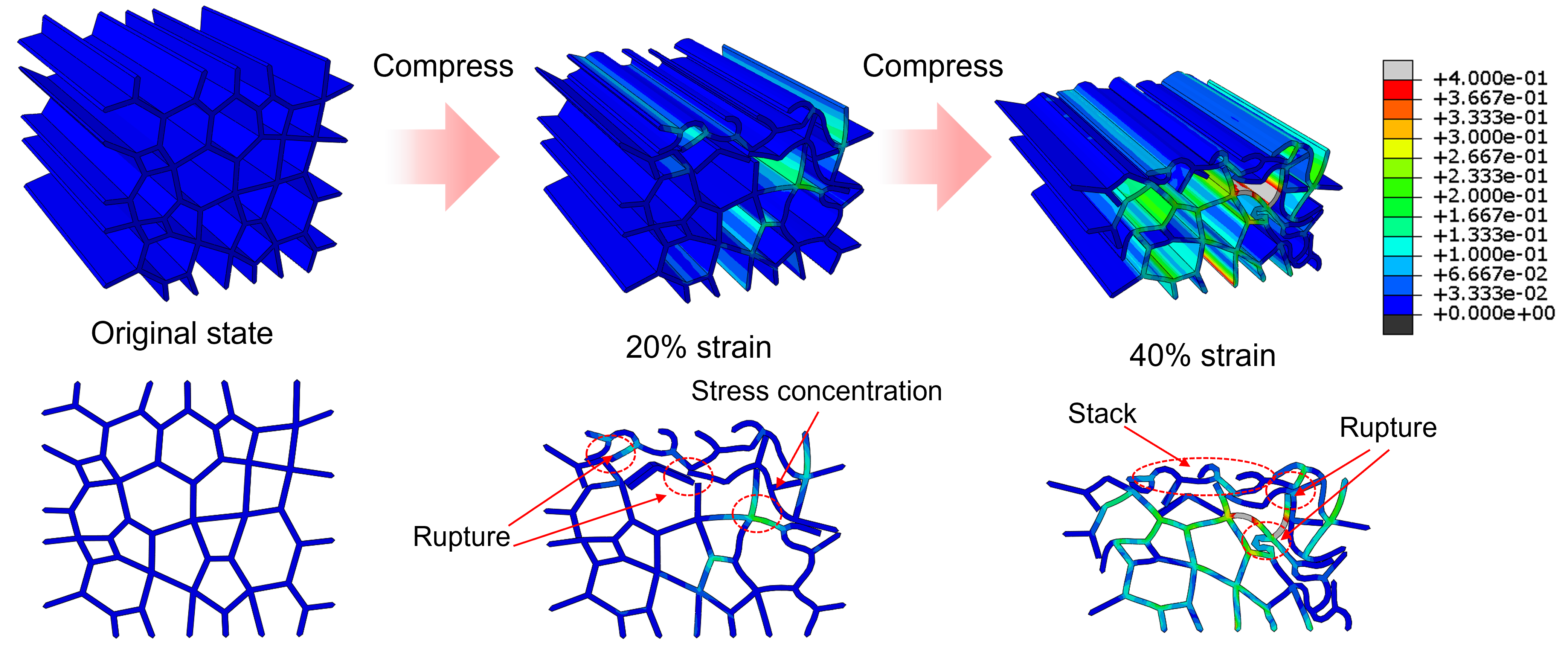


**Fig. S24** FE simulation of the compression-release process in the axial section-II of the wood carbon. The models of wood carbon show both 3D perspectives and 2D front views under the original state, 20% strain, and 40% strain.


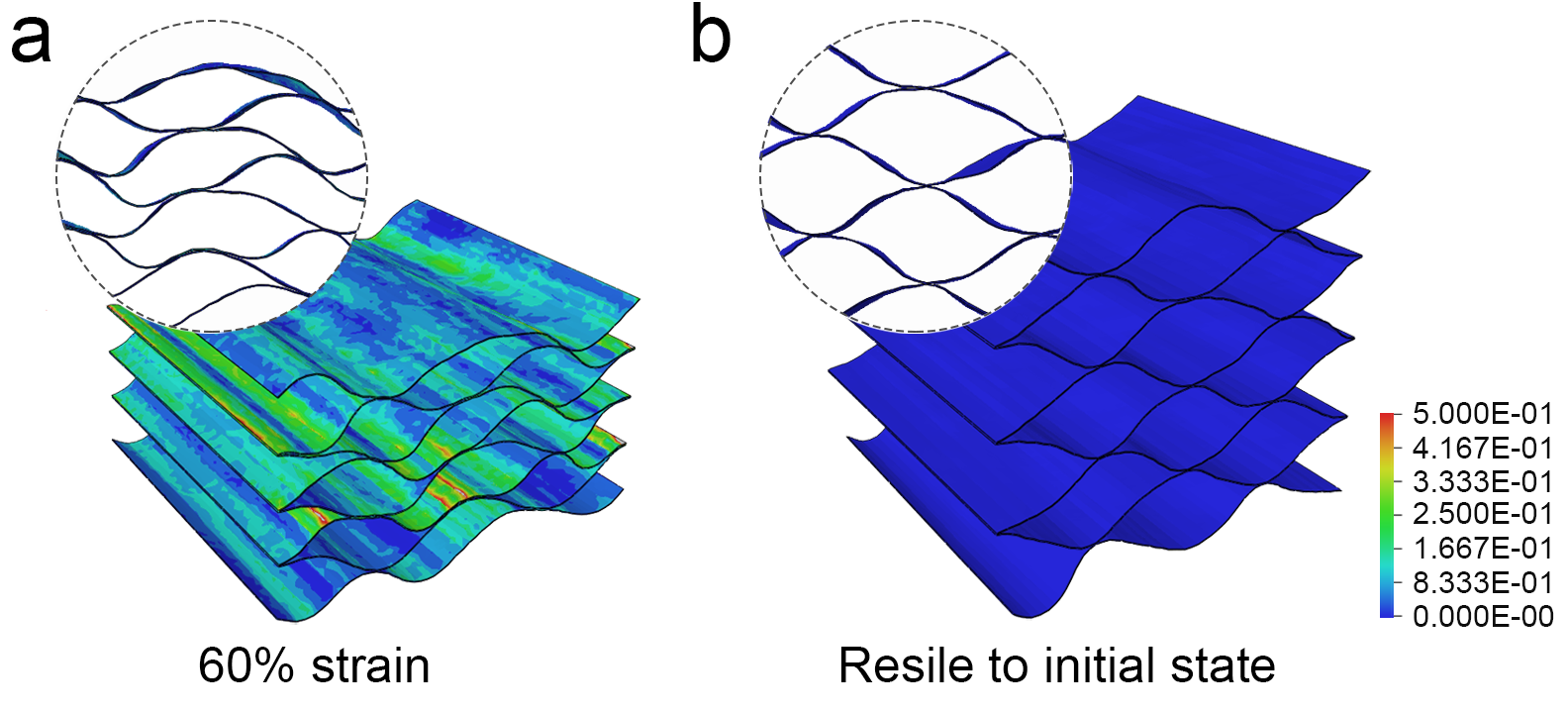


**Fig. S25** FE simulation of the compression process in the axial section-II of the WNCS at 60% and resile to initial state.


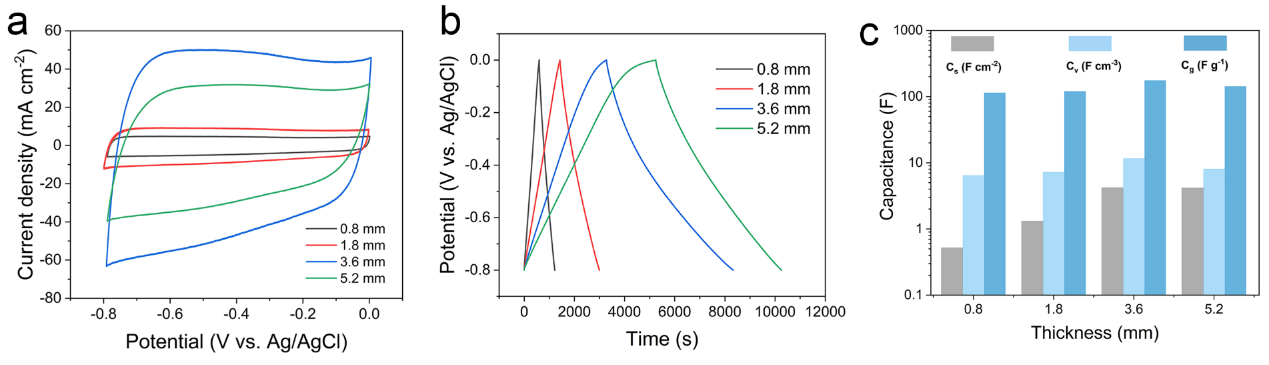


**Fig. S26** Comparison of (a) CV curves at 5 mV s^−1^, b) GCD curves at 1 mA cm^−2^, and (c) specific capacitances for WNCS electrodes with different thicknesses.


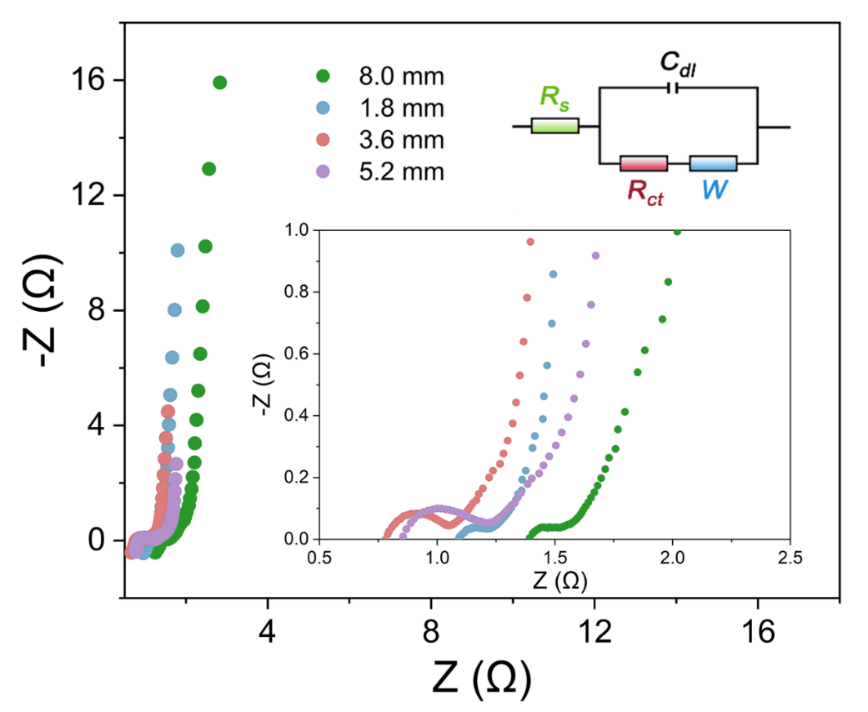


**Fig. S27** Nyquist plots for WNCS electrodes of varying thicknesses.


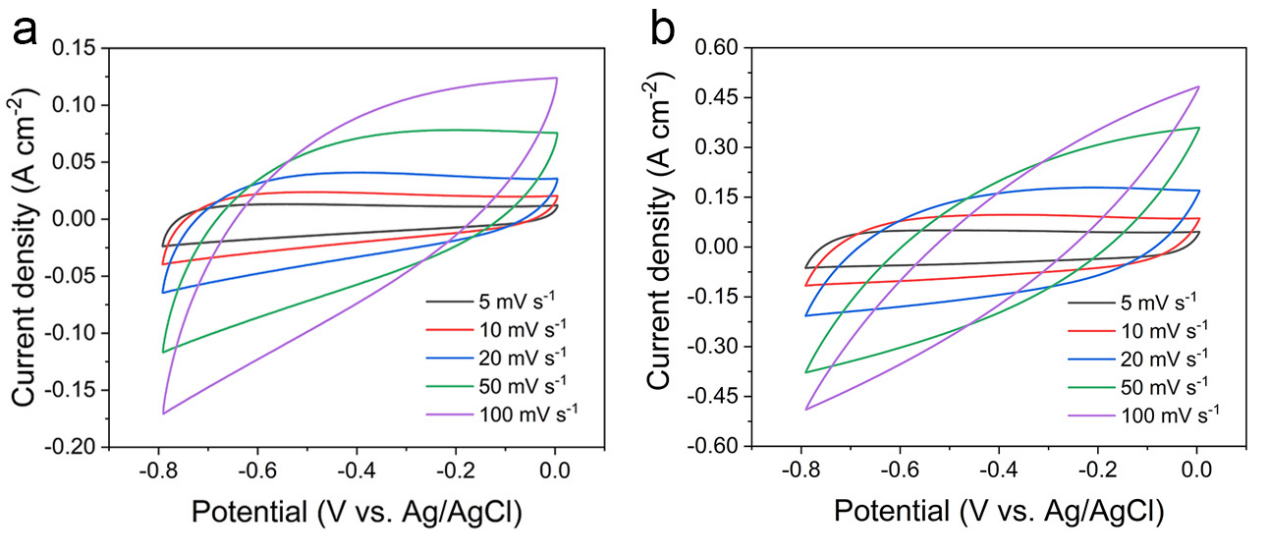


**Fig. S28** CV curves of (a) wood carbon and (b) WNCS at different scan rates.


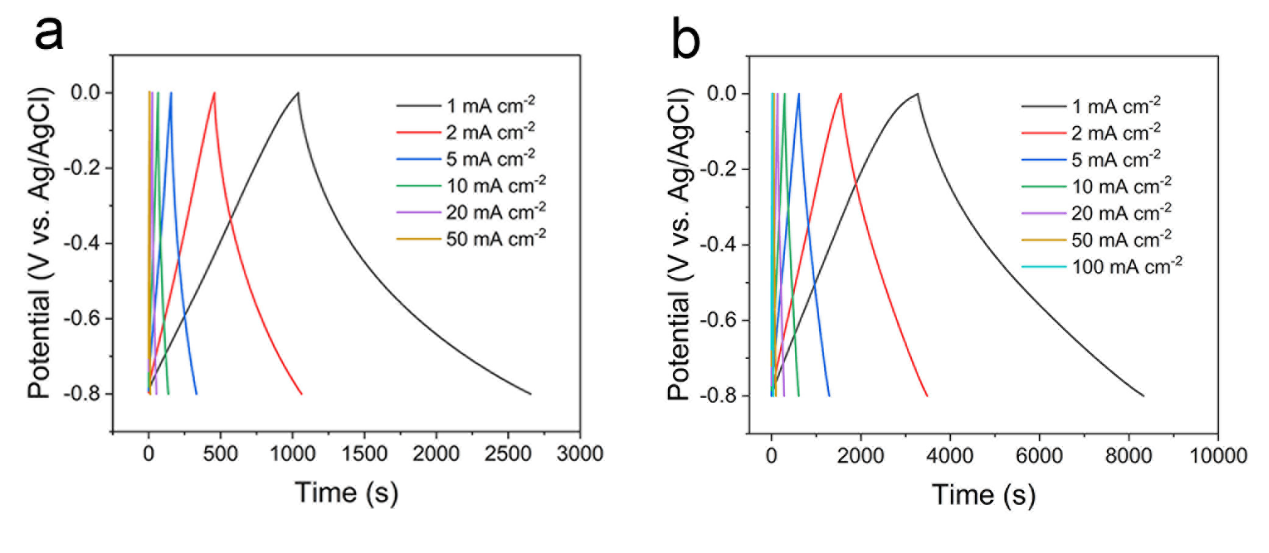


**Fig. S29** GCD curves of (a) wood carbon and (b) WNCS at different current density.


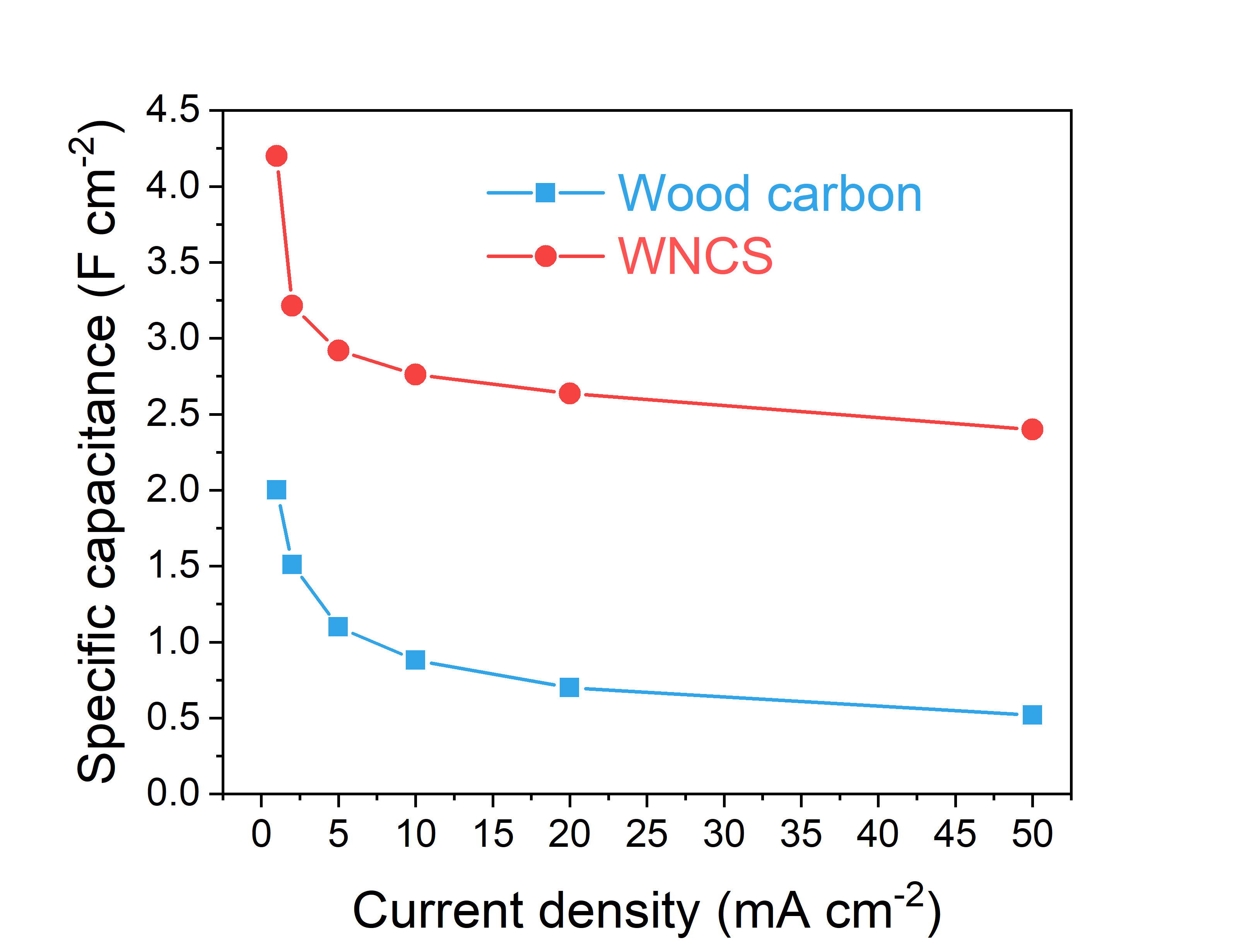


**Fig. S30** Comparison of area specific capacitances of wood carbon and WNCS.

**Fig. S31** Electrical conductivity of wood carbon and WNCS.


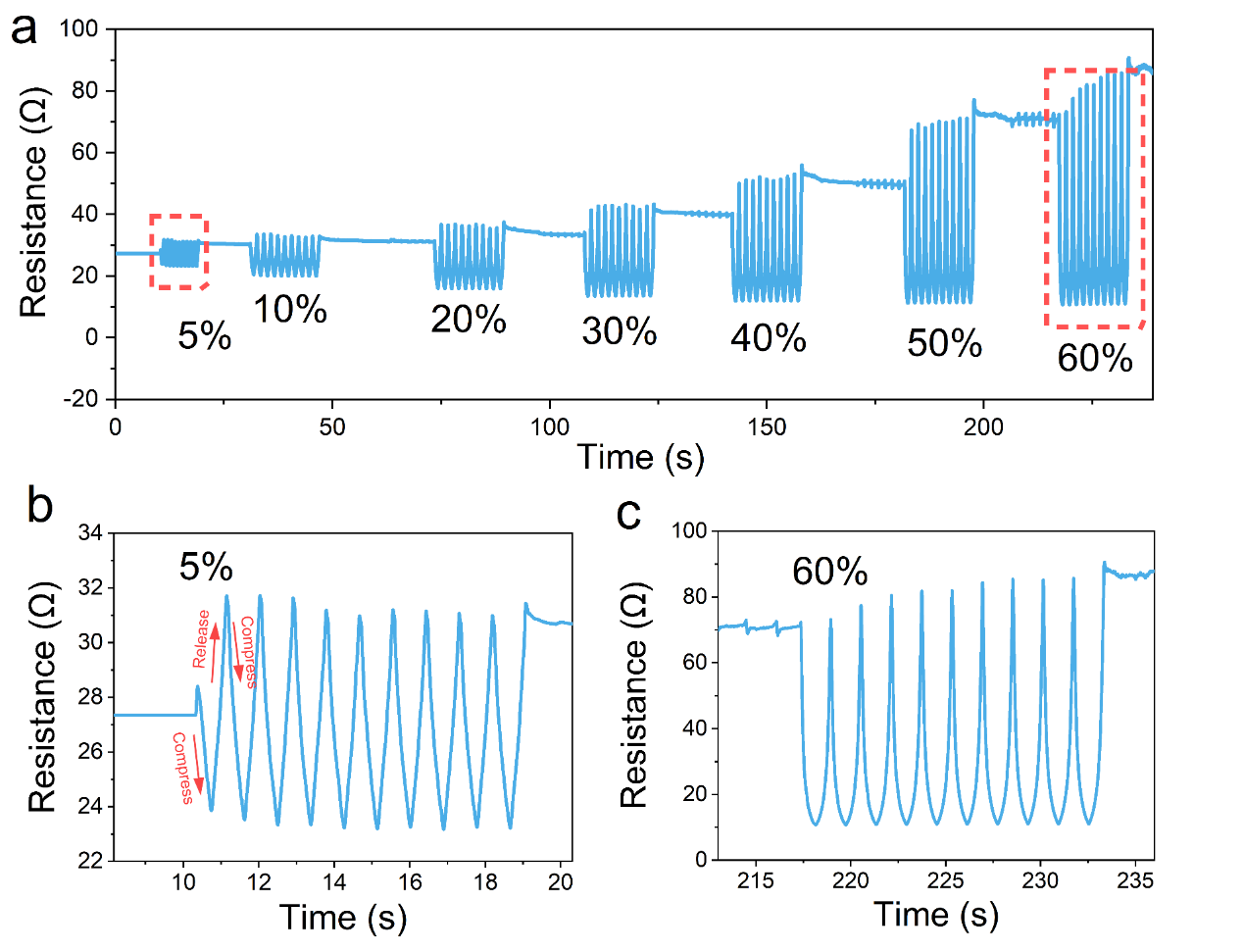


**Fig. S32** Resistance variation of the WNCS with increasing compressive strain. a) the curve of resistance changes in the range of 0-60% strain, each compressive test repeats 10 times. The curves of resistance change focusing under the strain of (b) 5% and (c) 60%.


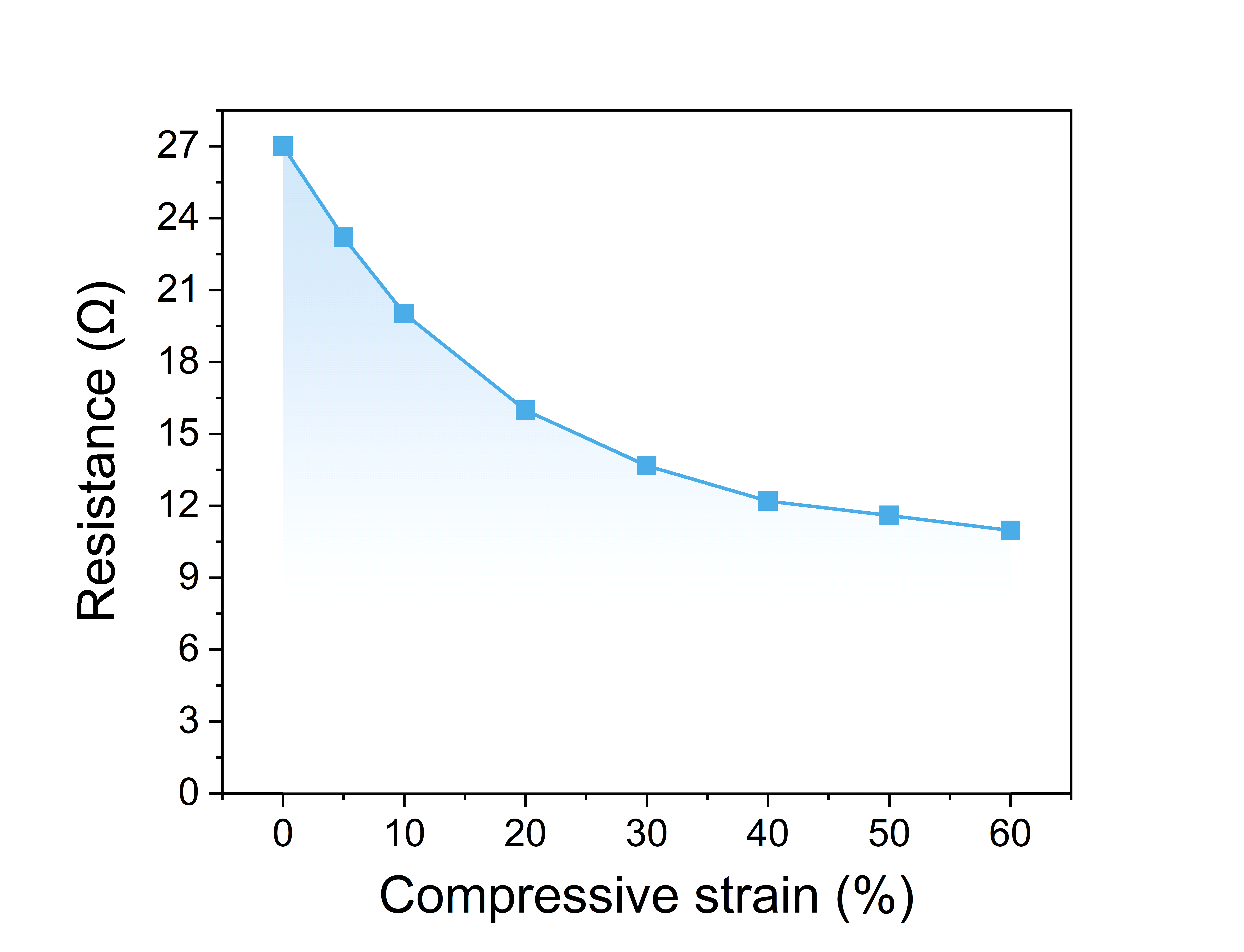


**Fig. S33** Electrical resistance variation of the WNCS under increasing compressive strains


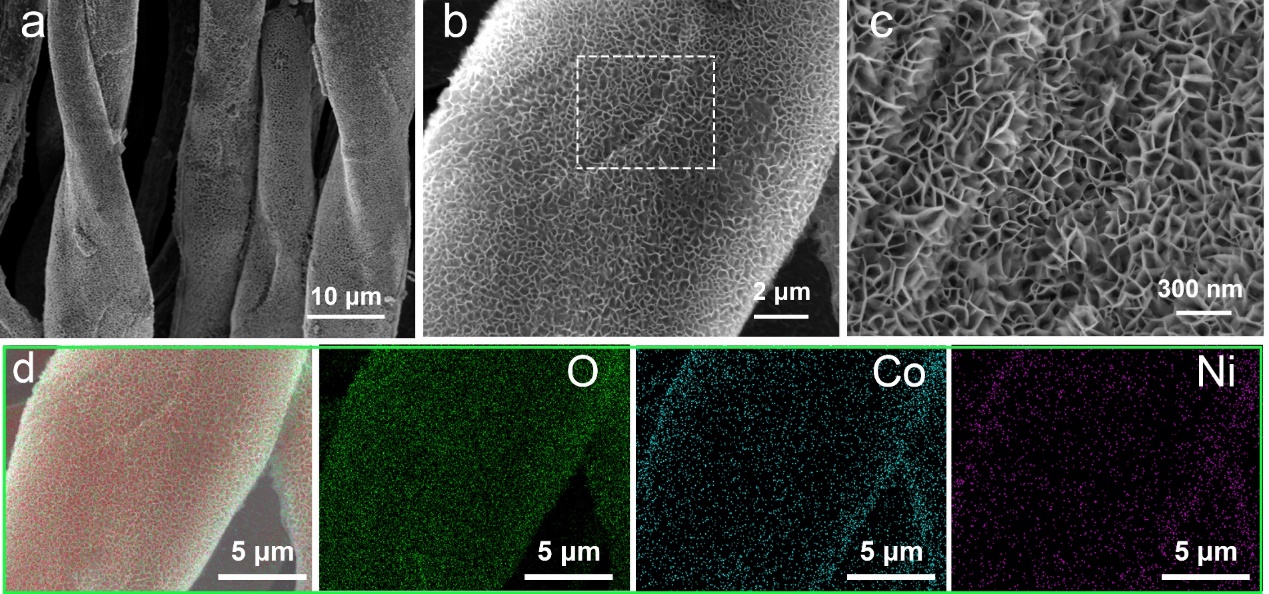


**Fig. S34** a–c) SEM images of the NiCo LDH/CFs electrode at different magnifications. d) Bright-field STEM image and EDS elemental mappings of O, Co, and Ni.


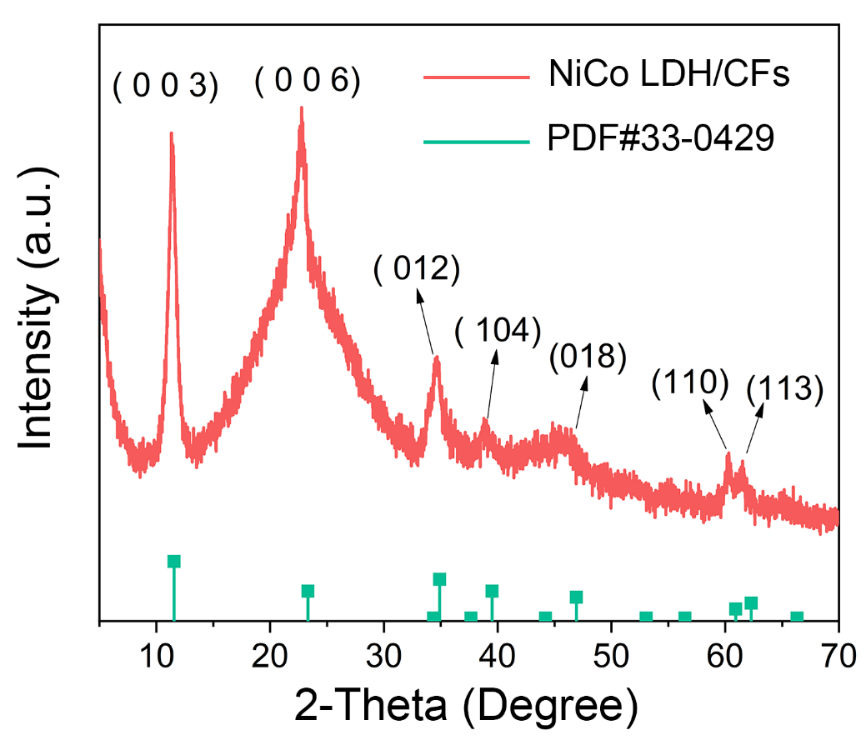


**Fig. S35** XRD pattern of NiCo LDH/CFs and the standard card of nickel–cobalt layered double hydroxide (PDF#33-0429).


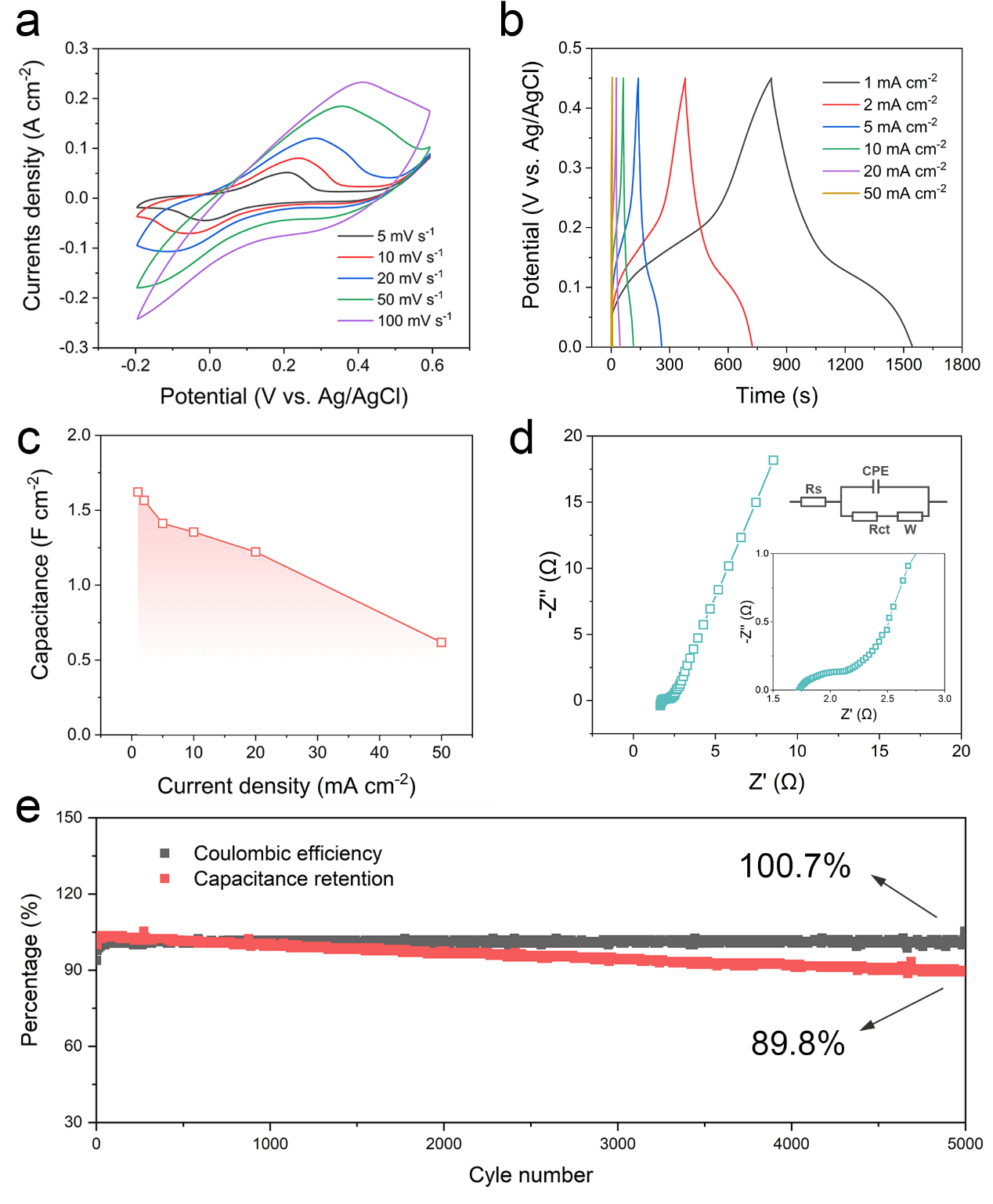


**Fig. S36** Electrochemical properties of NiCo LDH/CFs electrodes: a) CV curves, b) GCD curves, c) specific capacitances, d) Nyquist plot, and **e** cycling stability.


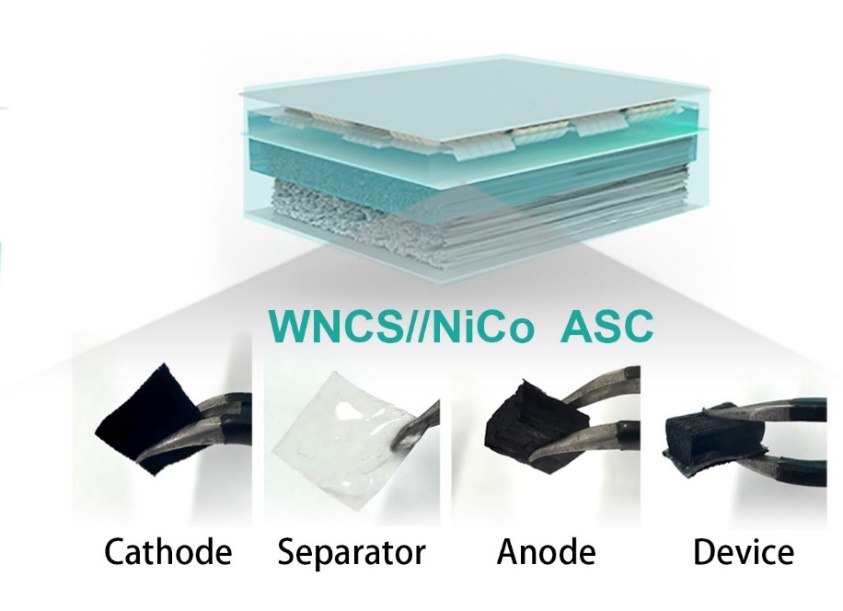


**Fig. S37** Schematic illustrations and digital images of assembly of the sandwich-like structure ASC device.


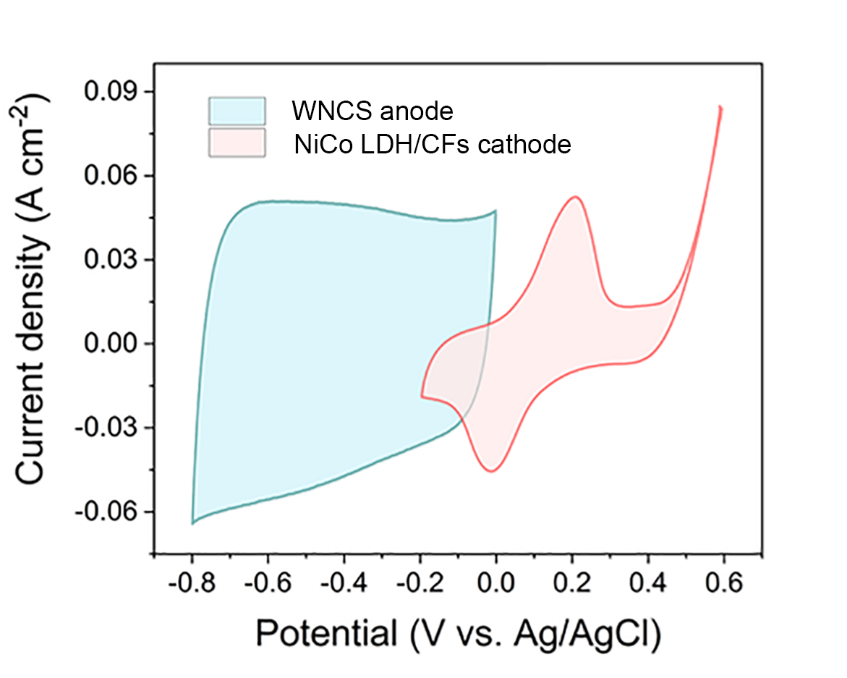


**Fig. S38** CV curves of the WNCS anode and NiCo LDH/CFs cathode at 5 mV s^−1^.


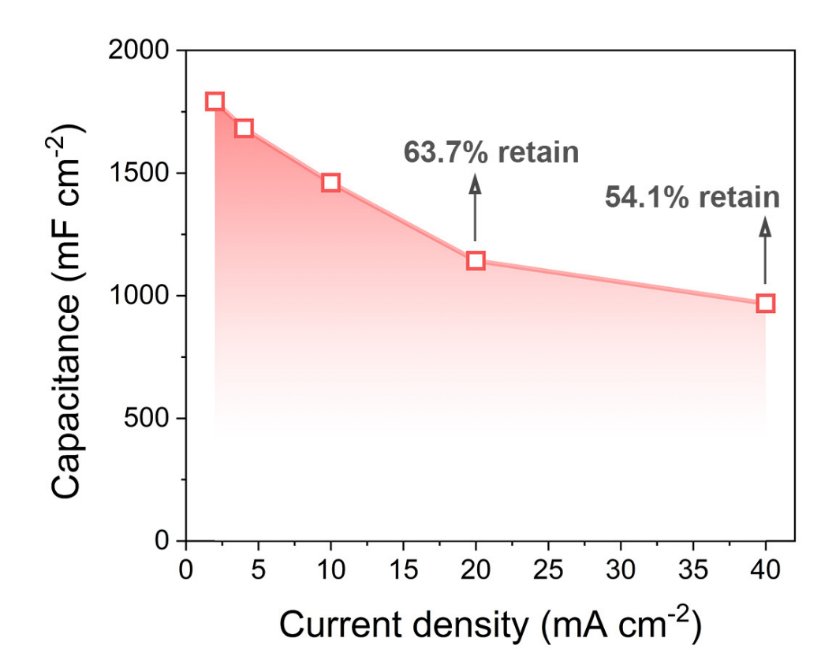


**Fig. S39** Specific areal capacitances of WNCS//NiCo ASC at different current densities.


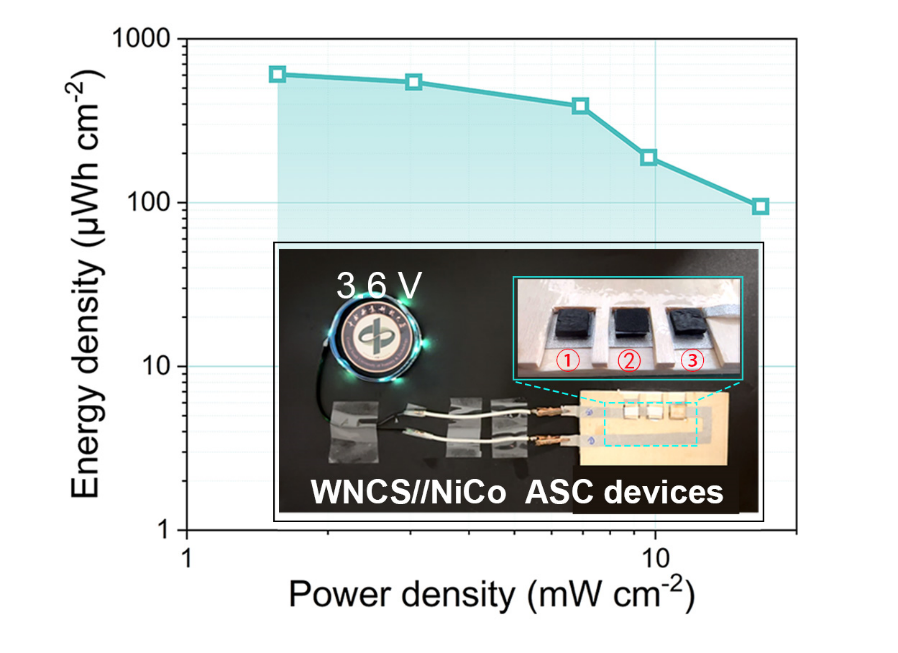


**Fig. S40** Energy densities and power densities of WNCS//NiCo ASC device.


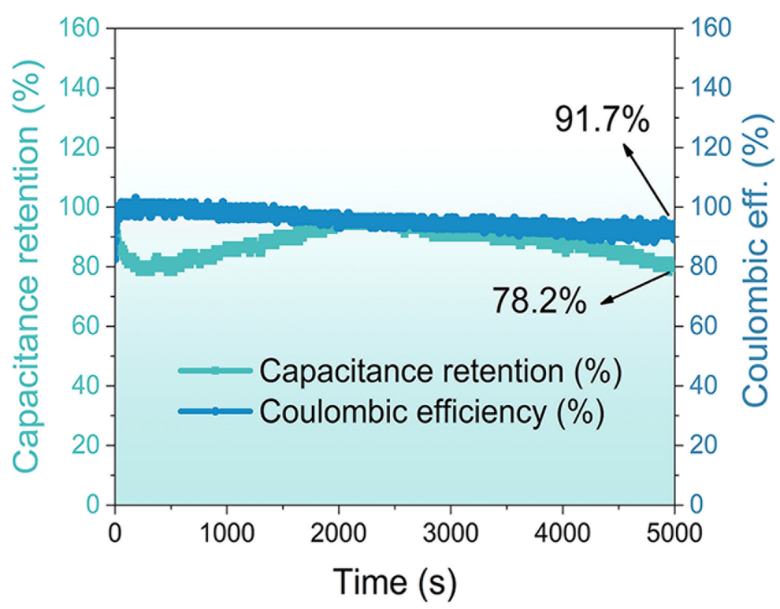


**Fig. S41** Cycling stability of the WNCS//NiCo ASC device at the current density of 20 mA cm^−2^ for 500 cycles.


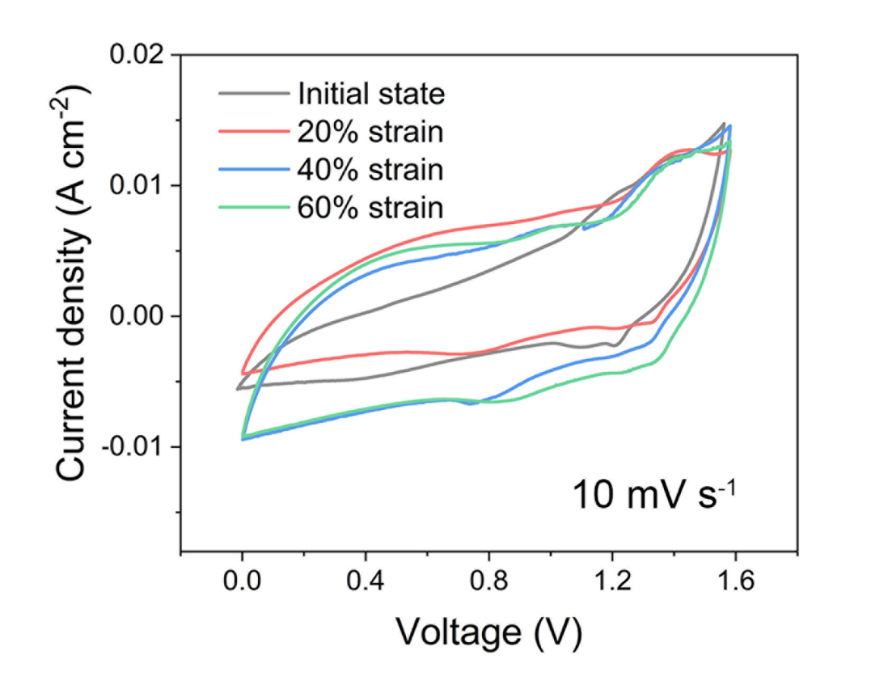


**Fig. S42** CV curves of the WNCS//NiCo ASC device under increasing compressive strains at 10 mV s^−1^.


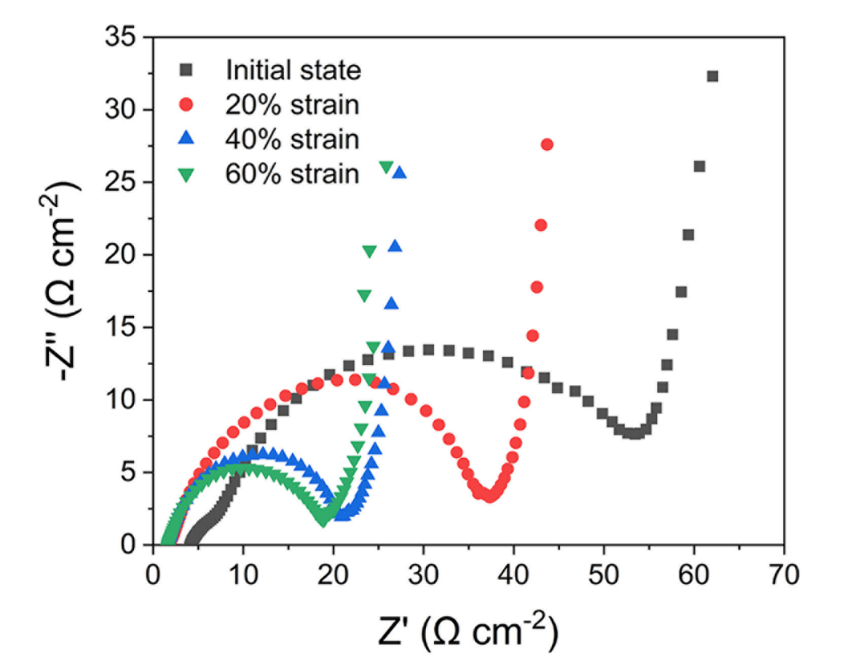


**Fig. S43** EIS plots of the WNCS//NiCo ASC device under increasing compressive strains.

**Supplementary Tables**

**Table S1** Comparison of the bulk density, BET surface area, average pore diameter, and pore volume of the WNCS with those of the natural wood, wood sponge, and wood carbon.

| Materials | Density  (mg cm^−3^) | SSA  (m^2^ g^−1^) | Average pore diameter | Pore volume  (cm^3^ g^−1^) | Micropore volume rate |
| --- | --- | --- | --- | --- | --- |
| Natural wood | 94.3 | 1.5 | 10.19 nm | 0.004 | 2% |
| Wood sponge | 34.2 | 6.7 | 12.83 nm | 0.022 | 0.1% |
| Wood carbon | 74.1 | 173.8 | 2.33 nm | 0.101 | 58.9% |
| WNCS | 42.6 | 601.7 | 2.18 nm | 0.328 | 71.9% |

**Table S2** Comparison of structural features and mechanical properties of the WNCS with other elastic 3D porous materials fabricated from different building blocks and methods.

| Material | Method | Morphology | Density  (mg cm^−3^) | Max strain | Stress  (kPa) | Fatigue resistance | | | | | Ref |
| --- | --- | --- | --- | --- | --- | --- | --- | --- | --- | --- | --- |
|  |  |  |  |  |  | Cycle | Strain | | Stress retention | |  |
| N-doped carbon foams | Carbonizing MF | 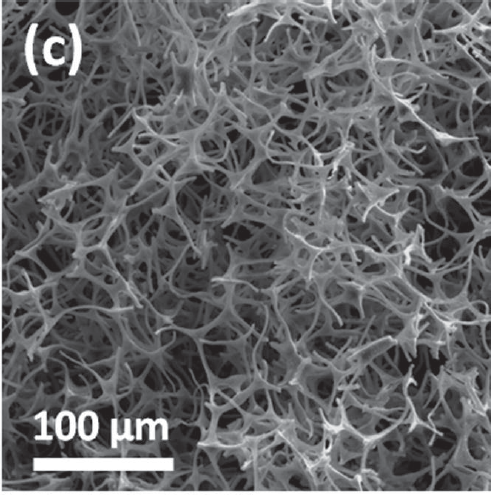 | - | 80% | 6.5 | 100 | | 55% | | - | [4] |
| MF/Ti_3_C_2_T_x_-50 | Coating on MF | 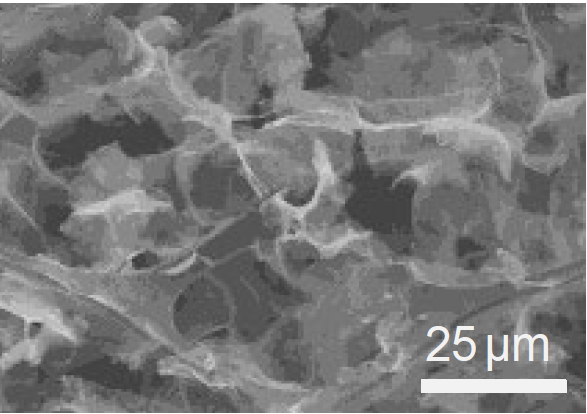 | - | 85% | 130 | 100 | | 50% | | >90% | [5] |
| PANI/CF | Carbonizing MF  Situ-polymerization | 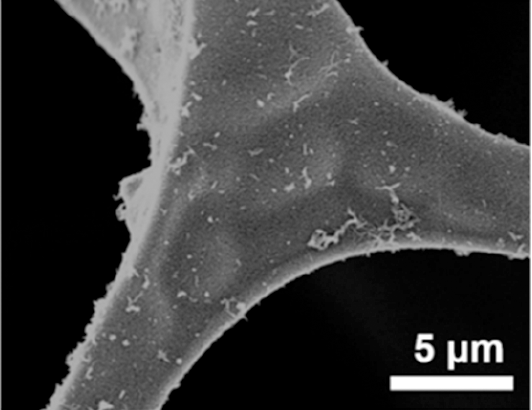 | - | 75% | 1.8 | - | | - | | - | [6] |
| Zn/CF | Carbonizing MF  Electrochemical deposition | 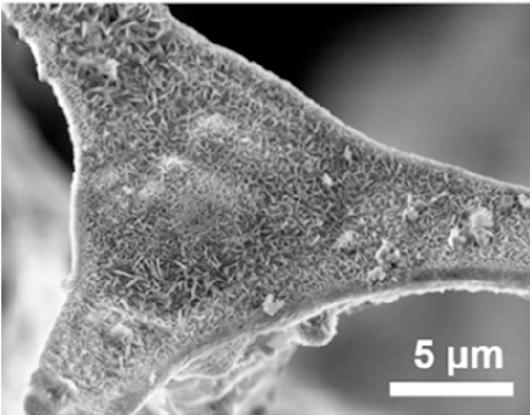 | - | 75% | 1.97 | - | | - | | - |  |
| MXene/rGO/  carbon foam | Coating on MF  Carbonization | 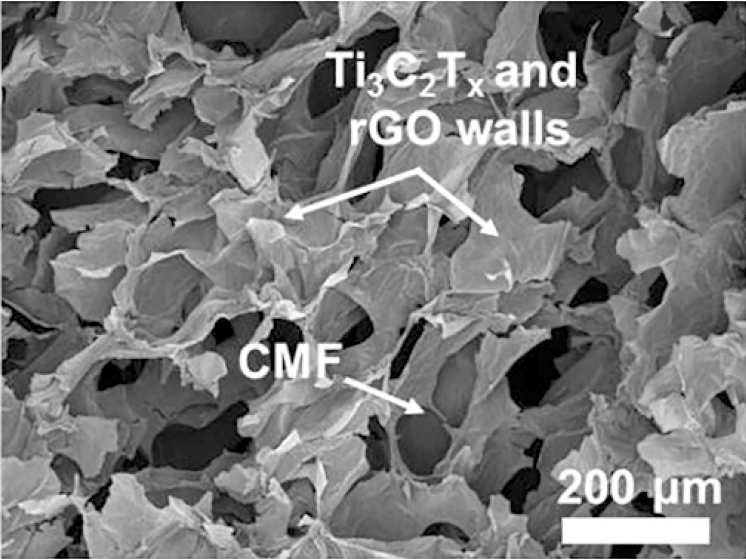 | - | 60% | 260 | 100 | | 50% | | <20% | [7] |
| PEDOT-PF^-^_6_ /NCF | Carbonizing MF  Situ-polymerization | 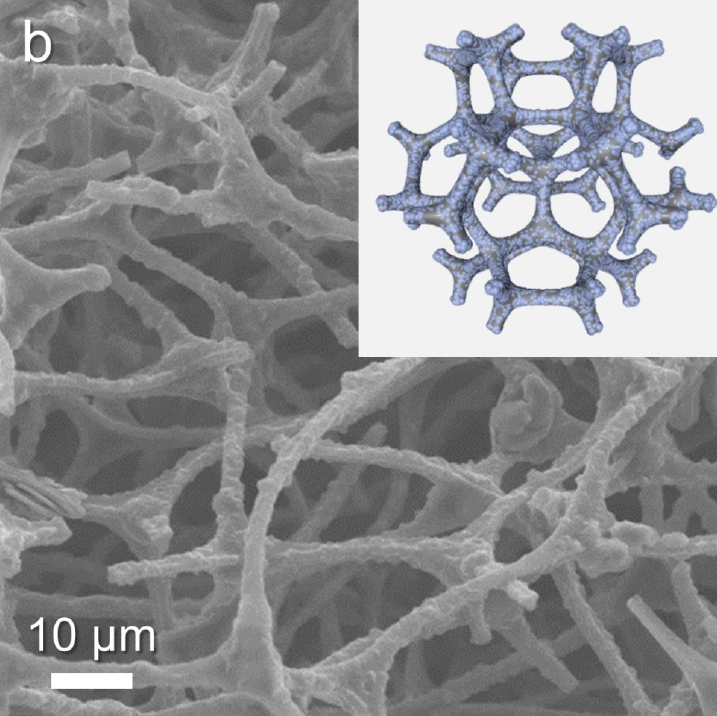 | - | 80% | ≈29 | 1000 | | 80% | | 85.1% | [8] |
| CNF/CNT/RGO carbon aerogel | Freeze-casting | 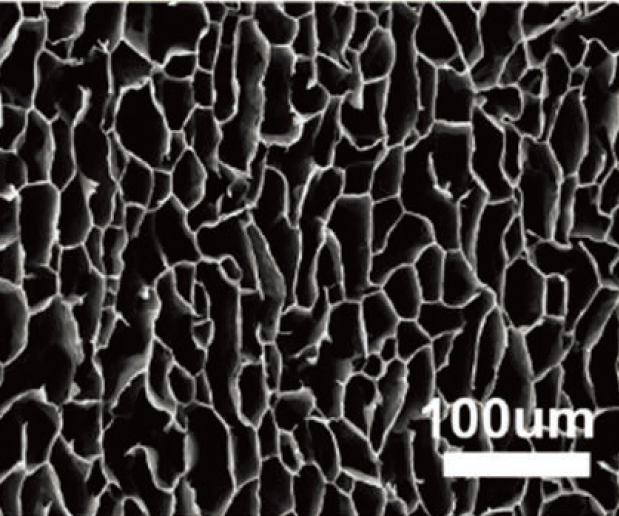 | 2.64 | 80% | 1.25 | 10000 | | 50% | | 85.3% | [9] |
| CNF/CNT/MXene aerogel | Freeze-casting | 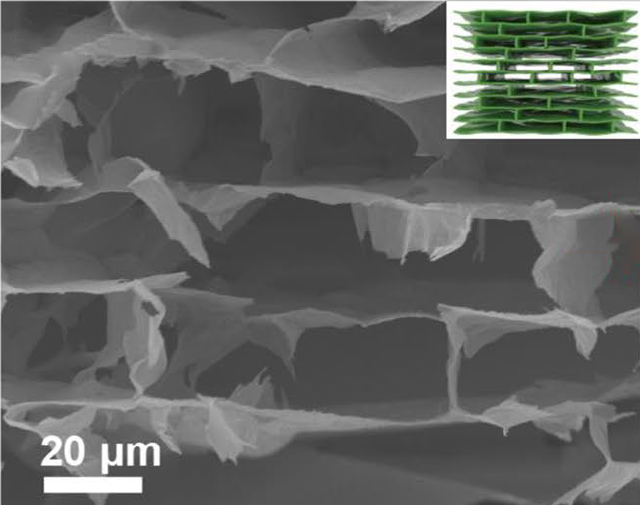 | 7.48 | 80% | 6.6 | 1000 | | 50% | | 90.3% | [10] |
| MX/PP/CNC | Freeze-casting | 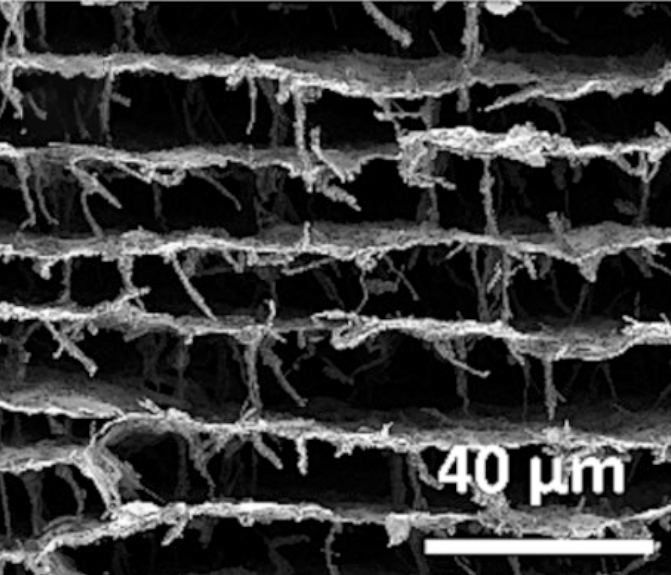 | 32.7 | 80% | 65 | 100 | | 50% | | 92.7 % | [11] |
| TCNF@SWCNT aerogel | 3D printing | 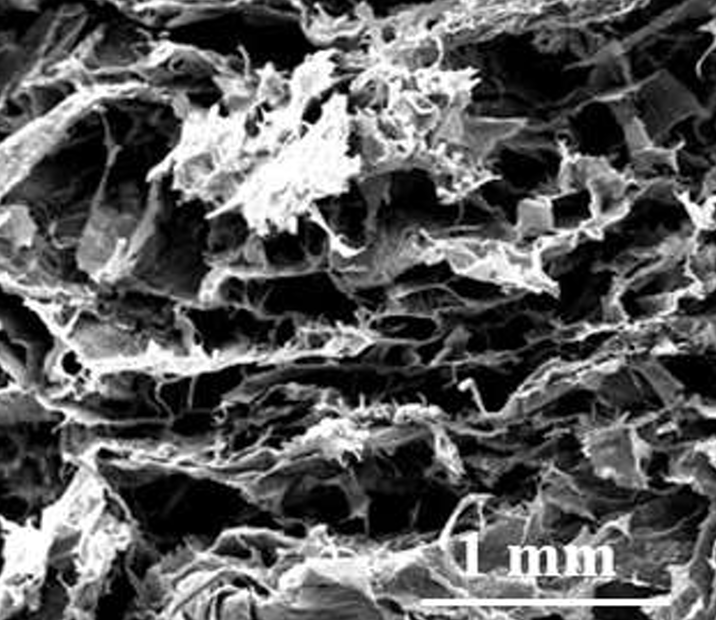 | 47 | 80% | 280 | 200 | | 50% | | - | [12] |
| rGO/CNTs | 3D printing | 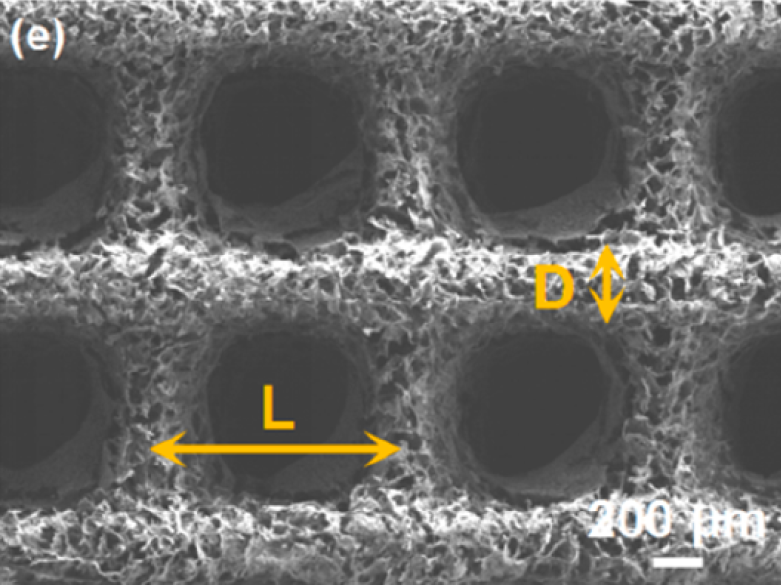 | 20 | 60% | 83 | - | | - | | - | [13] |
| 3DP-rGO foam | 3D printing | 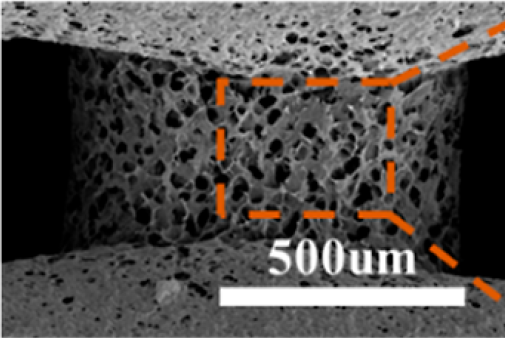 | 11.4 | 90% | 281.6 | 500 | | 50% | | - | [14] |
| CNTs aerogel | CVD | 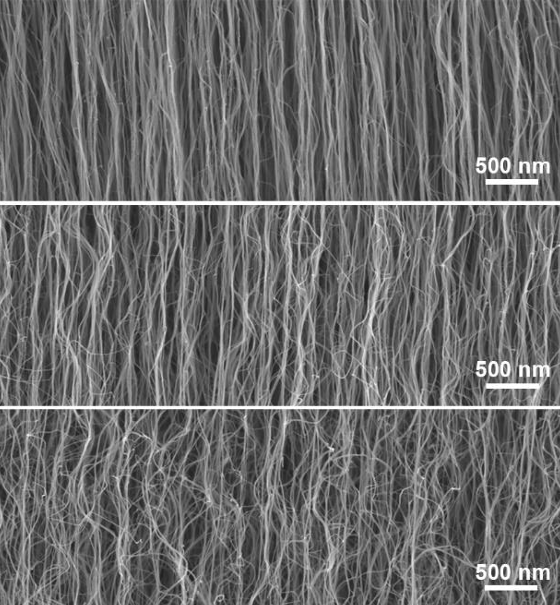 | - | 60% | 400 | 3000 | | 40% | | - | [15] |
| CNTs sponge | CVD | 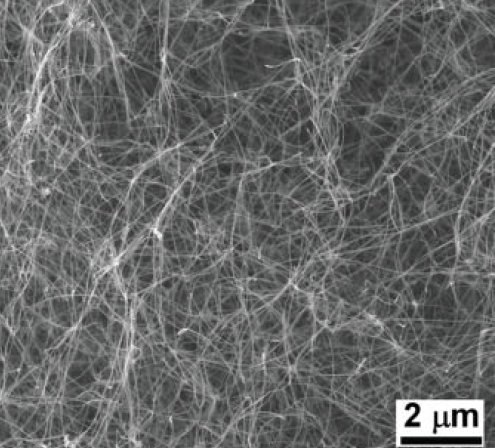 | 7.5 | 95% | <250 | 1000 | | 60% | | - | [16] |
| WNCS | Delignification  carbonization | 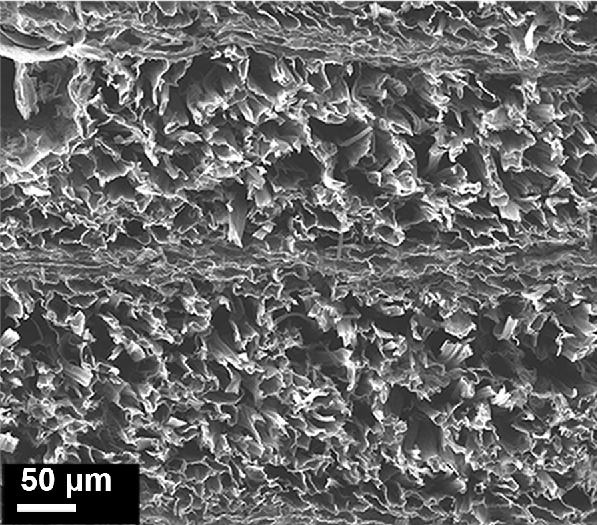 | 42.6 | 70% | 580.6 | 1000 | | 60% | | 75.2% | This work |

**Table S3** Comparison of electrochemical properties of the WNCS electrode with other reported EDL electrodes.

| Electrode materials | Potential | Electrolyte | Max specific capacitance | Cyclic stability | | | Ref. |
| --- | --- | --- | --- | --- | --- | --- | --- |
|  |  |  |  | *I* | Cycles | Capacitance  retention |  |
| CPYF-ZIF-67-PPy | 0 ~ 0.8 V | 1 M Na_2_SO_4_ | 2.3 F cm^−2^  (0.5 mA cm^−2^) | 10 mA cm^−2^ | 10,000 | 87.6% | [17] |
| CTAs@NCBs | 0 ~ 1 V | 1 M H_2_SO_4_ | 580 mF cm^−2^  (1 mA cm^−2^) | 20 mA cm^−2^ | 10,000 | 98.5% | [18] |
| NCNF2-900 | 0 ~ 1 V | 2 M H_2_SO_4_ | 612 mF cm^−2^  224 F g^−1^  (1.37 mA cm^−2^) | 5 A g^−1^ | 10,000 | 97% | [19] |
| Fe_2_O_3_/MXene aerogel | −1.1 ~ −0.3 V | 3 M H_2_SO_4_ | 691 mF cm^−2^  182 F g^−1^  (1 A g^−1^) | 5 A g^−1^ | 10,000 | 81.7% | [20] |
| MXene/SWCNT/CNF aerogel | 0 ~ 0.8 V | 2 M H_2_SO_4_ | 747 mF cm^−2^  (1 mV s^−1^) | - | - | - | [21] |
| MXene/carbon fibers | −0.3 ~ 0.7 V | 1 M H_2_SO_4_ | 1.7 F cm^−2^  (0.5 mA cm^−2^) | 6 mA cm^−2^ | 15,000 | 94% | [22] |
| N-doped graphene aerogel | −1 ~ 0 V | 6 M KOH | 123.6 mF cm^−2^  (0.5 mA cm^−2^)  111.2 F g^−1^  (0.2 A g^−1^) | 5 A g^−1^ | 10,000 | 83.2% | [23] |
| CNF/CNT/RGO carbon aerogels | 0 ~ 1 V | 1 M H_2_SO_4_ | 182 mF cm^–2^  116.3 F g^−1^  (0.1 A g^−1^) | 5 A g^−1^ | 5,000 | 92% | [9] |
| Ti_3_C_2_T_x_/rGO/Fe_3_O_4_ aerogel | −1.1 ~ −0.3 V | 1 M KOH | 1.25 F cm^−2^  (1 mA cm^−2^) | 20 mA cm^−2^ | 5,000 | 85.8% | [24] |
| Biomass carbon/RGO/CNF aerogel | 0 ~ 0.8 V | 1 M Na_2_SO_4_ | 1.6 F cm^−2^  (5 mA cm^−2^) | - | - | - | [25] |
| WNCS | −0.8 ~ 0 V | 3 M KOH | 4.2 F cm^−2^  11.7 F cm^−3^  176.3 F g^−1^  (1 mA cm^−2^) | 50 mA cm^−2^ | 10,000 | 100.2% | This work |

**Table S4** Comparison of electrochemical properties of supercapacitors based on 3D porous materials.

| 3D materials | Devices | Specific capacitance  (mF cm^−2^) | Energy density  (μW h cm^−2^) | Stability | Ref. |
| --- | --- | --- | --- | --- | --- |
| Wood | CDW/MoSe_2_||CDW/MnO_2_ | 415 (2.5 mA cm^−2^) | 147 (2 mW cm^−2^) | 80.9% retention after 1500 cycles | [26] |
|  | WC/MnO_2_ | 1120 (2 mA cm^−2^) | 502 (22.3 W kg^−1^) | 75.2% retention after  10,000 cycles | [27] |
|  | CW/Co(OH)_2_\|\|CW | 2200 (1 mA cm^−2^) | 690 (1.1 mW cm^−2^) | 85% retention after  10,000 cycles | [28] |
|  | CWZ/ZIF-67 | 732.5 (5 mA cm^−2^) | 107 (5 mW cm^−2^) | 74.2 % retention after 10,000 cycles | [29] |
|  | Ni-Ti_3_C_2_/Wood | 490 (2 mA cm^−2^) | 23 (557 μW cm^−2^) | 87 % retention after 5000 cycles | [30] |
|  |  |  |  |  |  |
| Cellulose | CNF/CNT/RGO | 109.4 (0.4 mA cm^−2^) | 15.2 (432 μW cm^−2^) | 85% retention after 10000 cycles | [9] |
|  | MXene/CNF | 849.2 (0.8 mA cm^−2^) | 21.2 (240 μW cm^−2^) | 88% retention after 10,000 cycles | [10] |
|  | C-AL/CNF | 231 (0.5 mA cm^−2^) | 32.1 (256 μW cm^−2^) | 88.5 % retention after 5,000 cycles | [31] |
|  |  |  |  |  |  |
| None-reneweable material | PPy/APA | 831 (6.2 mA cm^−2^) | 73.8 (570 μW cm^−2^) | 86.5% retention after 1000 cycles | [32] |
|  | HAN/SnO_2_/MnO_2_\|\|HAN/  SnO_2_/PPy | 128 (0.5 mA cm^−2^) | 160 (40 mW cm^−2^) | 87% retention after 30,000 cycles | [33] |
|  | MXene-AgNW-MNOW-C60 | 216.2 (10 mV s^−1^) | 19.2 (58.3 mW cm^−2^) | 85 % retention after 10,000 cycles | [34] |
|  | 3D-GA\|\|3D-GA-MnO_2_ | 1700.6 (5 mA cm^−2^) | 650 (164.5 mW cm^−2^) | 93% retention after 100,000 cycles | [35] |
|  |  |  |  |  |  |
| WNCS | WNCS||NiCo LDH/CFs | 1791.5 (2 mA cm^−2^) | 607.1 (1.56 mW cm^−2^) | 78.2% retention after 5000 cycles | This work |

Note: CDW, carbonized delignified wood; WC, wood carbon; CWZ, carbonized wood@ZIF-8; C-AL, carbon aerogel lignin; APA, aligned polyacrylamide aerogel; HAN, honeycomb alumina nanoscaffold; MNOW, manganese dioxide nanowires; 3D-GA, 3D-printed graphene aerogel.

**Table S5 Laboratory-scale WNCS fabrication cost calculation.**

| Items | Cost | Dosage/Consumption | Expense  (￥) |
| --- | --- | --- | --- |
| Balsa wood | 20￥/2×2×50 cm | 2×2×16 cm | 6.4 |
| NaOH | 50￥/500 g | 40 g | 4 |
| NaClO_2_ | 82￥/500 g | 10 g | 1.64 |
| Acetic acid | 60￥/500 mL | ≈1 mL | 0.12 |
| Nitric acid | 208￥/500 mL | 15 mL | 6.24 |
| Ethanol | 54￥/L | 200 mL | 10.8 |
| Tert butyl alcohol | 46￥/500 mL | 150 mL | 13.8 |
| Power | 2.7￥/kWh | 8 kWh | 21.6 |

Due to the inexpensive balsa wood feedstock and the mild manufacturing process, the lab-scale production cost of WNCS is calculated at only 4.5￥/cm³, equivalent to 105.5 ￥/g, which is notably lower than that of graphene, MXene, and activated carbon products. With industrial-scale production, this cost could be further reduced.

**Supplementary References**

[1] S. Wei, C. Wan and Y. Wu, *Green Chem.*, **2023**, 25, 3322.

[2] H. Dong, X. Li, Z. Cai, S. Wei, S. Fan, Y. Ge, X. Li and Y. Wu, *Small*, **2023**, e2305857.

[3] M. Borrega, P. Ahvenainen, R. Serimaa and L. Gibson, *Wood Sci. Technol.*, **2015**, 49, 403.

[4] K. Xiao, L. X. Ding, G. X. Liu, H. B. Chen, S. Q. Wang and H. H. Wang, *Adv. Mater.*, **2016**, 28, 5997.

[5] R. Guo, X. Y. Han, P. Yuan, X. X. He, Q. Li, J. Sun, L. Q. Dang, Z. H. Liu, Y. T. Zhang and Z. B. Lei, *Nano Res.*, **2022**, 15, 3254-3263.

[6] D. T. Li, T. Lv, Z. L. Chen, Y. L. Yang, Y. A. Liu, J. Wan, Y. L. Qi, S. K. Cao and T. Chen, *Small Struct.*, **2022**, 3, 2200027.

[7] J. Zhang, D. Jiang, L. Liao, L. Cui, R. Zheng and J. Liu, *Chem. Eng. J.*, **2022**, 429. 132232.

[8] B. H. Xiao, J. X. Li, H. Y. Xu, J. L. Huang, Y. L. Luo, K. Xiao and Z. Q. Liu, *Angew. Chem. Int. Ed.*, **2023**, 62, e202309614.

[9] H. Y. Liu, T. Xu, C. Y. Cai, K. Liu, W. Liu, M. Zhang, H. S. Du, C. L. Si and K. Zhang, *Adv. Funct. Mater.*, **2022**, 32, 2113082.

[10] T. Xu, Q. Song, K. Liu, H. Liu, J. Pan, W. Liu, L. Dai, M. Zhang, Y. Wang, C. Si, H. Du and K. Zhang, *Nanomicro Lett.*, **2023**, 15, 98.

[11] H. Huang, X. Ning, T. Yuan, C. Li, X. Zuo, Y. Zhang, Z. Chen, C. Sun, H. Zhang, Z. Fan and L. Pan, *J. Energy Storage*, **2024**, 83, 110676.

[12] W. Kang, L. Zeng, S. Ling, C. Lv, J. Liu, R. Yuan and C. Zhang, *Adv. Funct. Mater.*, **2021**, 31, 2102184.

[1] D. Kong, Y. Wang, S. Huang, B. Zhang, Y. V. Lim, G. J. Sim, Y. A. P. Valdivia, Q. Ge and H. Y. Yang, *ACS Nano*, 2020, 14, 9675.

[14] W. Cao, S. Ling, H. Chen, H. He, X. Li and C. Zhang, *Ind. Eng. Chem. Res*, **2022**, 61, 10922-10930.

[15] Y. Zhao, J. Cao, Y. Zhang and H. Peng, *Adv. Funct. Mater.*, **2019**, 30, 1902971.

[16] X. Gui, J. Wei, K. Wang, A. Cao, H. Zhu, Y. Jia, Q. Shu and D. Wu, *Adv. Mater.*, **2010**, 22, 617.

[17] Y. Liang, X. Luo, Z. Hu, L. Yang, Y. Zhang, L. Zhu and M. Zhu, *J. Colloid Interface Sci.*, **2023**, 631, 77.

[18] Z. Tang, G. Zhang, H. Zhang, L. Wang, H. Shi, D. Wei and H. Duan, *Energy Stor. Mater.*, **2018**, 10, 75.

[19] H. Chen, T. Liu, J. Mou, W. Zhang, Z. Jiang, J. Liu, J. Huang and M. Liu, *Nano Energy*, **2019**, 63, 103836.

[20] Y. Luo, Y. Tang, X. Bin, C. Xia and W. Que, *Small*, **2022**, 18, e2204917.

[21] S. Lyu, H. Chang, L. Zhang, S. Wang, S. Li, Y. Lu and S. Li, *Compos. B. Eng.*, **2023**, 264.

[22] W. Fan, Q. Wang, K. Rong, Y. Shi, W. Peng, H. Li, Z. Guo, B. B. Xu, H. Hou, H. Algadi and S. Ge, *Nanomicro Lett.*, **2023**, 16, 36.

[23] X. Xiang, Z. Deng, H. Zhang, C. Gao, S. Feng, Z. Liu, Q. Liang, Y. Fu, Y. Liu and K. Liu, *Chem. Eng. J.*, **2023**, 477, 147211.

[24] L. Zhang, K. Yu, Y. Li, Z. Wang, K. Zhan, J. Yang and B. Zhao, *ACS Appl. Nano Mater.*, **2022**, 6, 482.

[25] R. Chen, X. Li, Q. Huang, H. Ling, Y. Yang and X. Wang, *Chem. Eng. J.*, **2021**, 412, 128755.

[26] S. Wei, C. Wan, Y. Jiao, X. Li, J. Li, Y. Wu, *Chem. Commun.* **2020**, 56, 340.

[27] L. Chen, F. Wang, Z. Tian, H. Guo, C. Cai, Q. Wu, H. Du, K. Liu, Z. Hao, S. He, G. Duan, S. Jiang, *Small* **2022**, 18, e2201307.

[28] Y. Wang, X. Lin, T. Liu, H. Chen, S. Chen, Z. Jiang, J. Liu, J. Huang, M. Liu, *Adv. Funct. Mater.* **2018**, 28.

[29] Z. Zhang, S. Deng, D. Wang, Y. Qing, G. Yan, L. Li, Y. Wu, *Chem. Eng. J.* **2023**, 454.

[30] W. Chen, K. Yang, M. Luo, D. Zhang, Z. Li, C. Liu, X. Zhou, *EcoMat* **2022**, 5.

[31] Z. H. Chen, H. Zhuo, Y. J. Hu, H. H. Lai, L. X. Liu, L. X. Zhong, X. W. Peng, *Adv. Funct. Mater.* **2020**, 30.

[32] Y. Zhao, Y. Alsaid, B. Yao, Y. Zhang, B. Zhang, N. Bhuskute, S. Wu, X. He, *Adv. Funct. Mater.* **2020**, 30.

[33] Z. Lei, L. Liu, H. Zhao, F. Liang, S. Chang, L. Li, Y. Zhang, Z. Lin, J. Kroger, Y. Lei, Nat. *Commun.* **2020**, 11, 299.

[34] X. Li, H. Li, X. Fan, X. Shi, J. Liang, *Adv. Energy Mater.* **2020**, 10.

[35] B. Yao, S. Chandrasekaran, H. Zhang, A. Ma, J. Kang, L. Zhang, X. Lu, F. Qian, C. Zhu, E. B. Duoss, C. M. Spadaccini, M. A. Worsley, Y. Li, *Adv. Mater.* **2020**, 32, e1906652.
